# RiPP recognition elements evolved to prevent pathway interference through leader peptide discrimination

**DOI:** 10.1038/s41467-026-73250-6

**Published:** 2026-05-20

**Authors:** Aleksandr Popov, Dmitry Bikmetov, Anastasiia Grigoreva, Marina Serebryakova, Konstantin Severinov, Yuri I. Wolf, Guy Lippens, Akira Wada, Shunsuke Tagami, Svetlana Dubiley

**Affiliations:** 1 RIKEN Center for Integrative Medical Sciences, Yokohama, Japan; 2 Graduate School of Medical Life Science, Yokohama City University, Yokohama, Japan; 3 Institute of Gene Biology, Russian Academy of Science, Moscow, Russia; 4 A.N. Belozersky Institute of Physicochemical Biology MSU, Moscow, Russia; 5 Division of Intramural Research, Computational Biology Branch, National Library of Medicine, National Institutes of Health, Bethesda, MD USA; 6 Toulouse Biotechnology Institute, CNRS/INRAE/INSA/UPS, Toulouse, France

**Keywords:** Post-translational modifications, Peptides, Biosynthesis

## Abstract

Ribosomally synthesized and post-translationally modified peptides (RiPPs) are natural products with diverse structures and functions. Here, we report the discovery of a family of RiPPs whose biosynthetic gene clusters are widespread in the Bacillota genomes and often co-localize with those of lasso peptides, another distinct family of RiPPs. The synthesis of both kinds of RiPPs relies on specific interactions between small adapter protein domains known as RiPP recognition elements (RREs) with their precursor peptides. As these latter share a conserved RRE-binding motif, conflicts between the two biosynthetic pathways may emerge. Through biochemical and structural studies, we reveal how the two RiPP biosynthetic systems evolved to discriminate between their cognate precursors and leader peptidases, allowing them to coexist within a single host. Thus, our study provides insights into the evolutionary diversification of RiPP families.

## Abstract (web summary)

One bacterium can produce many ribosomally synthesized and post-translationally modified peptides (RiPPs) from different families. Here, the authors show how two closely related RiPP families in Bacillota co-evolved to exclude non-cognate precursor peptides, preventing biosynthetic pathway interference.

## Introduction

Ribosomally synthesized and post-translationally modified peptides (RiPPs) are a vast group of structurally diverse natural products synthesized by microorganisms, fungi, plants, and animals^1^. RiPPs defend against biotic^2^ and abiotic stresses^3^, mediate bacterial communication^4,5^, regulate morphogenesis^6,7^, and contribute to nutrient^8^ and trace metal acquisition^9^. A single organism may produce multiple RiPPs with non-overlapping functions^10,11^. However, the function of many RiPPs remains elusive^12^.

RiPPs are synthesized from genetically encoded precursor peptides that undergo biochemical transformations by dedicated tailoring enzymes^13^. Many tailoring enzymes display relaxed substrate specificity, tolerating mutations in the cognate precursor or even modifying dissimilar peptide sequences^14–16^. Although broad substrate tolerance of tailoring enzymes promotes rapid evolution of RiPPs, it also creates challenges and may lead to off-target modification of non-cognate RiPP precursors as well as cellular peptides and proteins.

**Fig. 1:**
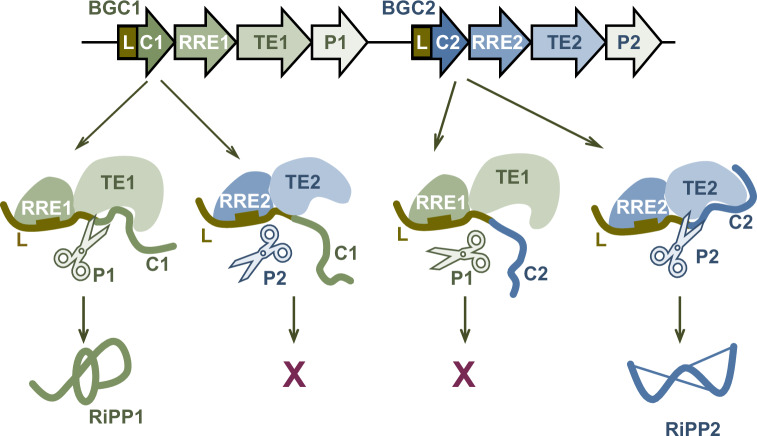
Potential conflict between two RiPP biosynthetic pathways in the processing of precursor peptides bearing identical RRE-binding motifs. RiPP1 and RiPP2, ribosomally synthesized and post-translationally modified peptides; BGC1 and BGC2, RiPP biosynthetic gene clusters; L, leader region of the precursor peptide containing a conserved RRE-binding motif; C1 and C2, core regions of the precursor peptides; RRE1 and RRE2, RiPP recognition elements; TE1 and TE2, tailoring enzymes; P1 and P2, leader peptidases.

To reduce the risk of off-target modifications, RiPP biosynthesis systems often rely on the modular structure of the precursor peptides. A typical precursor peptide consists of a C-terminal core, where post-translational modifications (PTMs) are installed, and a cleavable N-terminal leader^17^. The leader acts as a bait for a specialized domain known as the RiPP recognition element (RRE)^18^. The RRE serves as an adapter that brings together the tailoring enzymes and the cognate precursor presented in a modification-competent conformation (Fig. 1).

RREs are encoded in nearly 50% of all known RiPP biosynthetic gene clusters (BGCs), typically as domains within tailoring enzymes that catalyze the primary modification of the precursor^18^. In a substantial number of BGCs, however, RREs are present as stand-alone proteins (discrete RREs). Most lasso peptides, a large family of RiPPs characterized by a unique lariat-knot topology^19^, depend on discrete RREs in their biosynthesis^20^. The RREs deliver the precursor peptide to a specialized leader peptidase^21^, facilitate macrocyclization of the backbone by the lasso macrolactam cyclase^22^, and, in some cases, assist in the installation of additional modifications, such as phosphorylation^23^ or hydroxylation^24^. Most discrete lasso peptide RREs specifically recognize leader peptides harboring the consensus motif [Y/W]xxPx[L/V/I/A/F/Y]^25^. Similar to other RiPP families^26,27^, lasso peptide BGCs can encode multiple precursor peptides with divergent core sequences but identical leader motifs^25,28^, which may lead to competition among the precursors for the shared modification machinery. Notably, the lasso-like RRE-binding motif is also present in leaders of pyrroloquinoline quinone (PQQ) precursor peptides, a distinct family of small hypermodified RiPPs that are structurally unrelated to lasso peptides^25,29^. Both lasso peptide and PQQ RiPP families are broadly distributed in bacteria from overlapping phylogenetic groups^30,31^ and could therefore co-occur within a single genome. Simultaneous expression of PQQ and lasso peptide BGCs within a single cell could lead to cross-recognition of non-cognate precursors by enzymes from the other family, resulting in nonproductive complexes and reduced biosynthetic efficiency (Fig. 1). The strategies by which distinct RiPP biosynthetic pathways prevent or tolerate this interference remain unexplored.

In this paper, we address this question by combining biochemical and structural analyses of RREs and leader peptides interactions from two evolutionarily close RiPP families. We describe a family of RiPPs whose precursor peptides share an RRE-binding motif with that of some lasso and PQQ peptides. Although the final structures of these RiPPs remain undetermined, we refer to them here, for convenience, as Linear Polyphosphorylated Peptides (LPPs), based on the structure of their biosynthetic intermediates; this designation is not intended as a formal nomenclature proposal. We found that LPP and paeninodin-like lasso peptide BGCs often co-localize in the genomes of Bacilli. We demonstrate that, despite the similarity of the biosynthetic enzymes and the sequences of the LPP and lasso peptide leaders, efficient RiPP biosynthesis is ensured through selective recruitment of cognate precursors by their respective RRE domains. We elucidate the molecular mechanisms underlying the specificities of LPP and lasso peptide RREs. Finally, we identify a homolog of SipW, a specialized signal peptidase from Bacilli, as the enzyme responsible for the RRE-dependent cleavage of the leader peptide in LPP biosynthesis.

## Results

### Discovery of a RiPP family guided by archaeal-type signal peptidase

**Fig. 2:**
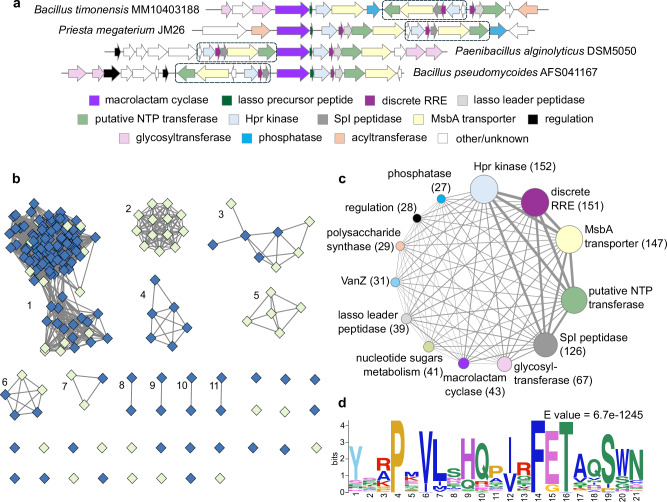
Bioinformatic analysis of SpI-encoding RiPP biosynthetic gene clusters. **a** Representative lasso peptide BGCs and their genetic neighborhoods. Genes are shown as arrows and colored according to predicted functional annotation. Putative linear polyphosphorylated peptide (LPP) BGCs are highlighted with frames. **b** Sequence similarity network (SSN) of archaeal-type signal peptidases I (SpI) homologous to WP_029196374.1 from *P. alginolyticus* DSM5050. Blue nodes denote SpI proteins whose genes are co-localized with the RRE-encoding gene. Connected components (clusters) are numbered by size. **c** Co-occurrence network of protein domains encoded within 12 kb of SpI genes from SSN cluster 1 (Supplementary Data 2). Line thickness reflects the frequency of co-occurrence. The total number of domains per node is indicated in parentheses and represented by node size. **d** Sequence Logo of the conserved motif found in putative LPP precursors.

During a search for lasso peptide biosynthetic genes in Bacilli genomes, we identified a group of atypical BGCs embedded within capsular polysaccharide biosynthesis loci. These clusters encode the usual lasso peptide biosynthetic machinery, including a precursor peptide (protein A) and a macrolactam cyclase (protein C), along with other biosynthetic proteins common in the paeninodin-like lasso peptide BGCs^32,33^. Unexpectedly, in addition to the canonical lasso leader peptidase (protein B2), they also carried two copies of the RREs and an archaeal-type signal peptidase I (SpI) homologous to *Bacillus subtilis* SipW (Fig. 2a). SpI-like peptidases have not previously been described in RiPP biosynthetic pathways^34^, making these BGCs both unusual and intriguing.

To explore the distribution of SpI-like peptidases in RiPP BGCs, we collected close homologs of the *Paenibacillus alginolyticus* DSM5050 SpI (WP_029196374.1) from the NCBI protein database using BLASTP search^35^ with an E-value cutoff of 0.001 (Supplementary Data 1). After filtering out sequences that were located on the edge of a contig, the final dataset yielded 199 proteins. These proteins were then used to construct a sequence similarity network (SSN). Removal of edges with an E-value more than 10E-23 from the SSN resulted in 11 clusters (connected components) comprising two or more members (Fig. 2b), with most of the hits originating from the Bacillota phylum (Supplementary Fig. 1).

Genomic context analysis using the RODEO web tool^31^ revealed that some nodes represent housekeeping enzymes, such as the bona fide SipW signal peptidases^36,37^, included in the SSN cluster #2. However, approximately 75% of collected SpIs co-localize with discrete RREs (Fig. 2b), which are the hallmarks of RiPP BGCs. The *P. alginolyticus* SpI-like peptidase, together with most other RRE-associated SpIs, falls within the largest SSN cluster #1, which comprises 126 members predominantly from Bacillota genomes. We therefore focused our study on this SSN cluster.

Defining the boundaries of unknown BGC types is often a challenging task, especially when gene clusters are embedded within loci rich in glycosyltransferases^38^ and other potential PTM-installing enzymes. We calculated the frequency of paired co-occurrence of protein domains, which allowed identification of conserved core genes defining these BGCs (Supplementary Data 2). The analysis revealed a conserved set of genes encoding SpI, a discrete RRE, an HprK-like kinase, an uncharacterized NTP transferase, and an MsbA-like transporter (Fig. 2c). HprK kinases and NTP transferases are typical secondary modification enzymes in paeninodin-like lasso peptide biosynthesis^33^. However, only about one-third of SpIs were linked to genes encoding canonical lasso biosynthetic enzymes such as macrolactam synthase and leader peptidase B2 (Supplementary Data 2). These results suggest that SpIs are part of a family of RiPP BGCs that often co-localize with the paeninodin-like lasso peptide BGCs. Notably, co-occurrence analysis revealed that BGCs of this family and paeninodin lasso peptides are often present within the same *Bacilli* genome, suggesting a possible synergistic relationship between the two RiPP systems. The set of biosynthetic enzymes shared with the paeninodin-type lasso peptides^32^ indicates that these putative RiPPs represent phosphorylated and glycosylated peptides, whereas the absence of a macrolactam cyclase suggests they remain linear. For convenience, we hereafter refer to this RiPP family as Linear Polyphosphorylated Peptides (LPPs).

A manual search for genes of putative precursor peptides in LPP BGCs revealed one to three open reading frames encoding 35–200 amino acid-long proteins per BGC. The precursors harbor a well-conserved N-terminal region but have highly variable C termini. Remarkably, the consensus N-terminal sequence contains a YxxPxVLxHQxIxFETxxS motif (Fig. 2d), part of which strongly resembles the [Y/W]xxPx[L/V/I/F/A] motif recognized by discrete lasso peptide RREs^25^. The frequent colocalization of the two BGC types in the same genome raises an intriguing functional question: do biosynthetic enzymes and RREs distinguish between the similar leader motifs to prevent potential conflict?

### Heterologous expression of *P. alginolyticus* DSM5050 *lpp* BGC

Conflicts arising from cross-interactions between distinct RiPP biosynthetic pathways may be mitigated by differential timing of expression^39^, nonproductive RRE•precursor•enzyme complex formation^40^, or other regulatory mechanisms. Before examining possible interference between the LPP and paeninodin biosynthetic pathways, we first sought to validate the bioinformatic prediction supporting the existence of the proposed LPP family. For this, we selected *P. alginolyticus* DSM5050, which harbors adjacent *lpp* and paeninodin-like lasso peptide BGCs in the genome. First, we asked if the native host expresses the putative *lpp* under laboratory conditions. RT-qPCR revealed a modest up-regulation of *lppB* (PAL01S_RS16300), which encodes the discrete RRE domain, during the stationary phase of growth (p-value 1.57E-03) (Supplementary Fig. 2). Expression of the adjacent lasso peptide *pba* BGC (*pbaB1*, PAL01S_RS16265) and of the exopolysaccharide biosynthesis-related *espG* (PAL01S_RS16325)^41^ was synchronously up-regulated (p-values 3.42E-04 and 9.25E-07, respectively) (Supplementary Figs. 2, 3). However, mass spectrometric analysis of *P. alginolyticus* DSM5050 cells and spent medium did not reveal mass ions we could attribute to putative products of the *pba* or *lpp* BGCs. We note, however, that since *pba* and *lpp* BGCs are surrounded by numerous genes related to polysaccharide biosynthesis Supplementary Fig. 3), which could contribute to peptide modification^38^, the complete set of the secondary PTMs in these RiPPs cannot be reliably predicted, thereby complicating their identification by mass spectrometry.

**Fig. 3:**
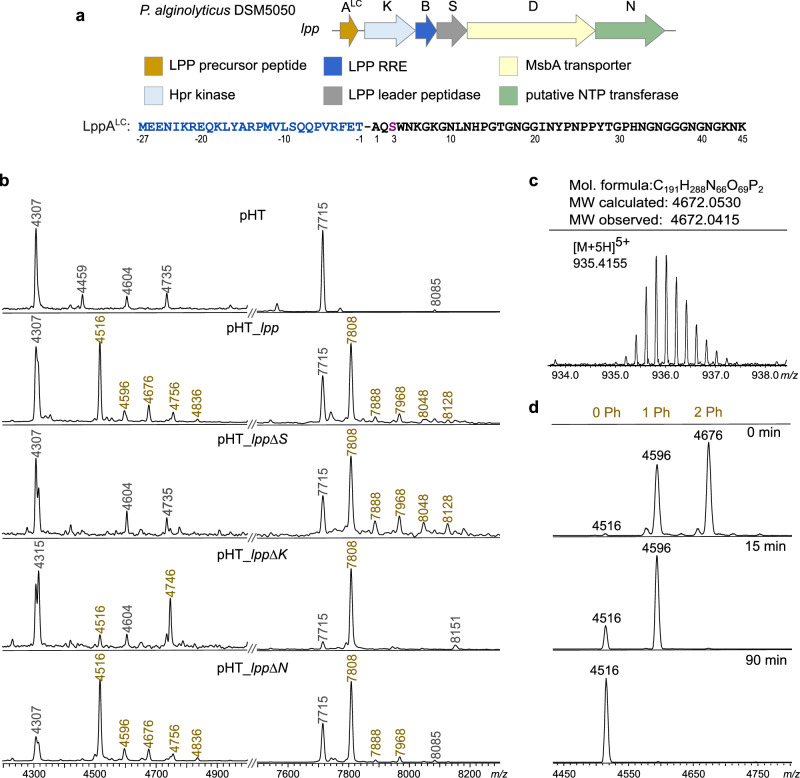
Multiple phosphorylation is the primary modification in LPP. **a** Architecture of the *P. alginolyticus* DSM550 *lpp* core BGC and sequence of the LppA^LC^ precursor peptide. Leader peptide residues are numbered from −27 to −1, and core peptide residues are numbered 1 through 45. The leader sequence and modified S3 residue are highlighted in blue and violet, respectively. **b** MALDI-TOF MS analysis of *B. subtilis* expressing *lpp* BGC variants. [M + H]^+^_av_ at *m/z* 7808, 7888, 7968, 8048, and 8128 are assigned to unmodified LppA^LC^ and its mono-, di-, tri-, and tetra-phosphorylated forms, respectively. Signals at *m/z* 4516, 4596, 4676, 4756, and 4831 correspond to leader-cleaved LppA^C^ and its mono-, di-, tri-, and tetra-phosphorylated forms, respectively. **c** High-resolution MS spectrum of the di-phosphorylated LppA^C^ core from *B. subtilis* wild-type *lpp* BGC. **d** CIAP treatment abolishes phosphorylation in *lpp* BGC products from the heterologous host.

To determine primary modifications that define the LPP family, we set up an expression system of a putative operon containing the core genes of the *lpp* BGC in the *B. subtilis* 168 *rok*::*erm*^42^ heterologous host. We constructed a pHT01-derived plasmid harboring genes coding for the precursor peptide LppA^LC^ (where L and C denote the leader and core part of the peptide, respectively), HprK kinase LppK, RRE domain LppB, SpI-like peptidase LppS, export pump LppD, and NTP-transferase LppN (Fig. 3a) under an IPTG-inducible promoter. Comparative MALDI MS analysis of the *B. subtilis* cells carrying the pHT-*lppA*^*LC*^*KBSDN* and empty vectors identified an [M + H]^+^_av_ at *m/z* 7808 matching the LppA^LC^ peptide and a series of additional [M + H]^+^_av_ ions with mass increments of 80 Da (Fig. 3b, two top right panels), which conform to LppA^LC^ bearing 1 to 4 phosphate groups. A similar series of mass ions with 80-Da mass shifts observed in the lower *m/z* part of the spectrum (Fig. 3b, two top left panels) could correspond to the C-terminal part of the precursor peptides cleaved after  the T-1 residue (Fig. 3a). The measured monoisotopic masses of the putative processed products, [M + 4H]^4+^_mi_ 1129.0371 (Supplementary Fig. 4) and [M + 5H]^5+^_mi_ 935.4155 (Fig. 3c), matched the calculated masses of the 45-amino acid long C-terminal fragment of LppA^LC^ and its di-phosphorylated form, respectively, within 2.5 ppm accuracy.

We analyzed the products of *lpp* BGCs harboring single gene deletions to reveal the role of putative modification enzymes in LPP maturation. Deletion of *lppS* resulted in the accumulation of the phosphorylated precursor peptides, while the mass ions corresponding to the processed peptide disappeared (Fig. 3b, third panels from the top). Thus, SpI-like LppS peptidase is responsible for LppA^LC^ processing. Consistent with the bioinformatic prediction, cells lacking *lppK* produced only unphosphorylated precursor peptide (Fig. 3b, second panels from the bottom). Interestingly, the mass ion [M + H]^+^_av_ 4516, corresponding to unphosphorylated processed peptide, was barely detected in the *lpp*Δ*K* mutant cells, suggesting that the LppS peptidase processes the unphosphorylated precursor inefficiently. Conversely, a peak at *m/z* 4746 [M + H]^+^ accumulated, consistent with the peptide core extended by the Glu2–Lys45 proteolytic fragment of LppA. Deletion of *lppN* did not change the pattern of detectable RiPP products (Fig. 3b, bottom panels), suggesting that this enzyme is inactive in our heterologous expression system. We speculate that the LppN activity relies on a partner protein encoded outside the core BGC. The putative partner could either produce a rare substrate or introduce an additional secondary modification of phosphorylated LppA^LC^, which is a prerequisite for the LppN function.

We fractionated the cell lysate and treated the partially purified processed products of the *lpp* BGC with calf intestinal alkaline phosphatase (CIAP) to verify the PTM prediction. As expected, this treatment converted the [M + H]^+^_av_ 4596 and [M + H]^+^_av_ 4676 mass ions into a single species with [M + H]^+^_av_ 4516 (Fig. 3d). Taken together, these data indicate that the primary product of the LppA^LC^ modification is a linear peptide bearing multiple phosphate groups. Next, we shortened the phosphorylated products of the *lpp* BGC by treating them with LysC endopeptidase to facilitate identification of the LppA^LC^ phosphorylation site. The MALDI-TOF MS analysis of the digested peptides revealed that the phosphate groups are located in the N-terminal AQSNWK fragment of the LppA^C^ (Supplementary Fig. 5a). The following MS/MS analysis of the [M + H]^+^_mi_ ion at 813.3, matching the monophosphorylated peptide fragment, localized a phosphate group to the S3 residue (Supplementary Fig. 5b). To verify that the LppA^C^ S3 residue is the sole phosphorylation site, we generated LppA^LC^ mutants and analyzed their phosphorylation states. The wild-type LppA^LC^ carried two phosphate groups, whereas the S3A substitution abolished phosphorylation (Supplementary Fig. 6). No phosphorylation was detected for the S3T or S3Y LppA^LC^ variants. These results confirm that, similar to HprK-like kinases from paeninodin-like BGCs^32,33^, LppK transfers multiple phosphate groups onto the S3 residue.

Although we were unable to identify the final product, our data clearly show that the *lpp* BGC is functional. Collectively, our findings demonstrate that LppK-dependent phosphorylation of the S3 residue constitutes the primary post-translational modification in LPP. The precursor peptide is processed by the SpI-like leader peptidase LppS, resulting in the release of the modified 45-amino-acid core.

### Structural basis of precursor peptide recognition in LPP and paeninodin-like lasso peptide RREs

**Fig. 4:**
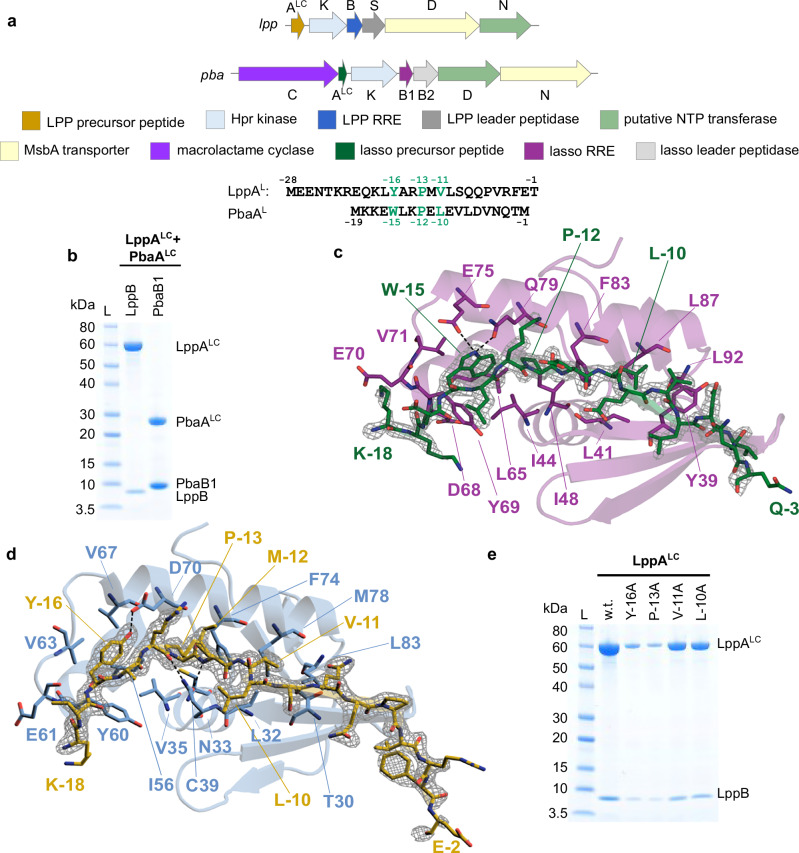
LppB and PbaB1 share a common mode of precursor peptide recognition. **a** Comparison of the *lpp* and *pba* BGCs. Conserved [Y/W]xxPx[V/L] motifs in the LppA^L^ and PbaA^L^ leader peptides are shown in teal. **b** Pull-down assays of RRE–precursor peptide complexes from *E. coli* co-expressing LppA^LC^ and PbaA^LC^ peptides with either Strep-tagged LppB or Strep-tagged PbaB1. LppA^LC^ was expressed as an MBP-LppA^LC^-TrxA fusion (MW 61.5 kDa), and PbaA^LC^ as a TrxA-PbaA^LC^-CBD fusion (MW 24.6 kDa). All pull-down experiments were performed independently three times with similar results. Source data are provided as a Source Data file. **c**,**d** Intermolecular interactions in the PbaB1•PbaA^L^ (pdb_00009x90) and LppB•LppA^L^ (pdb_00009x8z) complexes. PbaA^L^ and LppA^L^ peptides are shown in green and yellow, respectively. For the leader peptides, a sigma-A-weighted 2Fo–Fc map is contoured at 1.5 σ level with gray mesh. Hydrogen bonds between RRE and leader peptide side chains are indicated with dashed lines. Alternative conformations are shown for LppA^L^ M−12. **e** Pull-down assays of LppB in complexes with the wild-type and mutant LppA^LC^ peptides. L, protein MW ladder. All pull-down experiments were performed independently three times with similar results. Source data are provided as a Source Data file.

Our bioinformatics analysis showed that more than half of the bacteria harboring *lpp*-like BGCs also encode lasso peptide biosynthetic enzymes (Supplementary Data 1). This observation prompted us to test whether the RRE domains encoded in the adjacent *lpp* and lasso peptide BGCs of *P. alginolyticus* (Fig. 4a) can efficiently discriminate against non-cognate precursors. Since LppB was insoluble when expressed alone (Supplementary Fig. 7a), we decided to analyze the formation of RRE•precusor peptide complexes in an *E. coli* host (Supplementary Fig. 7b). Each Strep-tagged RRE domain was co-expressed with both precursor peptides simultaneously, and the protein complexes were isolated by affinity chromatography. To facilitate selective detection by SDS-PAGE, we fused the precursors with distinct mass tags: LppA^LC^ was tagged with an N-terminal maltose-binding protein (MBP) and a C-terminal thioredoxin A (TrxA), whereas the lasso peptide precursor PbaA^LC^ was flanked by an N-terminal TrxA and a C-terminal chitin-binding domain (CBD). As expected, the RRE domains of LppB and PbaB1 selectively bound their cognate peptides under competing conditions (Fig. 4b and S8).

To investigate the specific mechanism of peptide recognition, we determined the crystal structures of the LppB•LppA^L^ and PbaB1•PbaA^L^ complexes (pdb_00009x8z and pdb_00009×90, 2.0 Å and 2.1 Å, respectively, Supplementary Table 1). Their overall structures are similar to those of previously reported actinobacterial lasso peptide RRE•leader complexes (pdb_00006jx3; pdb_00005v1v; pdb_00005v1u). Both LppB and PbaB1 have winged helix-turn-helix (wHTH) topology and are composed of a β sheet formed by three antiparallel β strands (β2–β4) and three α helices (α1–α3), complemented by two short antiparallel β strands (β1 and β5) that lock the N and C termini together (Supplementary Fig. 9).

PbaA^L^ binds to PbaB1 in an extended β conformation essentially in the same manner as observed in the B1•leader complexes of lasso peptides from different organisms reported previously^25,43^. Hydrogen bonds between the backbones of PbaA^L^ K-18 and PbaB1 D68 and E70 residues anchor the N-terminal portion of the leader (Fig. 4c and S10a). The WxxPxL motif of PbaA^L^, a relatively minor variation of the [Y/W]xxPx[L/V/I/F/A] motif with the first residue being tryptophan, is located in a deep hydrophobic cleft between β4 and α1-α3 of PbaB1. The cleft is divided into two pockets by the conserved F83 residue (Fig. 4c). PbaA^L^ W-15 is surrounded by PbaB1 I44, L65, Y69, and V71 and is held in place by hydrogen bonds with the side chains of PbaB1 Q79 and E75 (Fig. 4c and S10b), which further fix the tryptophan residue within the first hydrophobic pocket. PbaA^L^ P-12 is surrounded by PbaB1 I44, I48, and F83 (Fig. 4c and S10c). The conserved PbaA^L^ L-10 is embedded in the second hydrophobic pocket formed by PbaB1 Y39, L41, F83, L87, and L92 (Fig. 4C and S10D). PbaA^L^ E-9, V-8, and L-7 align with PbaB1 Y38-N40, extending the antiparallel β sheet of PbaB1 (Fig. 4c).

The interaction between LppB and LppA^L^ also follows the same “knobs-into-holes” logic described for lasso peptides^25,43^. The N-terminal part of LppA^L^ is fixed by hydrogen bonds between the LppA^L^ K-18, Y-16, and LppB E61 backbones (Fig. 4d and S10e). The hydrophobic residues V35, C39, I56, Y60, V63, and V67 in the first pocket of LppB tightly hold the conserved LppA^L^ Y-16 (Fig. 4d and S10e). The position of LppA^L^ Y-16 is further fixed by a hydrogen bond with LppB D70, as was seen in the actinobacterial lasso peptide RRE leader complex^43^. LppA^L^ P-13 is sandwiched by LppB V35 and F74. Hydrogen bonds between the peptide backbone and the side chain of LppB N33 fix the kinked conformation around LppA^L^ P-13 (Fig. 4d and S10g). The second hydrophobic pocket of LppB, flanked by T30, L32, F74, M78, and L83, anchors LppA^L^ V˗11 (Fig. 4d and S10h). The central part of the leader (residues Q-7 to M-12) forms an extended antiparallel β sheet with residues Y27 to N33 of LppB. However, LppA^L^ bends sharply at L-10 (Supplementary Fig. 11), forming a classic antiparallel β bulge^44^. The C-terminal part of the leader does not make obvious contact with LppB, suggesting that this portion of the consensus sequence may be required for interaction with the modifying enzymes.

Mutagenesis studies of the bacilli and actinobacterial lasso peptide precursors unambiguously showed the importance of the Y/WxxP motif for the binding to RRE^21,25,43^. To investigate the role of conserved residues in LppA^L^ on its interaction with LppB, we conducted a mutational analysis. Consistent with the crystal structure, Y-16A and P-13A substitutions in LppA^LC^ reduced the efficiency of complex formation in vivo, as evidenced by reduced levels of soluble LppB (Supplementary Fig. 12) and decreased recovery of soluble LppB•LppA^LC^ complexes in pull-down assays (Fig. 4e). The V-11A and L-10A substitutions had no noticeable effect on LppA^LC^ binding affinity to LppB (Fig. 4e). Thus, similar to the RRE•lasso peptide precursor complexes^25,43^, the conserved leader motif YxxP is essential for the efficient formation of the LppB•LppA^LC^ complex.

Together, our data demonstrate that, despite substantial structural similarities and shared modes of peptide binding, the Bacilli RRE domains have evolved to distinguish between the LPP and lasso leader peptides.

### WxxP and YxxP motifs in leader peptides dictate RRE binding specificity

**Fig. 5:**
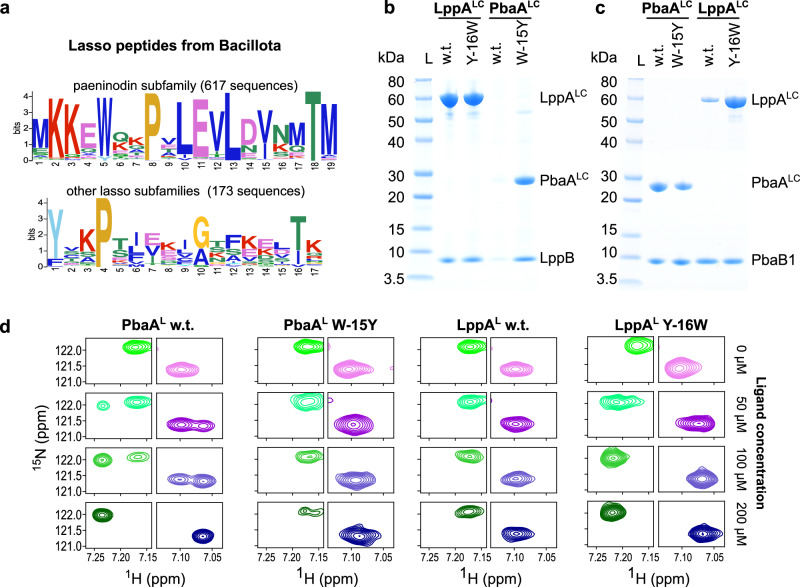
Leader peptide residues direct cognate peptide recognition by LppB and PbaB1. **a** Logos of leader peptide motifs in paeninodin (top) and other lasso peptide subfamilies (bottom) from Bacillota identified using MEME^62^. The datasets were obtained from Kretsch et al.^45^. **b** Purification of strep-tagged LppB co-expressed with wild-type or mutant LppA^LC^ and PbaA^LC^ peptides. **c** Purification of strep-tagged PbaB1 co-expressed with wild-type or mutant LppA^LC^ and PbaA^LC^ peptides. All pull-down experiments in B and C were performed independently three times with similar results. **d** Chemical shift perturbations of characteristic PbaB1 resonances upon binding to the indicated precursor peptide variants. For each peptide variant, the left and right panels correspond to the two individual characteristic resonances, respectively. For a detailed description, see the main text. Source data are provided as a Source Data file.

It has previously been noted that, unlike lasso peptides from other phyla, most Bacillota lasso peptides contain a WxxP rather than a YxxP motif in the leader sequences^25^. However, the significance of this difference has remained unclear. To confirm the prevalence of the WxxP, we searched for conserved motifs in the leader sequences of Bacillota lasso peptides using the comprehensive dataset published by Kretsch et al.^45^ Consistent with the observations reported by Tietz et al.^31^, tryptophan at the first position of the motif is nearly invariant among paeninodin-like lasso peptide precursors. In contrast, and similar to LPP, non-paeninodin lasso precursors from Bacillota predominantly harbor the YxxP motif (Fig. 5a). Given that LPP and paeninodin BGCs are frequently found within the same genomes, we hypothesized that the conserved tyrosine or tryptophan residue in their leader peptides contributes to RRE-mediated discrimination against non-cognate precursors.

To explore this possibility, we generated LppA^LC^ Y-16W and PbaA^LC^ W-15Y variants and examined their interactions with LppB and PbaB1. Pull-down assay results revealed that LppB binds to both wild-type LppA^LC^ and its Y-16W variant with similar efficiency, suggesting that tryptophan is readily tolerated in LPP leaders (Fig. 5b). Since the LppB•LppA^LC^ Y-16W complex formed more efficiently than the LppB•LppA^LC^ Y-16A one (Fig. 4e), this tryptophan residue presumably interacts with the ligand-binding hydrophobic pocket of LppB. At the same time, LppB did not bind wild-type PbaA^LC^ even under non-competing conditions and therefore remained insoluble (Supplementary Fig. 13). This apparent discrepancy in the binding behavior of LppA^LC^ Y-16W and PbaA^LC^ can be explained by the contacts between LppB and the C-terminal region of the cognate leader peptide, which stabilize the complex. However, replacing W-15 with tyrosine turned PbaA^LC^ into a suitable ligand for LppB (Fig. 5b). Thus, the presence of a tyrosine residue in the RRE binding motif is sufficient for binding a non-cognate peptide, emphasizing that the presence of Y rather than W in the leader peptide is the main recognition factor for LppB.

**Fig. 6:**
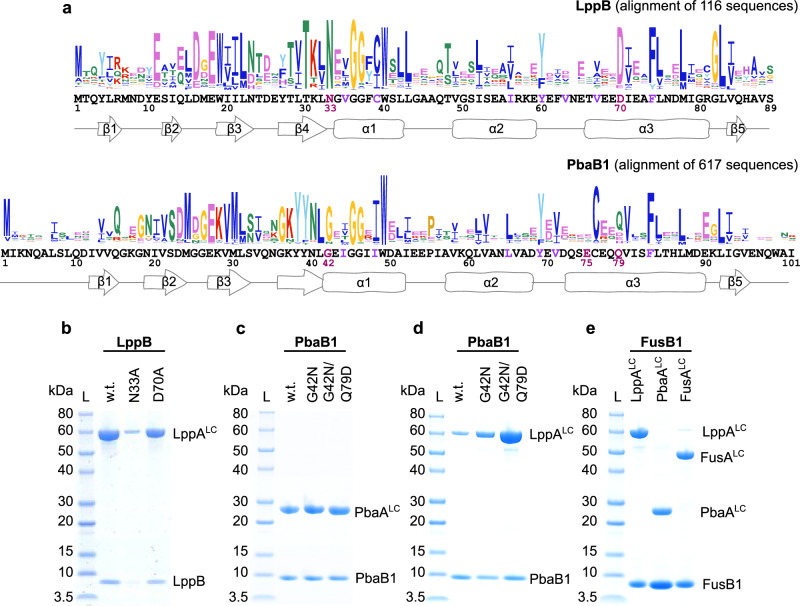
Conserved residues in discrete RRE domains from Bacillota define exclusion of non-cognate leader peptides. **a** Sequences of LppB (top) and PbaB1 (bottom) with residue conservation shown as WebLogos^63,64^ and secondary structure elements depicted as arrows (β strands) and rectangles (α helices). The dataset for paeninodin-like lasso peptide RREs alignment was obtained from Kretsch et al.^45^ Amino acid residues forming hydrophobic interactions with the YxxP and WxxP motifs are highlighted in magenta. Residues analyzed by mutagenesis are shown in red. **b** Purification of LppA^LC^ with strep-tagged wild-type and mutant LppB. **c** Purification of PbaA^LC^ with strep-tagged wild-type and mutant PbaB1 **d** Purification of LppA^LC^ in complex with strep-tagged wild-type and mutant PbaB1. **e** FusB1, a discrete RRE from the fuscanodin lasso peptide pathway, binds cognate FusA^LC^ as well as non-cognate PbaA^LC^ and LppA^LC^ peptides in the pull-down assay. LppA^LC^ was expressed as an MBP-LppA^LC^-TrxA fusion (MW 61.5 kDa), PbaA^LC^ as a TrxA-PbaA^LC^-CBD fusion (MW 24.6 kDa), and FusA^LC^ as an MBP-FusA^LC^ fusion (MW 45.7 kDa). L, protein MW ladder. All pull-down experiments were performed independently three times with similar results. Source data are provided as a Source Data file.

The binding behavior of PbaB1 toward the precursor peptide variants mirrored that observed for LppB. PbaB1 accepted the PbaA^LC^ W-15Y mutant, though with slightly lower efficiency (Fig. 5c). Although being less selective against the non-cognate peptide than LppB, PbaB1 bound LppA^LC^ Y-16W with an apparent affinity similar to that of its cognate precursor, which underscores the preference for tryptophan-containing peptides (Fig. 5c). Though PbaB1 E75 and Q79 form hydrogen bonds with PbaA^LC^ W-15 in the crystal structure, they are not strongly conserved (Fig. 6a), suggesting that the dedicated hydrophobic pocket of paeninodin RREs is generally sufficient for complex formation. Thus, the identity of the first residue in the Y/W motif is critical for the binding to canonical discrete RREs with specialized recognition pockets. However, additional anchoring mechanisms for precursor peptides likely exist, as a substitution at this position can be tolerated when the rest of the peptide remains wild type.

To validate the results of our pull-down assay, we attempted to measure the dissociation constant (K_D_) for LppB•LppA^L^ and PbaB1•PbaA^L^ complexes. However, similar to some lasso peptide RREs^46^, LppB remained insoluble in the absence of the cognate precursor peptide, even after refolding, thus precluding quantitative analysis. The PbaB1 proved soluble without a peptide, and its CD spectrum indicated the presence of β strands and α helices (Supplementary Fig. 14), in agreement with our crystal structure. However, when we recorded the ^1^H, ^15^N HSQC NMR spectrum of isotopically labeled PbaB1, we observed only approximately half of the expected amide correlation peaks (Supplementary Fig. 15). This might indicate internal protein flexibility, whereby exchange between different conformations leads to line broadening in its more flexible regions. When we saturated PbaB1 with the PbaA peptide, the majority of the expected amid correlation peaks were recovered (Supplementary Fig. 15), suggesting that the marginally stable PbaB1 folds into a stable domain upon complexing its cognate peptide.

To quantify the affinity of PbaB1 for individual peptides, the protein was titrated with increasing concentrations of each peptide, and 2D spectra were recorded at each titration point. Depending on the affinity constant and exchange kinetics, we observed distinct spectral behaviors. The titration with the cognate PbaA^L^ peptide illustrates the first scenario, in which both the free and complexed PbaB1 coexist in solution as two separate entities that do not exchange on the time scale imposed by the chemical shift difference between equivalent peaks (Fig. 5d and S15). This behavior is characteristic of complexes with K_D_ values below 1 µM. Although NMR cannot be more precise in this case, it confirms that this complex is an example of tight binding^47^. In contrast, the non-cognate LppA^L^ peptide induced little change in the PbaB1 spectrum until the peptide reached millimolar concentration. Both PbaB1 conformers, free and complexed with the LppA^L^, are thus in fast exchange, resulting in an average peak whose chemical shift reflects the fraction of complexes formed (Supplementary Fig. 16). The fit of the chemical shift perturbations as a function of peptide concentration yielded a weak K_D_ ~ 1.5 mM (Supplementary Fig. 16). For the LppA Y-16W and PbaA W-15Y peptides, the behavior was intermediate. At substoichiometric amounts of the LppA Y-16W peptide relative to PbaB1, some peak doubling was observed, but the peaks also broadened (Fig. 5d). At higher concentrations of LppA Y-16W, only the bound conformation was detected, and we recovered the initial line width. For the PbaA W-15Y peptide, we observed substantial line broadening of the left peak at higher peptide concentration (Fig. 5d), while the right peak only slightly broadened but shifted. Approximation of the chemical shift perturbation trajectories enabled estimation of K_D_ values of approximately 10 µM for LppA Y-16W and 40 µM for PbaA W-15Y, respectively.

Together, our pull-down assay and NMR analysis of PbaB1•leader peptide complex formation consistently indicate that tryptophan or tyrosine residues in leader peptides constitute the primary determinants by which LPP and paeninodin-like lasso RREs achieve cognate peptide selection. However, the kinetic data also highlight the limited sensitivity of the pull-down approach in resolving affinity differences for complexes with K_D_ values in the low tens of micromolar or lower.

### RREs evolved to select cognate precursor peptides

To gain deeper insight into the mechanism of the Y/WxxP-peptide selection, we closely examined the structures of the RRE•leader peptide complexes. We first assessed conservation in both RREs of amino acid residues that interact directly with the YxxP and WxxP motifs. In both RREs, most contacts are hydrophobic, and importantly, the residues mediating these interactions are either identical or evolutionarily permissive in the two RRE domains (Fig. 6a), suggesting that they are unlikely to account for leader peptide selectivity.

However, some notable differences are apparent. In the structure of the LppB•LppA^L^ complex, the hydrogen bond between the highly conserved LppB D70 (Fig. 6a) and LppA^L^ Y-16 stabilizes the complex (Fig. 4d and S10f). In the same structure, the kinked backbone conformation at LppA^L^ P-13 is stabilized by hydrogen bonds with the side chain of N33 (Fig. 4d and S10g). Both D70 and N33 residues are highly conserved among LPP RREs (Fig. 6a) and in RREs of many PQQ and lasso peptides across diverse phyla (Supplementary Fig. 17), suggesting an evolutionarily conserved mechanism for efficient capture of their precursors. Experimental data confirm the importance of these hydrogen bonds for high-affinity binding: the LppB D70A mutations reduced the efficiency of LppB•LppA^LC^ complex formation, while the N33A substitution almost abolished complex assembly (Fig. 6b and S18).

Paeninodin-like lasso peptide RREs have a conserved glycine residue at the position corresponding to N33 of LppB (Fig. 6a). We propose that this asparagine-to-glycine substitution contributes to discrimination against non-cognate precursors. We tested the effect of substituting Gly42 with Asn in PbaB1 on substrate discrimination. The mutation did not visibly affect the binding of PbaA^LC^ (Fig. 6c) but enhanced non-specific interaction of PbaB1 with wild-type LppA^LC^ (Fig. 6d). To further test whether the PbaB1 promiscuity could be increased, we introduced a second mutation that mimics the tyrosine-binding D70 in LppB. As expected, the G42N/Q79D substitutions in PbaB1 did not alter recognition of its cognate peptide but dramatically enhanced binding of the tyrosine-containing LppA^LC^, thus confirming the role of these residues in the cognate peptide selection.

Since the YxxPx[L/V/I/F/A] motif predominates in lasso peptide leaders^25^, we next asked whether RREs from outside Bacillota could discriminate against LPP precursors. To this end, we used a well-characterized FusB1 encoded in the actinobacterial fusilassin/fuscanodin lasso peptide BGC^22^ to test its potential promiscuity. In contrast to LppB and PbaB1, FusB1 bound not only its cognate precursor FusA^LC^ but also the non-cognate PbaA^LC^ and LppA^LC^ precursors with similar efficiency (Fig. 6e), highlighting the remarkable promiscuity of lasso peptide RREs outside the Bacillota phylum.

Collectively, our findings suggest that LPP and paeninodin-like lasso RREs employ evolutionarily conserved mechanisms for accurate precursor peptide selection.

### LppB presents the LppA^LC^ leader cleavage site to the SpI-like leader peptidase LppS

**Fig. 7:**
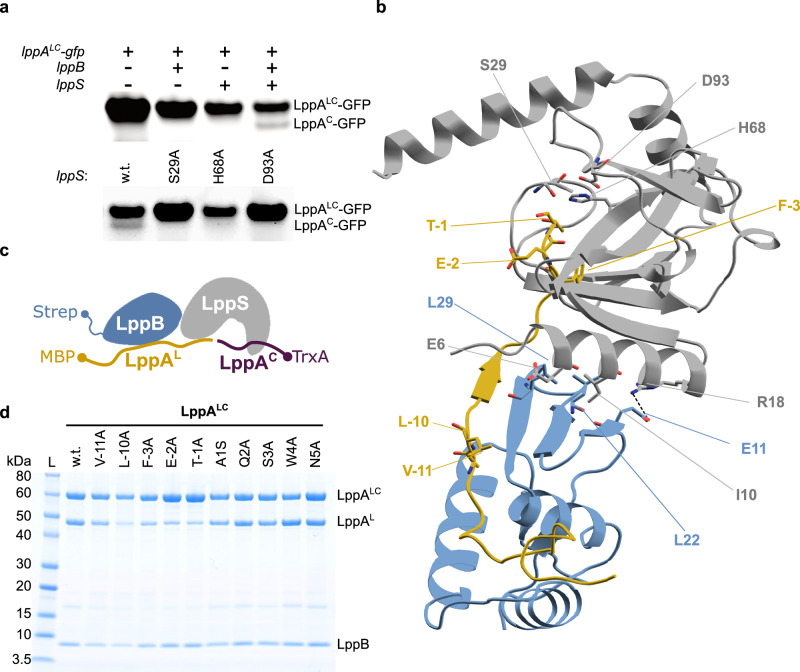
LppB and LppS cooperate for the LppA^LC^ leader peptide cleavage. **a** In vitro translation/cleavage assay. LppA^LC^ fused at the C-terminus with GFP was co-translated with LppB, LppS, or both (top). LppA^LC^-GFP was co-translated with LppB and either wild-type LppS or its catalytic mutants (bottom). LppA^LC^, LppA^LC^ precursor peptide fused to GFP, LppA^C^, the core part of the LPP fused to GFP. **b** Ribbon diagram of the LppS•LppB•LppA^L^ complex modeled with AlphaFold3. Residues forming intermolecular contacts are shown as sticks. **c** Schematic of the in vivo cleavage and pull-down assay. **d** In vivo cleavage and pull-down analysis of LppB in complex with wild-type or mutant LppA^LC^. L, protein MW ladder, LppA^LC^, MBP-LppA^LC^-TrxA fusion (MW 61.5 kDa), LppA^L^, LppA^L^ fused to MBP (MW 45.1 kDa). All pull-down experiments in (**a**) and (**d**) were performed independently three times with similar results. Source data are provided as a Source Data file.

The primary role of the RRE domain in lasso peptide biosynthesis is to present the precursor to cysteine peptidase B2 for cleavage^21,32,48^. Aided by structure prediction using AlphaFold, mutational analysis identified the binding interface between RRE and B2 peptidase, which was found to be conserved in Actinobacteria and Bacillota^45,49^. By analogy, we propose that LppB in the LPP system could also be essential for LppS-mediated cleavage of the LppA^LC^ leader peptide. To investigate this possibility, we employed an in vitro translation/cleavage assay^49^. We expressed the LppA^LC^ precursor C-terminally fused to green fluorescent protein (GFP), either alone or in combination with LppB and/or LppS. Although the LppK kinase was not included in the system, we anticipated detectable proteolysis of unmodified peptide^50^. Indeed, while LppS alone failed to process the LppA^LC^-GFP fusion, co-expression of LppS with LppB resulted in the appearance of a lower-molecular-weight band corresponding to LppA^C^-GFP lacking the leader sequence (Fig. 7a, upper panel). No cleavage was observed when catalytically inactivated variants of LppS (S29A, H68A, or D93A) were tested (Fig. 7a, lower panel).

Due to the poor solubility of LppS we were unable to co-crystallize the LppS•LppB•LppA^LC^ complex and therefore relied on AI-based structure prediction. AlphaFold3^51^ predicted with high confidence (average pLDDT score = 89.8) that LppS interacts with both LppA^L^ and LppB (Fig. 7b and S19). In contrast to the lasso peptide B2•B1•A complex, where B1 engages the peptidase via a β sandwich interaction^45,49^, the extended intermolecular β sheet of LppB and LppA^L^ forms hydrophobic contacts with the N-terminal α1 helix of LppS (Supplementary Fig. 20a). Two “locks” of hydrogen bonds on both sides of the interface - between LppB E11 and LppS K17/R18, and between LppB K31 and LppS E6 – likely stabilize the complex (Supplementary Fig. 20b, c).

Although the conformation of LppB and LppA^L^ in the predicted complex closely matched the crystal structure (RMSD = 2.02 Å) supporting the accuracy of the model, we sought to validate this prediction experimentally. To this end, we performed an in vivo cleavage/pull-down assay in which the wild-type MBP-LppA^LC^-TrxA fusion was co-expressed with LppS and Strep-tagged LppB. Both intact and cleaved forms of LppA^LC^ co-purified with LppB (Fig. 7c). Consistent with the structural model, in vivo cleavage/pull-down assay revealed that the mutagenesis of LppS E6 and I10, and LppB E11, L22, and L29 markedly reduced cleavage efficiency (Supplementary Fig. 21). The LppS R18 mutation did not significantly affect LppA^LC^ cleavage, likely due to the remaining interactions between other residues. Notably, in contrast to LppB (Fig. 6a), the LppS residues forming the intermolecular contacts are moderately conserved (Supplementary Fig. 22), suggesting that this interaction serves a secondary function in the assembly of the tripartite complex.

According to the AlphaFold3 model, the C-terminal residues of the LppA^L^ form multiple hydrogen bonds with LppS (Supplementary Fig. 20d), LppA^L^ F-3 is inserted into a hydrophobic cleft near the highly conserved LppS residue F42 (Supplementary Figs. 20e, 22), while the terminal T-1 residue of the leader is located near the catalytic center of LppS (Fig. 7b and S20f). To validate the relevance of this prediction, we analyzed the effect of mutations in conserved LppA^LC^ residues (Fig. 2d) on leader cleavage efficiency. As shown in Fig. 7d, substitutions in the core region (LppA^C^ A1–N5) did not impair processing. In contrast, mutations of the three C-terminal leader residues (LppA^С^ F-3, E-2, and T-1) reduced cleavage efficiency, in agreement with the structural model. Similarly, substitution of the conserved LppA^L^ L-10 residues markedly diminished proteolysis, indicating its essential role for proper presentation of the precursor to the peptidase.

Collectively, our data support that LPP precursor peptides and RRE co-evolved to engage an SpI-like serine peptidase for leader cleavage.

## Discussion

In this work, we identified a family of RiPPs whose biosynthesis proceeds through an intermediate bearing a polyphosphate post-translational modification. The LPP biosynthesis involves the SpI-like leader peptidase, an addition to the list of known RiPP-processing enzymes^34^. Our genetic data indicate that the unmodified LPP precursor is a poor substrate for leader cleavage, highlighting the tight coordination of modification and processing steps in LPP biosynthesis. Similar to canonical cysteine leader peptidases in the lasso peptide systems^21^, LppS requires RRE-assisted presentation of the precursor for efficient cleavage.

The nearly identical system of precursor peptide recruitment further underscores the close link between the LPP and lasso peptide RiPPs. BGCs of both RiPP families encode discrete RRE domains and precursor peptides that share consensus [Y/W]xxP RRE-binding motifs in their leaders. However, our data show that, despite the high similarity in the binding motifs, the biosynthetic machineries effectively discriminate between cognate and non-cognate precursors, ensuring the integrity of the pathways and proper allocation of resources. Our structural and mutational analyses revealed that the choice of tyrosine *vs*. tryptophan within the conserved leader motif is a key determinant of RRE specificity. In LPPs, high-affinity precursor binding is mediated by fixing tyrosine-containing motifs via a hydrogen bond, a mechanism identical to that utilized in most lasso peptide systems^25,43^. In contrast, paeninodin lasso peptide RREs have a hydrophobic pocket that favors tryptophan-containing precursors and reduces the affinity with non-canonical peptides by losing the hydrogen bonds that help to fix the conserved proline residue. Unlike LPP and paeninodins, which are restricted to the Bacillota, lasso peptide RREs from other phyla, such as the actinobacterial FusB1^22^, retained relaxed specificity for the precursor peptides. The example of LPP and lasso peptide co-evolution highlights the versatility of the RRE fold, which can simultaneously tolerate closely related motifs and maintain robust discrimination.

**Fig. 8:**
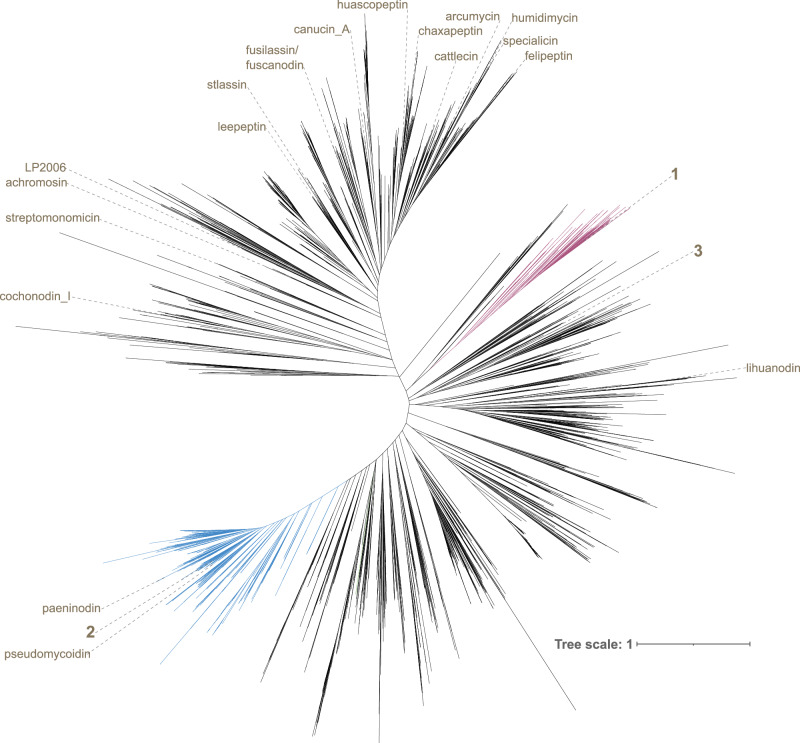
Approximate maximum-likelihood tree of discrete RRE domains from lasso peptide and LPP RiPP BGCs. B1 proteins encoded within BGCs responsible for the biosynthesis of known lasso peptides are indicated on the tree. The paeninodin family clade is shown in blue, and the LPP clade is highlighted in mauve. 1 – LppB; 2 – PbaB1; 3 – BsfB1^23^.

The phylogenetic analysis of available RRE-containing domains suggests that paeninodins and LPP belong to distinct monophyletic groups (Fig. 8). Both groups seem to have emerged independently from a wider class of lasso peptide RREs, but they have converged on similar modification pathways with other phosphate-modified lasso peptides^23^ (Supplementary Fig. 23). Except for the macrolactam cyclase, the LPP core biosynthetic genes are functionally identical to those of the lasso peptides from the paeninodin^32,33^ and Bsf subfamilies^23^, suggesting that structurally, LPPs are linear analogs of phosphorylated lasso peptides.

Mapping this set of RREs onto 9173 completely sequenced Bacilli genomes reveals complex and extremely patchy phyletic patterns for these of RiPPs. Paeninodins and LPPs are present in overlapping sets of 344 and 80 genomes, respectively, but their evolutionary trajectories involve at least 102 and 61 gain and loss events, respectively. Such a dynamic history suggests extensive selection-driven horizontal gene transfer, typical for genes involved in all sorts of biological conflicts^52,53^. On the flip side, this dynamism effectively erases the history of the systems’ origins and confounds the co-occurrence analysis by imposing the strong ecological pressure against possible intra-genomic constraints, leaving many questions about interactions between these systems open.

The biological roles of paeninodins and LPPs remain unknown. Both RiPPs often co-localize with capsular polysaccharide loci in the genomes of Bacilli. Although it is tempting to speculate that LPPs and paeninodins contribute to biofilm formation, this hypothesis has yet to be tested in future studies.

## Methods

### Bacterial strains and growth conditions

*Paenibacillus alginolyticus* DSM5050 strain^54^ was purchased from the German Collection of Microorganisms and Cell Cultures (DSMZ). *Bacillus subtilis* 168 was from the laboratory stock. *E. coli* DH5α (Toyobo) was used for the cloning procedures, and *E. coli* BL21(DE3) (Agilent) was used for the protein expression. All bacteria were routinely grown on LB media at 37 °C.

### Molecular cloning

All oligonucleotide primers and synthetic DNA fragments used in this study are listed in Supplementary Data 3 and Supplementary Table 2, respectively. The *fusB1* and *fusA*^*LC*^ synthetic genes were described previously by Alfi et al.^49^ *P. alginolyticus* DSM5050 genomic DNA was used as a PCR template, unless stated otherwise. Vectors generated in this study are available from the corresponding authors upon request.

### Bioinformatics

A dataset of 240 sequences of proteins, homologous to *P. alginolyticus* DSM5050 SpI protease (NCBI accession number WP_029196374.1), was collected from the NCBI protein database^55^ in February 2019 using BLASTP search^56^ with an E-value cutoff of 0.001. A sequence similarity network (SSN) was constructed using the EFI-EST web service^57^ (https://efi.igb.illinois.edu/efi-est/). Edges of the network corresponding to E-value higher than 10e-23 were removed, and the resulting network was visualized using Cytoscape^58^.

Analysis of 12 kb-genomic regions upstream and downstream to SpI homologs was performed using the RODEO web tool^31^ (https://webtool.ripp.rodeo/) with Pfam^59^ and TIGRFAMs^60^ databases. Short (less than 3 kb) and truncated contigs were manually removed, resulting in a final dataset of 199 sequences. Co-occurrence of protein domains was calculated using a Python script (available at https://github.com/bikdm12/lpp) and visualized in Cytoscape^58^. Several proteins involved in specialized biochemical reactions (such as macrolactam synthases) lacked specific models in protein domain databases, causing multiple related models to match the same protein with similar E-values and yield inconsistent annotations. To correct this, we manually grouped related models and summed their counts. The grouping is available in Supplementary Data 2.

For the initial identification of putative precursor peptide sequences in *lpp*-like BGCs, all open reading frames (ORFs) meeting the following criteria were collected: located within the BGC core or in the region up to 2 kb upstream of the putative BGC core and encoded in the same direction as the BGC, no overlap with annotated genes by more than five codons from the N-terminus, length in the range of 30–70 codons, and an ATG start codon preceded by a strong Shine-Dalgarno sequence. The Clustal Omega^61^ multiple sequence alignment of the collected sequences revealed the conserved Px_9_Fx[T/A] motif. This motif was used for scanning of ORFs retrieved after a second round of searches with less stringent criteria: location within the BGC or in the region 5 kb upstream or downstream of it, no restrictions on ORF length or orientation, having a start codon ATG, GTG, or TTG. Precursor sequences of lasso peptides were obtained from the previous comprehensive study by Kretsch et al.^45^ Motifs were identified using the MEME web tool^62^ (https://meme-suite.org/).

To visualize conserved amino acid residues in LppB and LppS, their sequences were aligned with the corresponding homologs from the SSN cluster 1 using Clustal Omega (https://www.ebi.ac.uk/jdispatcher/msa/clustalo). For the PbaB1 homologs, the RRE domains encoded in paeninodin-like BGCs from the dataset previously published by Kretsch et al.^45^ were used. Columns of the alignments that correspond to the gaps in the target protein sequences were manually removed before logos construction with the WebLogo web tool^63,64^ (https://weblogo.berkeley.edu/logo.cgi).

Sequences of discrete RRE domains from LPP and lasso peptide BGCs, collected in this study and from the comprehensive dataset of Kretsch et al.^45^, respectively, were aligned using MUSCLE5^65^. An approximate maximum likelihood tree was reconstructed using FastTree^66^ with gamma-distributed site rates and WAG evolutionary model and visualized using iTOL^67^.

Completely sequenced bacterial genomes were downloaded from the NCBI FTP site in November 2023; their approximate phylogeny was reconstructed from concatenated alignments of 54 nearly universal bacterial COGs^68^. RRE domains were mapped to the proteins, encoded in these genomes, using MMSEQS2^69^. Equal weight maximum parsimony reconstruction for two classes of RRE domains (paeninodins and LPPs) within the 9173 genomes of the Bacilli clade was obtained using the Fitch parsimony algorithm^70^. All supporting data are available on Zenodo Repository (10.5281/zenodo.18877617).

### Protein structure prediction

AlphaFold3^51^ was run with default parameters to generate five model structures of the LppS•LppB•LppA^L^ complex. The model with the best pLDDT value was selected for experimental validation. The PbaB1•PbaA^L^ complex structure was predicted by AlphaFold2 in multimeric mode^71,72^ with default parameters. The model with the highest pLDDT score was further used for the molecular replacement to solve the complex crystal structure.

### RT-qPCR

The expression levels of the *pbaB1* and *lppB* genes were measured by quantitative reverse transcription-PCR using primers listed in Supplementary Data 3, corresponding to the genes encoding PbaB1 (WP_029196370.1) and LppB (WP_029196375.1) proteins, and compared with the expression levels of the housekeeping genes encoding for gyrase subunit A GyrA (WP_029196169.1) and polysaccharide biosynthesis tyrosine autokinase (WP_029196380.1). RNA samples were then prepared using the Column RNA Isolation Kit (Biolabmix) according to the manufacturer’s protocol, treated with DNase I, RNase-free (Thermo Scientific), and purified from the reaction mixture using the Column RNA Isolation Kit (Biolabmix). RNA quality was assessed using a NanoDrop 2000 spectrophotometer (Thermo Scientific), and the RNA was then reverse-transcribed into cDNA using the MMLV RT kit (Evrogen). The cDNA was subsequently subjected to a qPCR reaction mixture, Maxima SYBR Green/ROX qPCR Master Mix 2X (Thermo Scientific). Absolute quantification (standard curve method)^73^ was used for data processing. P-values were calculated using two-sided Welch’s t-tests (see Source Data file).

### Heterologous expression of the *lpp* BGC variants

The *lpp* biosynthetic gene cluster and its *lppA*^*LC*^*-D* fragment lacking the putative NTP-transferase gene were PCR amplified and inserted into a pHT01 vector (MoBiTec, cat. no. PBS001) between KpnI and SalI restriction sites. Deletion of *lppK, lppB*, and *lppS* was introduced to the pHT01-*lpp* plasmid using the InFusion cloning kit (Takara). DNA fragments with the deletion of the corresponding genes were obtained by overlap-extension PCR^74^. The expression of *lpp* BGC variants was carried out in the *B. subtilis rok::erm* cells to relieve transcription silencing^42^. The host strain was constructed by disrupting the coding sequence of *rok* in *B. subtilis* 168 using an integrative pSC plasmid and a procedure described in Pomerantsev et al.^75^ Heterologous expression of the *lpp* BGC variants in the *B. subtilis rok::erm* cells was conducted in 1/2 Luria-Bertani (LB) (Tryptone 5 g L^−1^, yeast extract 2.5 g L^−1^, NaCl 2.5 g L^−1^, agar 15 g L^−1^) supplemented with 10 µg L^−1^ of chloramphenicol and 0.1 mM isopropyl-β-D-thiogalactopyranoside (IPTG) for induction. Cells were incubated at 30 °C for 16 h before the MALDI-TOF MS analysis.

MALDI-TOF spectra were recorded using the UltrafleXtreme II MALDI-TOF-TOF (Bruker), equipped with a neodymium laser (355 nm). Cells were mixed in a 1:2 ratio with the matrix solution (40 mg/mL 2,5-dihydroxybenzoic acid (Sigma), 0.5% trifluoroacetic acid (TFA) in 30 % acetonitrile) and placed on a steel target plate. In the mass spectra recorded in reflector (measurement accuracy within 0.1 Da) and linear mode (measurement accuracy was within 0.5 Da), monoisotopic and average mass/charge ratio (*m/z*) values were labeled, respectively. For the fragmentation spectra LIFT mode was used, the accuracy for the detected product ions within the 1 Da range. FlexAnalysis 3.2 software (Bruker) was used for mass-spectra processing.

To confirm phosphorylation of LppA, cells harvested from plates were subjected to methanol extraction (70% methanol, 16 h incubation, centrifugation at 8,000 × g) in three independent replicas. The cell extracts were collected, evaporated in a centrifugal vacuum concentrator (Genevac EZ-2, SP Scientific), dissolved in 100 μL of 0.1% trifluoroacetic acid (TFA), and purified by reverse-phase chromatography on a Milichrom A-02 system (Econova) using a ProntoSil-120-5-C18 AQ column (Econova) at a flow rate of 0.2 mL/min with UV detection at 210 and 280 nm. The column was pre-equilibrated with 0.1% TFA in 5% aqueous acetonitrile and eluted with a linear gradient of 5–80% aqueous acetonitrile containing 0.1% TFA over 20 min. Fractions containing LppA were collected and evaporated prior to mass spectrometry analysis. The high-resolution spectra were recorded as part of the LC-MS analysis on an Agilent 1200 HPLC equipped with the UV and 6550 iFunnel QTOF LC/MS detectors and Jet Stream Technology ion source (Agilent). The analyzed compounds were loaded on the Poroshell 120 SB˗C18 column (2.7 µ, 2.1 × 100 mm, Agilent) at 40 °C using a linear (0 to 80%) gradient of acetonitrile in 5 mM ammonium acetate buffer (pH 5.2) at a 0.2 mL min^−1^ flow rate. The electrospray source was set to positive ion mode at 4 kV, 290 K. Data were analyzed using MassHunter Qualitative Analysis 10.00 software (Agilent).

### Identification of the phosphorylation site

To identify the phosphorylation site, products of the *lpp* BGCs were partially purified. *B. subtilis* cells harboring pHT01-*lpp* were grown on LB agar plates supplemented with 0.1 mM IPTG. Cells were harvested and extracted with 70% methanol at room temperature for 16 h. The extract was collected, dried, and fractionated by reverse-phase chromatography. HPLC was performed using a ProntoSil 120-5-C18 AQ column (Econova) with a 5-80% linear gradient of acetonitrile in 0.1% trifluoroacetic acid at a flow rate of 0.2 mL min^−1^ and UV detection at 210 and 280 nm. HPLC fractions containing phosphorylated and unphosphorylated LppA^C^ peptides were dried and re-dissolved in water. Dephosphorylation was carried out using calf intestinal alkaline phosphatase (CIAP) at 37 °C for 90 min. Peptide digestion with LysC protease was performed at 37 °C for 30 min.

The raw MALDI MS spectra were deposited to the ProteomeXchange consortium via the JPOST repository^76^ with the dataset identifiers PXD070677 for ProteomeXchange and JPST004185 for jPOST.

### In vitro LppA^LC^ cleavage assay

The DNA templates used for the cell-free transcription-translation were PCR-amplified from *P. alginoliticus* genomic DNA (*lppB* and *lppS*) or purchased as a synthetic DNA fragment (*lppA*^*LC*^*˗sfGFP*). Mutations in the *lppS* sequence were introduced by overlap-extension PCR^74^.

The cell-free protein synthesis was performed using the PUREfrex 2.0 system (GeneFrontier) as described in Alfi et al.^49^ In brief., 1.25 μL of solution I, 0.125 μL of solution II, and 0.25 μL of solution III from the PUREfrex 2.0 kit were mixed with 0.125 μL RNase inhibitor (Nacalai Tesque), 0.1 μL each of PCR templates encoding LppA–sfGFP, LppB, and LppS, and 0.45 μL H₂O. For reactions lacking LppB and/or LppS, the corresponding PCR templates were replaced with H₂O. Samples were incubated for 6 h at 37 °C to allow protein expression and peptidase cleavage.

After incubation, samples were mixed with 2.5 μL H₂O and 2.5 μL 4× LDS sample buffer (Invitrogen). Then, 2.5 μL of the mixture was loaded onto SDS-PAGE gels (NuPAGE 4–12% Bis-Tris gel, Invitrogen) without heating to avoid denaturation of sfGFP. Gels were run at 100 V for 60 min and analyzed using an Amersham Typhoon scanner (GE Healthcare).

### Pull-down assays

Coding sequences of LppB, PbaB1, and FusB1 were 5’-terminally extended with the strep-tag coding sequence using PCR and inserted into the pET-Duet1 plasmid (Novagen, cat. no. 71146) by the InFusion method to create pET-*lppB*, pET-*pbaB1*, and pET-*fusB1*, respectively.

The competition-binding assay was performed in BL21(DE3) cells harboring pET-*lppB* or pET-*pbaB1* plasmids in combination with pAIR-*lppA*^*LC*^*-pbaA*^*LC*^ vector. The pAIR-*lppA*^*LC*^*-pbaA*^*LC*^ was constructed by introducing *lppA*^*LC*^ between KpnI and SalI restriction sites into the first expression cassette of the pAIR plasmid^43^, thus creating the *mbp-lppA*^*LC*^*-trxA* coding sequence. Then, the CBD-encoding DNA fragment was PCR-amplified using the pTXB1 vector (New England Biolabs, cat. no. N6707S) as a template and combined with the synthetic *trxA_pbaA*^*LC*^ fragment by overlap-extension PCR^74^ to obtain *trxA_pbaA*^*LC*^*_cbd* fusion, which was then inserted into the second expression cassette of pAIR-*lppA*^*LC*^ using the InFusion cloning kit (Takara) to generate pAIR-*lppA*^*LC*^*-pbaA*^*LC*^ vector.

For the non-competitive pull-down assays, *E. coli* BL21(DE3) cells were transformed with combinations of pRSF-*lppA*^*LC*^ and pET-*lppB*, pRSF-*pbaA*^*LC*^ and pET-*pbaB1*, or pRSF-*fusA*^*LC*^ and pET˗*fusB1* vectors. The pRSF-*lppA*^*LC*^ and pRSF-*pbaA*^*LC*^ were obtained by PCR amplification of the *mbp-lppA*^*LC*^*-trxA*^*LC*^ and *trxA-pbaA*^*LC*^*-cbd* DNA fragments from the pAIR-*lppA*^*LC*^*-pbaA*^*LC*^ vector, followed by their insertion into the pRSF-Duet1 vector (Novagen, cat. no. 71341) by the InFusion technique to excise the second expression cassette. pRSF-*fusA*^*LC*^ was obtained by PCR amplification of the FusA^LC^ coding sequence followed by its insertion into the pRSF-*lppA*^*LC*^ vector by the InFusion technique to generate *mbp˗fusA*^*LC*^ fusion.

Coupled phosphorylation and pull-down assay was carried out in E. coli BL21(DE3) cells co-transformed with the pAIR-*lppA*^*LC*^*-lppB* and pET-*lppK* plasmids. The pAIR-*lppA*^*LC*^*-lppB* vector was constructed by inserting the LppB coding sequence into the second expression cassette of the pAIR-*lppA*^*LC*^ plasmid using the InFusion kit, with a forward primer carrying a 5′ Strep-tag sequence. The pET-*lppK* construct was obtained by inserting the LppK coding sequence into the pET-Duet˗1 vector between BamHI and XhoI restriction sites.

In vivo LppA^LC^ cleavage and pull-down assay was performed in *E. coli* BL21(DE3) cells co-transformed with pRSF-*lppA*^*LC*^ and pET-*lppB-lppS* vectors. The latter was constructed by inserting a DNA fragment containing the *LppB* and *LppS* coding sequences into the pET-Duet-1 vector using the InFusion method, with a forward primer carrying a 5′ Strep-tag sequence.

All point mutations in *lppA*^*LC*^, *lppB*, *lppS*, *pbaA*^*LC*^, and *pbaB1* were introduced by overlap-extension PCR^74^ and cloned into the corresponding vectors.

Protein expression was performed in 15 mL of LB liquid media (Nacalai) supplemented with 100 µg mL^−1^ of ampicillin and 30 µg mL^−1^ of kanamycin. 0.1 mM IPTG was added for induction once the cultures reached OD_600_ 0.6. After induction, the cells were grown at 37 °C with vigorous shaking for 2 h, then collected by centrifugation, resuspended in 1 mL of wash buffer WB1 (25 mM Tris–HCl, pH 8.0, 150 mM NaCl, 5 mM EDTA), and lysed by sonication. The lysates, cleared by centrifugation at 20,000 × *g* for 25 min, were combined with 80 μL of Strep-Tactin Superflow Plus resin (Qiagen) pre-equilibrated with WB1 and incubated for 45 min with gentle rotation at 4 °C. The resin was washed with WB1, and the protein complex was eluted with 100 μL of elution buffer EB1 (25 mM Tris–HCl, pH 8.0, 150 mM NaCl, 5 mM EDTA, 2.5 mM desthiobiotin). The fractions were combined with LDS sample buffer (Invitrogen) and analyzed by SDS-PAGE using NuPAGE 4–12% Bis-Tris gels (Invitrogen). Gels were stained with Bullet CBB Stain One (Nacalai).

The identity of the purified proteins, phosphorylation status, and the accuracy of leader peptide cleavage by LppS were confirmed by tryptic peptide fingerprinting combined with mass spectrometry^77^ using Mascot search^78^.

### RRE•leader peptide complex purification

For purification of RRE•leader peptide complexes, the *lppA*^*L*^ and *pbaA*^*L*^ gene fragments encoding the leader peptides were inserted into the first expression cassette of the pAIR vector^43^ using the In-Fusion method to construct pAIR-*lppA*^*L*^ and pAIR-*pbaA*^*L*^ vectors. Subsequently, DNA fragments encoding 6xHis-LppB were inserted into the pAIR-*lppA*^*L*^ vector between the BamHI and XhoI restriction sites to generate the pAIR-*lppA*^*L*^*-lppB* plasmid. Similarly, DNA fragment encoding 6xHis-PbaB1 was inserted into the second expression cassette of pAIR-*pbaA*^*L*^ using In-Fusion cloning to yield the pAIR-*pbaA*^*L*^*-pbaB1* vector.

The overnight culture of *E. coli* BL21(DE3) cells harboring the pAIR-*pbaA*^*L*^*-pbaB1* or pAIR-*lppA*^*L*^*-lppB* plasmids was used to inoculate 2 L of LB medium supplemented with 30 μg mL^˗1^ kanamycin. The culture grew at 37 °C until OD_600_ reached 0.6 before the protein expression was induced with 0.1 mM IPTG. The induced culture was transferred to 18 °C and allowed to grow for 14 h with vigorous shaking. The cells were then collected by centrifugation, resuspended in 80 mL of the wash buffer WB2 (25 mM Tris-HCl, pH 8.0, 500 mM NaCl, 5 mM imidazole, pH 8.0), and lysed by sonication. Cleared lysates, obtained by centrifugation at 20,000 × *g* for 25 min, were applied to the 5 mL HisTrap HP His-tag protein purification column (Cytiva). After washing the column with the WB2, the pure protein sample was eluted with the elution buffer EB2 (500 mM NaCl, 25 mM Tris-HCl, pH 8.0, 300 mM imidazole). The eluted fractions were concentrated with the Amicon Ultra centrifugal filter (Millipore) and applied to a gel filtration column HiLoad Superdex 200 16/600 (Cytiva). The fractions were analyzed by SDS-PAGE using NuPAGE 4–12% Bis-Tris gels (Invitrogen). Fractions, containing the target protein complex, were pulled and concentrated with an Amicon Ultra centrifugal filter (Millipore) for further crystallization. Protein concentrations were measured by NanoDrop One spectrophotometer (Thermo Scientific) at 280 nm.

### Crystallization, data collection, and structure determination

The data collection and refinement statistics are presented in Supplementary Table 1.

For crystallizing LppB•LppA^L^ complex, 0.2 µL of the protein solution (2.5 mg mL^−1^) was combined with the equivalent volume of the commercial reservoir solution (Rigaku), containing 100 mM potassium phosphate buffer, pH 6.2, 20% (w/v) PEG 1000, and 200 mM NaCl. Crystallization was performed by the sitting drop vapor diffusion technique at 4 °C. The X-ray data were obtained at Proton Factory BL5-A. The crystallization solution supplemented with 30% glycerol (v/v) was used for cryoprotection. LppB•LppA^L^ complex belongs to P 2_1_ 2_1_ 2_1_ space group with unit-cell dimensions *a* = 52.90, *b* = 55.60, and *c* = 83.87 and α = β = γ = 90°. Phenix.Xtriage analysis^79^ indicated the presence of translational non-crystallographic symmetry (tNCS), which likely contributed to the relatively high R values observed during refinement. The structure was solved by molecular replacement with the TbiB1•TbiAα^L^ structure (pdb_00005v1v) as a search model. The final structure was refined at 2.1 Å to R_work_ and R_free_ values of 0.20 and 0.25, respectively.

To obtain the crystals of PbaB1•PbaA^L^ complex, 0.2 µL of the protein solution (3.3 mg mL^˗1^) was combined with an equal volume of the reservoir solution (Rigaku), containing 25.5% (w/v) PEG 4000, 170 mM ammonium sulfate, and 15% (v/v) glycerol. Crystallization was performed by the sitting drop vapor diffusion technique at 20 °C. For cryoprotection, glycerol concentration was adjusted to 30% (v/v). The X-ray data were obtained at SPring8 BL32XU. PbaB1•PbaA^L^ complex belongs to P 4_3_ space group with unit-cell dimensions *a* = 49.79, *b* = 49.79, and *c* = 135.01 and α = β = γ = 90°. Phenix.Xtriage analysis^79^ indicated the presence of twinning, which resulted in relatively high R-values during further refinement. The structure was solved by molecular replacement using the structure of the protein complex predicted by AlphaFold2 as a search model. The highest-ranked model was used for crystal structure determination. The final structure was refined at 2.0 Å to R_work_ and R_free_ values of 0.23 and 0.26, respectively.

The raw data were processed with XDS software^80^. Molecular replacement was performed by PHASER^81^. For the final structure refinement, PHENIX^79^ and Coot^82^ were employed. Structures were visualized with PyMOL.

### Circular Dichroism (CD) spectroscopy

Coding sequence of PbaB1 was 5’-terminally extended with the 6xHis-tag using PCR and inserted into pET47b vector (Novagen, cat. no. 71461) using InFusion cloning kit to generate pET47-*pbaB1* plasmid.

Protein expression was performed in 2 L of LB medium (Nacalai) supplemented with 30 µg mL^−1^ of kanamycin. Once the cell density reached an OD600 of 0.6, protein expression was induced with 0.1 mM IPTG, and the culture was shifted to 18 °C for 14 h. Cells were collected by centrifugation, resuspended in 80 mL of the buffer WB3 containing 25 mM Tris-HCl (pH 8.0), 150 mM NaCl, 5 mM imidazole (pH 8.0), and lysed by sonication. Cleared lysate, obtained by centrifugation at 20,000 × *g* for 20 min, was applied to a 5 mL HisTrap HP column (Cytiva). After washing the column with the WB3 buffer, PbaB1 was eluted with the elution buffer EB3 (25 mM Tris-HCl (pH 8.0), 150 mM NaCl, 300 mM imidazole (pH 8.0)). The eluted fraction was applied to a HiLoad Superdex 200 16/600 column (Cytiva) pre-equilibrated with 25 mM Tris-HCl (pH 8.0) and 150 mM NaCl. The purified protein was subsequently buffer-exchanged by dialysis against Phosphate Buffered Saline (PBS) (Takara).

The CD spectrum of PbaB1 was recorded on a J-1500 Circular Dichroism Spectrometer (JASCO). The protein concentration was adjusted to 10 μM in PBS buffer, and the sample was loaded into a 1 mm quartz cuvette. Spectra were acquired as ten accumulations at 20 °C over the wavelength range of 200–250 nm with a step size of 0.1 nm and averaged prior to analysis. Source data are provided as a Source Data file.

### NMR spectroscopy

^15^N-labeled PbaB1 protein was produced in M9 minimal medium supplemented with 2 g L^−1 15^NH_4_Cl, 0.5 % glycerol, and 50 µg mL^−1^ of ampicillin and purified as described above.

A sample of 200 µM of ^15^N labeled PbaB1 was dissolved in 180 µL of 50 mM Na phosphate buffer (pH 7.5, 50 mM NaCl), supplemented with 20 µL D_2_O and trimethylsilylpropionate (TSP) as reference, and transferred to a standard 3-mm NMR tube. Titration experiments were performed cumulatively by sequential addition of lyophilized synthetic peptide directly to the NMR sample, with spectra acquisition after each addition. All spectra were recorded at 303 K on an 800-MHz NEO Bruker spectrometer equipped with a QCP cryogenic probe head (Bruker Biospin) and operating under Topspin 4.4.1. The ^1^H, ^15^N HSQC spectrum was recorded with the standard Bruker hsqcf3gpph19 pulse program, with 4k x 128 points for a spectral width of 15.6 ppm x 32 ppm, centered on 4.7 ppm and 119 ppm for the ^1^H and the ^15^N dimensions, respectively, and 32 scans per increment. Spectra were processed to 4k x 512 complex points after zero-filling and multiplication by a squared sine bell window function shifted by π/3. The signal of the TSP was referenced to zero, and indirect referencing was used for the ^15^N dimension. Chemical shift perturbations (CSP values) were calculated using Eq. (1), where ΔδH^N^ represents the difference in the amide proton chemical shift, and ΔδN represents the difference in the nitrogen chemical shift of each peak. They were plotted as a function of ligand concentration to obtain a K_D_ value. All data processing was done using the Bruker Topspin 4.1.3 software. Source data are provided as a Source Data file.

1 $${{\rm{CSP}}}=\sqrt{{(\triangle {{\rm{\delta }}}{{{\rm{H}}}}^{{{\rm{N}}}})}^{2}+0.1{(\triangle {{\rm{\delta }}}{{\rm{N}}})}^{2}}$$

### Reporting summary

Further information on research design is available in the Nature Portfolio Reporting Summary linked to this article.

## Supplementary information

Supplementary Information

Supplementary Data 1

Supplementary Data 2

Supplementary Data 3

Reporting Summary

Transparent Peer Review file

## Source data

Source Data

## Data Availability

Atomic coordinates of LppB•LppA^L^ and PbaB1•PbaA^L^ complexes have been deposited in the Protein Data Bank (PDB) under accession codes pdb_00009x8z [10.2210/pdb9X8Z/pdb] and pdb_00009×90 [10.2210/pdb9X90/pdb]. Atomic coordinates of FusB1•FusA^L^(TfuB1•TfuAL), TbiB1•TbiAα^L^, and TbiB1•TbiAβ^L^ discussed in this study are available under accession codes pdb_00006jx3 [10.2210/pdb6JX3/pdb], pdb_00005v1v [10.2210/pdb5V1V/pdb], and pdb_00005v1u [10.2210/pdb5V1U/pdb], respectively. MALDI MS spectra are available at jPOST and ProteomeXchange under accession numbers JPST004185 and PXD070677, respectively. The NMR data and raw data supporting the phylogenetic analysis are available on Zenodo [10.5281/zenodo.18877617]. Source data are provided with this paper as a Source Data file. Source data are provided with this paper.

## References

[1] Montalbán-López, M. et al. New developments in RiPP discovery, enzymology and engineering. *Nat. Prod. Rep.* **38**, 130–239 (2021). 32935693 10.1039/d0np00027b PMC7864896

[2] Wei, T., Chern, M., Liu, F. & Ronald, P. C. Suppression of bacterial infection in rice by treatment with a sulfated peptide. *Mol. Plant Pathol.* **17**, 1493–1498 (2016). 26765864 10.1111/mpp.12368 PMC6638351

[3] Leprevost, L. et al. A widespread family of ribosomal peptide metallophores involved in bacterial adaptation to metal stress. *Proc. Natl. Acad. Sci. USA* **121**, e2408304121 (2024). 39602266 10.1073/pnas.2408304121 PMC11626156

[4] Wang, B., Zhao, A., Novick, R. P. & Muir, T. W. Key driving forces in the biosynthesis of autoinducing peptides required for staphylococcal virulence. *Proc. Natl. Acad. Sci. USA* **112**, 10679–10684 (2015). 26261307 10.1073/pnas.1506030112 PMC4553796

[5] Okada, M. et al. Structure of the Bacillus subtilis quorum-sensing peptide pheromone ComX. *Nat. Chem. Biol.* **1**, 23–24 (2005). 16407988 10.1038/nchembio709

[6] Kulikovsky, A. et al. Bacillus subtilis utilizes decarboxylated S-adenosylmethionine for the biosynthesis of tandem aminopropylated microcin C, a potent inhibitor of bacterial aspartyl-tRNA synthetase. *J. Am. Chem. Soc.* **147**, 11998–12011 (2025). 40162528 10.1021/jacs.4c18468

[7] Kodani, S. et al. The SapB morphogen is a lantibiotic-like peptide derived from the product of the developmental gene ramS in Streptomyces coelicolor. *Proc. Natl. Acad. Sci. USA* **101**, 11448–11453 (2004). 15277670 10.1073/pnas.0404220101 PMC509221

[8] Allenby, N. E. E. et al. Phosphate starvation induces the sporulation killing factor of bacillus subtilis. *J. Bacteriol.* **188**, 5299–5303 (2006). 16816204 10.1128/JB.00084-06 PMC1539955

[9] Behling, L. A. et al. NMR, mass spectrometry and chemical evidence reveal a different chemical structure for methanobactin that contains oxazolone rings. *J. Am. Chem. Soc.* **130**, 12604–12605 (2008). 18729522 10.1021/ja804747d PMC3617554

[10] Yi, Y., Liang, L., de Jong, A. & Kuipers, O. P. A systematic comparison of natural product potential, with an emphasis on RiPPs, by mining of bacteria of three large ecosystems. *Genomics* **116**, 110880 (2024). 38857812 10.1016/j.ygeno.2024.110880

[11] Pasinato, A. & Singh, G. Bioinformatic exploration of RiPP biosynthetic gene clusters in lichens. *Fungal Biol. Biotechnol.* **12**, 6 (2025). 40317021 10.1186/s40694-025-00197-6 PMC12048977

[12] Ongpipattanakul, C. et al. Mechanism of action of ribosomally synthesized and post-translationally modified peptides. *Chem. Rev.* **122**, 14722–14814 (2022). 36049139 10.1021/acs.chemrev.2c00210 PMC9897510

[13] Arnison, P. G. et al. Ribosomally synthesized and post-translationally modified peptide natural products: overview and recommendations for a universal nomenclature. *Nat. Prod. Rep.* **30**, 108–160 (2013). 23165928 10.1039/c2np20085f PMC3954855

[14] Li, H., Ding, W. & Zhang, Q. Discovery and engineering of ribosomally synthesized and post-translationally modified peptide (RiPP) natural products. *RSC Chem. Biol.* **5**, 90–108 (2024). 38333193 10.1039/d3cb00172e PMC10849128

[15] Wu, C. & van der Donk, W. A. Engineering of new-to-nature ribosomally synthesized and post-translationally modified peptide natural products. *Curr. Opin. Biotechnol.* **69**, 221–231 (2021). 33556835 10.1016/j.copbio.2020.12.022 PMC8238801

[16] Moreira, R., Yang, Y., Luo, Y., Gilmore, M. S. & van der Donk, W. A. Bibacillin 1: a two-component lantibiotic from Bacillus thuringiensis. *RSC Chem. Biol.* **5**, 1060–1073 (2024). 39268544 10.1039/d4cb00192c PMC11385697

[17] Oman, T. J. & van der Donk, W. A. Follow the leader: the use of leader peptides to guide natural product biosynthesis. *Nat. Chem. Biol.* **6**, 9–18 (2010). 20016494 10.1038/nchembio.286 PMC3799897

[18] Burkhart, B. J., Hudson, G. A., Dunbar, K. L. & Mitchell, D. A. A prevalent peptide-binding domain guides ribosomal natural product biosynthesis. *Nat. Chem. Biol.* **11**, 564–570 (2015). 26167873 10.1038/nchembio.1856 PMC4509860

[19] Barrett, S. E. & Mitchell, D. A. Advances in lasso peptide discovery, biosynthesis, and function. *Trends Genet.* **40**, 950–968 (2024). 39218755 10.1016/j.tig.2024.08.002 PMC11537843

[20] Kloosterman, A. M., Shelton, K. E., van Wezel, G. P., Medema, M. H. & Mitchell, D. A. RRE-finder: a genome-mining tool for class-independent RiPP discovery. *mSystems* **5**, 00267-20 (2020). 10.1128/mSystems.00267-20 PMC7470986 32873609

[21] Zhu, S. et al. The B1 protein guides the biosynthesis of a lasso peptide. *Sci. Rep.* **6**, 35604 (2016). 27752134 10.1038/srep35604 PMC5067487

[22] Koos, J. D. & Link, A. J. Heterologous and in vitro reconstitution of fuscanodin, a lasso peptide from thermobifida fusca. *J. Am. Chem. Soc.* **141**, 928–935 (2019). 30532970 10.1021/jacs.8b10724 PMC6475475

[23] Duan, Y. et al. Leader peptide removal in lasso peptide biosynthesis based on penultimate isoleucine residue. *Front. Microbiol.* **14**, 1181125 (2023). 37497541 10.3389/fmicb.2023.1181125 PMC10368454

[24] Zhang, C. & Seyedsayamdost, M. R. CanE, an iron/2-oxoglutarate-dependent lasso peptide hydroxylase from streptomyces canus. *ACS Chem. Biol.* **15**, 890–894 (2020). 32191027 10.1021/acschembio.0c00109

[25] Chekan, J. R., Ongpipattanakul, C. & Nair, S. K. Steric complementarity directs sequence promiscuous leader binding in RiPP biosynthesis. *Proc. Natl. Acad. Sci. USA* **116**, 24049–24055 (2019). 31719203 10.1073/pnas.1908364116 PMC6883790

[26] Li, B. et al. Catalytic promiscuity in the biosynthesis of cyclic peptide secondary metabolites in planktonic marine cyanobacteria. *Proc. Natl. Acad. Sci. USA* **107**, 10430–10435 (2010). 20479271 10.1073/pnas.0913677107 PMC2890784

[27] Nguyen, N. A. et al. A Silent biosynthetic gene cluster from a methanotrophic bacterium potentiates discovery of a substrate promiscuous proteusin cyclodehydratase. *ACS Chem. Biol.* **17**, 1577–1585 (2022). 35666841 10.1021/acschembio.2c00251 PMC9746716

[28] Um, S. et al. Cebulassopins, antiproliferative lasso peptides from a split biosynthetic operon. *J. Am. Chem. Soc*. 10.1021/jacs.5c14658 (2026). 10.1021/jacs.5c14658 41666276

[29] Ellerhorst, M. et al. Recent insights into the biosynthesis and biological activities of the peptide-derived redox cofactor mycofactocin. *Nat. Prod. Rep.* **42**, 1344–1366 (2025). 40375824 10.1039/d5np00012b

[30] Shen, Y.-Q. et al. Distribution and properties of the genes encoding the biosynthesis of the bacterial cofactor, pyrroloquinoline quinone. *Biochemistry* **51**, 2265–2275 (2012). 22324760 10.1021/bi201763d PMC3334298

[31] Tietz, J. I. et al. A new genome-mining tool redefines the lasso peptide biosynthetic landscape. *Nat. Chem. Biol.* **13**, 470–478 (2017). 28244986 10.1038/nchembio.2319 PMC5391289

[32] Zyubko, T. et al. Efficient in vivo synthesis of lasso peptide pseudomycoidin proceeds in the absence of both the leader and the leader peptidase. *Chem. Sci.* **10**, 9699–9707 (2019). 32055339 10.1039/c9sc02370d PMC6993621

[33] Zhu, S. et al. Insights into the unique phosphorylation of the lasso peptide paeninodin. *J. Biol. Chem.* **291**, 13662–13678 (2016). 27151214 10.1074/jbc.M116.722108 PMC4919450

[34] Eslami, S. M. & van der Donk, W. A. Proteases involved in leader peptide removal during RiPP biosynthesis. *ACS bio med chem. Au* **4**, 20–36 (2024). 38404746 10.1021/acsbiomedchemau.3c00059 PMC10885120

[35] NCBI Resource Coordinators Database resources of the National Center for Biotechnology Information. *Nucleic Acids Res.* **44**, D7–D19 (2016). 26615191 10.1093/nar/gkv1290 PMC4702911

[36] Tjalsma, H. et al. Functional analysis of the secretory precursor processing machinery of Bacillus subtilis: identification of a eubacterial homolog of archaeal and eukaryotic signal peptidases. *Genes Dev.* **12**, 2318–2331 (1998). 9694797 10.1101/gad.12.15.2318 PMC317044

[37] Stöver, A. G. & Driks, A. Control of synthesis and secretion of the Bacillus subtilis protein YqxM. *J. Bacteriol.* **181**, 7065–7069 (1999). 10559173 10.1128/jb.181.22.7065-7069.1999 PMC94182

[38] Sun, K. et al. Iterative glycosylation on a single residue of a mature lasso peptide. *Chem. Sci.* **16**, 6480–6487 (2025). 40103722 10.1039/d5sc00605h PMC11912497

[39] Bartholomae, M., Buivydas, A., Viel, J. H., Montalbán-López, M. & Kuipers, O. P. Major gene-regulatory mechanisms operating in ribosomally synthesized and post-translationally modified peptide (RiPP) biosynthesis. *Mol. Microbiol.* **106**, 186–206 (2017). 28787536 10.1111/mmi.13764

[40] Wakeel, M. A., Corbin, E. A., McShan, A. C. & Agarwal, V. Extending the peptide/protein interaction paradigm to a protein/protein engagement model in RiPP biosynthesis. *ACS Chem. Biol.* **20**, 2069–2074 (2025). 40779306 10.1021/acschembio.5c00411 PMC12455567

[41] Dogsa, I. et al. Bacillus subtilis EpsA-O: a novel exopolysaccharide structure acting as an efficient adhesive in biofilms. *npj Biofilms Microbiomes* **10**, 98 (2024). 39358392 10.1038/s41522-024-00555-z PMC11447030

[42] Forrest, D., Warman, E. A., Erkelens, A. M., Dame, R. T. & Grainger, D. C. Xenogeneic silencing strategies in bacteria are dictated by RNA polymerase promiscuity. *Nat. Commun.* **13**, 1149 (2022). 35241653 10.1038/s41467-022-28747-1 PMC8894471

[43] Sumida, T., Dubiley, S., Wilcox, B., Severinov, K. & Tagami, S. Structural basis of leader peptide recognition in lasso peptide biosynthesis pathway. *ACS Chem. Biol.* **14**, 1619–1627 (2019). 31188556 10.1021/acschembio.9b00348

[44] Craveur, P., Joseph, A. P., Rebehmed, J. & de Brevern, A. G. β-Bulges: extensive structural analyses of β-sheets irregularities. *Protein Sci.* **22**, 1366–1378 (2013). 23904395 10.1002/pro.2324 PMC3795495

[45] Kretsch, A. M. et al. Peptidase activation by a leader peptide-bound RiPP recognition element. *Biochemistry* **62**, 956–967 (2023). 36734655 10.1021/acs.biochem.2c00700 PMC10126823

[46] Duan, Y. et al. Heterologous expression and *in vivo* characterization of the longipeptin biosynthetic gene cluster. *Org. Biomol. Chem*. 10.1039/D6OB00049E (2026). 10.1039/d6ob00049e 41769935

[47] Williamson, M. P. Using chemical shift perturbation to characterise ligand binding. *Prog. Nucl. Magn. Reson. Spectrosc.* **73**, 1–16 (2013). 23962882 10.1016/j.pnmrs.2013.02.001

[48] Duquesne, S. et al. Two enzymes catalyze the maturation of a lasso peptide in Escherichia coli. *Chem. Biol.* **14**, 793–803 (2007). 17656316 10.1016/j.chembiol.2007.06.004

[49] Alfi, A. et al. Cell-free mutant analysis combined with structure prediction of a lasso peptide biosynthetic protease B2. *ACS Synth. Biol.* **11**, 2022–2028 (2022). 35674818 10.1021/acssynbio.2c00176

[50] Shimizu, Y., Kanamori, T. & Ueda, T. Protein synthesis by pure translation systems. *Methods* **36**, 299–304 (2005). 16076456 10.1016/j.ymeth.2005.04.006

[51] Abramson, J. et al. Accurate structure prediction of biomolecular interactions with AlphaFold 3. *Nature* **630**, 493–500 (2024). 38718835 10.1038/s41586-024-07487-w PMC11168924

[52] Karamycheva, S., Wolf, Y. I., Koonin, E. V. & Makarova, K. S. Spatial-temporal genome analysis and its application for the prediction of functional systems in bacteria and archaea. *mBio* **17**, 03127 (2026). 10.1128/mbio.03127-25 PMC12802224 41395940

[53] Puigbò, P., Makarova, K. S., Kristensen, D. M., Wolf, Y. I. & Koonin, E. V. Reconstruction of the evolution of microbial defense systems. *BMC Evol. Biol.* **17**, 94 (2017). 28376755 10.1186/s12862-017-0942-y PMC5379612

[54] Nakamura, L. K. Bacillus alginolyticus sp. nov. and Bacillus chondroitinus sp. nov., Two Alginate-Degrading Species. *Int. J. Syst. Bacteriol.* **37**, 284–286 (1987).

[55] Sayers, E. W. et al. Database resources of the national center for biotechnology information. *Nucleic Acids Res* **50**, D20–D26 (2022). 34850941 10.1093/nar/gkab1112 PMC8728269

[56] Camacho, C. et al. BLAST+: architecture and applications. *BMC Bioinforma.* **10**, 421 (2009). 10.1186/1471-2105-10-421 PMC2803857 20003500

[57] Oberg, N., Zallot, R. & Gerlt, J. A. EFI-EST, EFI-GNT, and EFI-CGFP: enzyme function initiative (EFI) web resource for genomic enzymology tools. *J. Mol. Biol.* **435**, 168018 (2023). 37356897 10.1016/j.jmb.2023.168018 PMC10291204

[58] Shannon, P. et al. Cytoscape: a software environment for integrated models of biomolecular interaction networks. *Genome Res* **13**, 2498–2504 (2003). 14597658 10.1101/gr.1239303 PMC403769

[59] El-Gebali, S. et al. The Pfam protein families database in 2019. *Nucleic Acids Res.* **47**, D427–D432 (2019). 30357350 10.1093/nar/gky995 PMC6324024

[60] Haft, D. H. et al. TIGRFAMs: a protein family resource for the functional identification of proteins. *Nucleic Acids Res.* **29**, 41–43 (2001). 11125044 10.1093/nar/29.1.41 PMC29844

[61] Madeira, F. et al. The EMBL-EBI Job Dispatcher sequence analysis tools framework in 2024. *Nucleic Acids Res.* **52**, W521–W525 (2024). 38597606 10.1093/nar/gkae241 PMC11223882

[62] Bailey, T. L. et al. MEME SUITE: tools for motif discovery and searching. *Nucleic Acids Res.* **37**, W202–W208 (2009). 19458158 10.1093/nar/gkp335 PMC2703892

[63] Crooks, G. E., Hon, G., Chandonia, J.-M. & Brenner, S. E. WebLogo: a sequence logo generator. *Genome Res.* **14**, 1188–1190 (2004). 15173120 10.1101/gr.849004 PMC419797

[64] Schneider, T. D. & Stephens, R. M. Sequence logos: a new way to display consensus sequences. *Nucleic Acids Res.* **18**, 6097–6100 (1990). 2172928 10.1093/nar/18.20.6097 PMC332411

[65] Edgar, R. C. Muscle5: High-accuracy alignment ensembles enable unbiased assessments of sequence homology and phylogeny. *Nat. Commun.* **13**, 6968 (2022). 36379955 10.1038/s41467-022-34630-w PMC9664440

[66] Price, M. N., Dehal, P. S. & Arkin, A. P. FastTree 2-approximately maximum-likelihood trees for large alignments. *PLoS One* **5**, e9490 (2010). 20224823 10.1371/journal.pone.0009490 PMC2835736

[67] Letunic, I. & Bork, P. Interactive Tree of Life (iTOL) v6: recent updates to the phylogenetic tree display and annotation tool. *Nucleic Acids Res.* **52**, W78–W82 (2024). 38613393 10.1093/nar/gkae268 PMC11223838

[68] Makarova, K. S. et al. An updated evolutionary classification of CRISPR–Cas systems including rare variants. *Nat. Microbiol.* **10**, 3346–3361 (2025). 41198952 10.1038/s41564-025-02180-8 PMC12669027

[69] Steinegger, M. & Söding, J. MMseqs2 enables sensitive protein sequence searching for the analysis of massive data sets. *Nat. Biotechnol.* **35**, 1026–1028 (2017). 29035372 10.1038/nbt.3988

[70] Fitch, W. M. Toward defining the course of evolution: minimum change for a specific tree topology. *Syst. Zool.* **20**, 406 (1971).

[71] Jumper, J. et al. Highly accurate protein structure prediction with AlphaFold. *Nature* **596**, 583–589 (2021). 34265844 10.1038/s41586-021-03819-2 PMC8371605

[72] Evans, R. et al. Protein complex prediction with AlphaFold-Multimer. Preprint at 10.1101/2021.10.04.463034 (2021).

[73] Larionov, A., Krause, A. & Miller, W. A standard curve based method for relative real time PCR data processing. *BMC Bioinforma.* **6**, 62 (2005). 10.1186/1471-2105-6-62 PMC1274258 15780134

[74] Ho, S. N., Hunt, H. D., Horton, R. M., Pullen, J. K. & Pease, L. R. Site-directed mutagenesis by overlap extension using the polymerase chain reaction. *Gene* **77**, 51–59 (1989). 2744487 10.1016/0378-1119(89)90358-2

[75] Pomerantsev, A. P., Camp, A. & Leppla, S. H. A new minimal replicon of Bacillus anthracis plasmid pXO1. *J. Bacteriol.* **191**, 5134–5146 (2009). 19502400 10.1128/JB.00422-09 PMC2725576

[76] Okuda, S. et al. jPOST environment accelerates the reuse and reanalysis of public proteome mass spectrometry data. *Nucleic Acids Res.* **53**, D462–D467 (2025). 39526391 10.1093/nar/gkae1032 PMC11701591

[77] Shevchenko, A., Tomas, H., Havli, J., Olsen, J. V. & Mann, M. In-gel digestion for mass spectrometric characterization of proteins and proteomes. *Nat. Protoc.* **1**, 2856–2860 (2006). 17406544 10.1038/nprot.2006.468

[78] Perkins, D. N., Pappin, D. J. C., Creasy, D. M. & Cottrell, J. S. Probability-based protein identification by searching sequence databases using mass spectrometry data. *Electrophoresis* **20**, 3551–3567 (1999). 10612281 10.1002/(SICI)1522-2683(19991201)20:18<3551::AID-ELPS3551>3.0.CO;2-2

[79] Liebschner, D. et al. Macromolecular structure determination using X-rays, neutrons and electrons: recent developments in *Phenix*. *Acta Crystallogr. D. Struct. Biol.* **75**, 861–877 (2019). 31588918 10.1107/S2059798319011471 PMC6778852

[80] Kabsch, W. *XDS*. *Acta Crystallogr. D Biol. Crystallogr*. **66**, 125–132 (2010). 10.1107/S0907444909047337 PMC2815665 20124692

[81] McCoy, A. J. et al. Phaser crystallographic software. *J. Appl. Crystallogr.* **40**, 658–674 (2007). 19461840 10.1107/S0021889807021206 PMC2483472

[82] Emsley, P., Lohkamp, B., Scott, W. G. & Cowtan, K. Features and development of *Coot*. *Acta Crystallogr. D. Biol. Crystallogr.* **66**, 486–501 (2010). 20383002 10.1107/S0907444910007493 PMC2852313

