# Supplementary material for: RiPP recognition elements evolved to prevent pathway interference through leader peptide discrimination
Source: Nat Commun. 2026 May 20;17:6633. doi: 10.1038/s41467-026-73250-6 (PMC13381572; doi:10.1038/s41467-026-73250-6)
Supplement: Supplementary file 1 — Supplementary Information [file 41467_2026_73250_MOESM1_ESM.pdf]

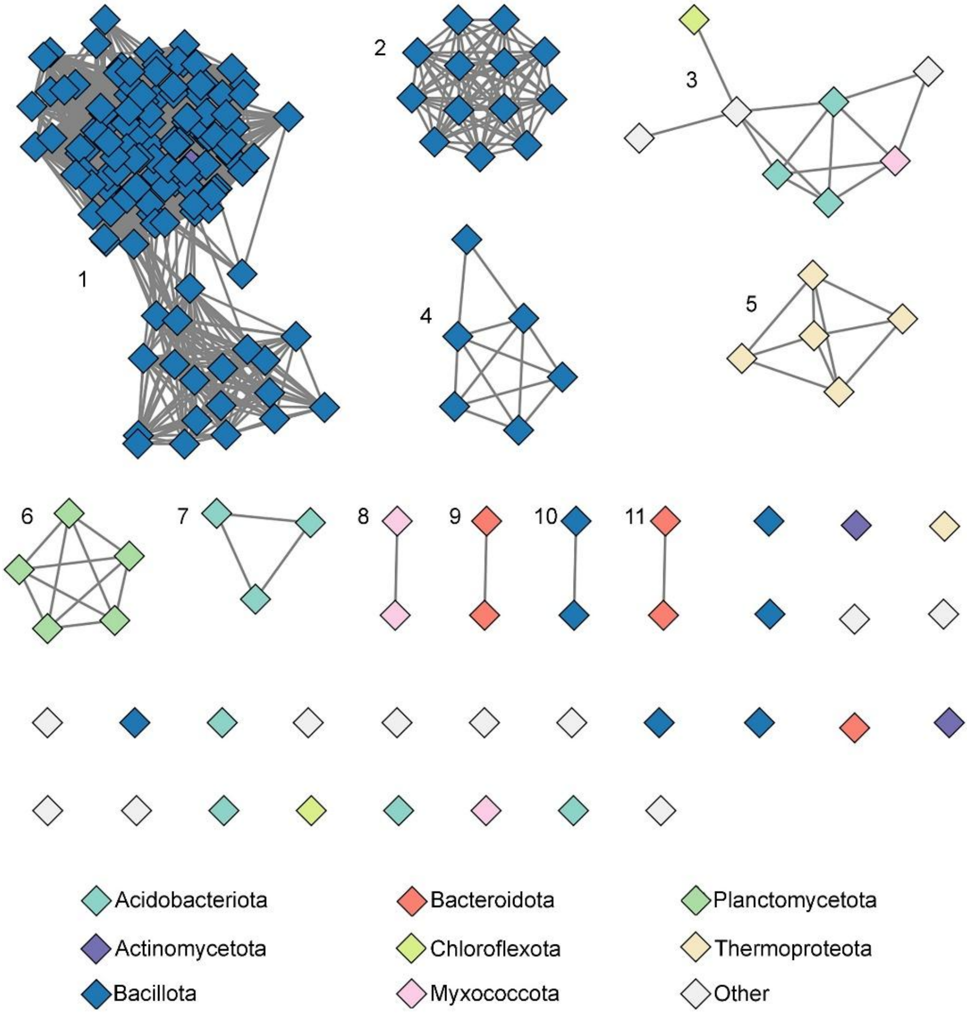


**Supplementary Figure 1. Sequence similarity network of proteins, homologous to the signal peptidase I from the *lpp* biosynthetic gene cluster of *Paenibacillus alginolyticus* DSM5050 (WP_029196374.1).** Edges corresponding to an E-value higher than 10e-23 are removed. Nodes are colored according to the phylum.

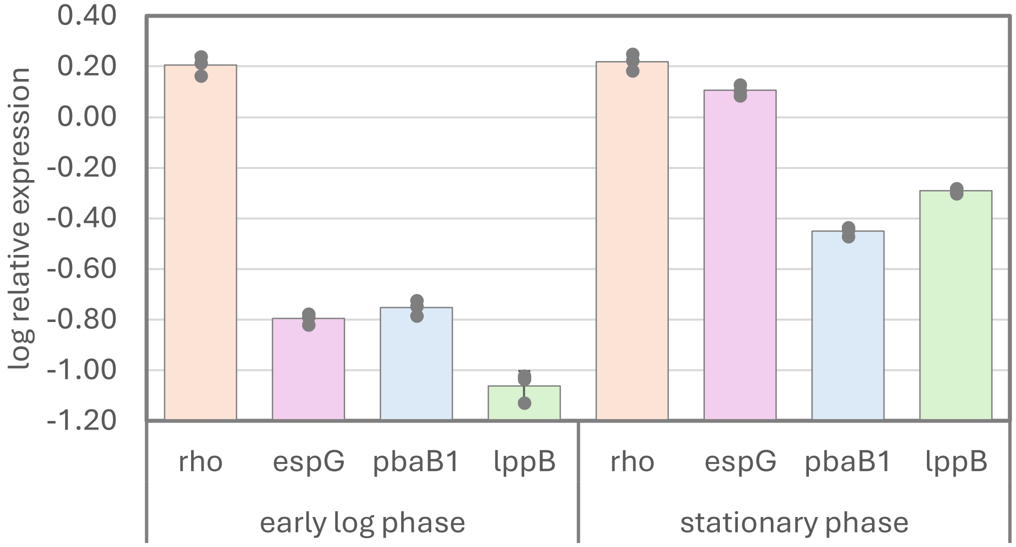


**Supplementary Figure 2. RT-qPCR analysis of *lpp* and *pba* BGCs expression levels in *Paenibacillus alginolyticus* DSM5050 cells harvested at early logarithmic and stationary growth phases in 1/2 LB medium.** Data were normalized to the expression level of the constitutively expressed *gyrA*. Expression of *rho* was used as a positive technical control. *espG* (PAL01S_RS16325) is the exopolysaccharide chain length-determining protein tyrosine kinase gene colocalized with *lpp* and *pba* (Supplementary Figure 3). Data from 3 individual replicates are shown as dots. Source data are provided as a Source Data file.

**A**

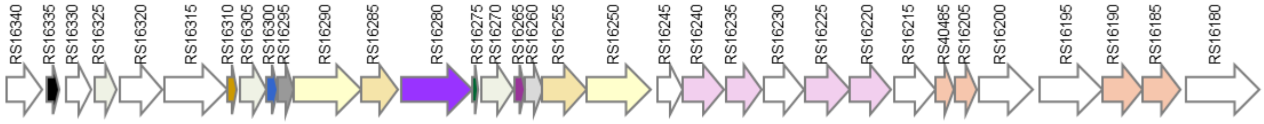


**B**

| Locus tag | Protein family | Annotation | Name |
|---|---|---|---|
| PAL01S_RS16340 | TIGR00277 | HDIG: HDIG domain | |
| PAL01S_RS16335 | TIGR03020 | EpsA: transcriptional regulator EpsA | |
| PAL01S_RS16330 | TIGR01006 | polys_exp_MPA1: polysaccharide export protein, MPA1 family | |
| PAL01S_RS16325 | TIGR03029 | EpsG: chain length determinant protein tyrosine kinase EpsG | |
| PAL01S_RS16320 | TIGR01730 | RND_mfp: efflux transporter, RND family, MFP subunit | |
| PAL01S_RS16310 | No match | | |
| PAL01S_RS16315 | No match | | *lppA*$^{LC}$ |
| PAL01S_RS16305 | TIGR04352 | HprK_rel_A: HprK-related kinase A | *lppK* |
| PAL01S_RS16300 | RREFam002 | Discrete RRE protein in a lasso peptide cluster | *lppB* |
| PAL01S_RS16295 | TIGR02228 | sigpep_I_arch: signal peptidase I | *lppS* |
| PAL01S_RS16290 | TIGR02204 | MsbA_rel: ABC transporter, permease/ATP-binding protein | *lppD* |
| PAL01S_RS16285 | PF14907 | Uncharacterized nucleotidyltransferase | *lppN* |
| PAL01S_RS16280 | TIGR01536 | asn_synth_AEB: asparagine synthase (glutamine-hydrolyzing) | *pbaC* |
| PAL01S_RS16275 | No match | | *pbaA*$^{LC}$ |
| PAL01S_RS16270 | TIGR04352 | HprK_rel_A: HprK-related kinase A | *pbaK* |
| PAL01S_RS16265 | RREFam002 | Discrete RRE protein in a lasso peptide cluster | *pbaB1* |
| PAL01S_RS16260 | PF13471 | Transglutaminase-like superfamily | *pbaB2* |
| PAL01S_RS16255 | PF14907 | Uncharacterized nucleotidyltransferase | *pbaN* |
| PAL01S_RS16250 | TIGR02203 | MsbA_lipidA: lipid A export permease/ATP-binding protein MsbA | *pbaD* |
| PAL01S_RS16245 | PF05704 | Capsular polysaccharide synthesis protein | |
| PAL01S_RS16240 | PF00534 | Glycosyl transferases group 1 | |
| PAL01S_RS16235 | PF00535 | Glycosyl transferase family 2 | |
| PAL01S_RS16230 | PF04230 | Polysaccharide pyruvyl transferase | |
| PAL01S_RS16225 | PF00534 | Glycosyl transferases group 1 | |
| PAL01S_RS16220 | PF00534 | Glycosyl transferases group 1 | |
| PAL01S_RS16215 | PF07470 | Glycosyl Hydrolase Family 88 | |
| PAL01S_RS40485 | TIGR03570 | sugar O-acyltransferase, sialic acid O-acetyltransferase, NeuD family | |
| PAL01S_RS16205 | TIGR03570 | sugar O-acyltransferase, sialic acid O-acetyltransferase, NeuD family | |
| PAL01S_RS16200 | No match | | |
| PAL01S_RS16195 | TIGR01923 | O-succinylbenzoate-CoA ligase | |
| PAL01S_RS16190 | PF01757 | Acyltransferase family | |
| PAL01S_RS16185 | PF01757 | Acyltransferase family | |
| PAL01S_RS16180 | PF13229 | Right-handed beta helix region | |

**Supplementary Figure 3. Schematic representation of the *P. alginolyticus lpp* locus (A) and annotations of the genes (B).** Arrows indicating genes are colored according to their putative functions. Genes used for the RT-PCR assay (Figure S2) are highlighted in blue.

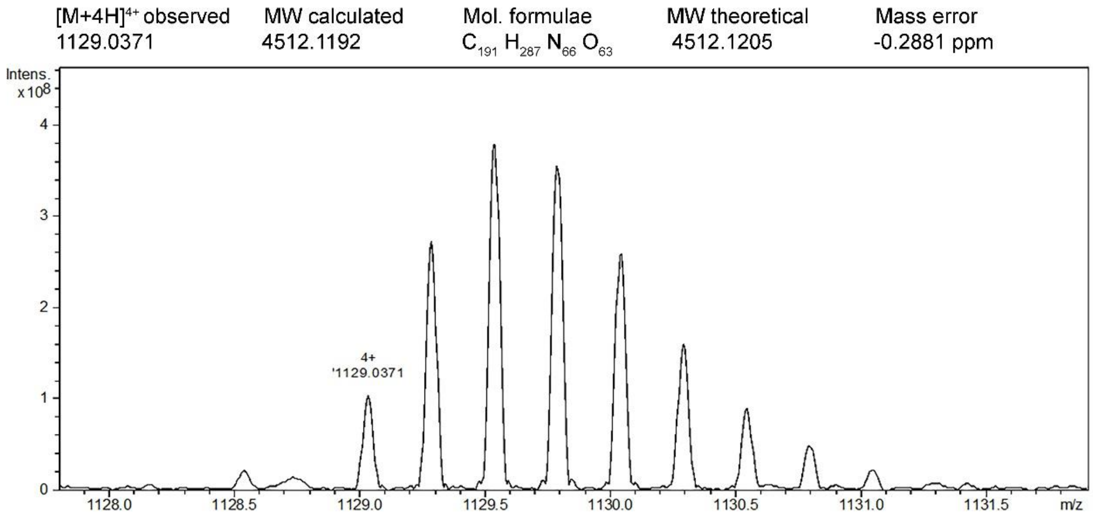


**Supplementary Figure 4. High-resolution MS spectrum of the *in vivo* processed LppA$^{LC}$.**

**A**

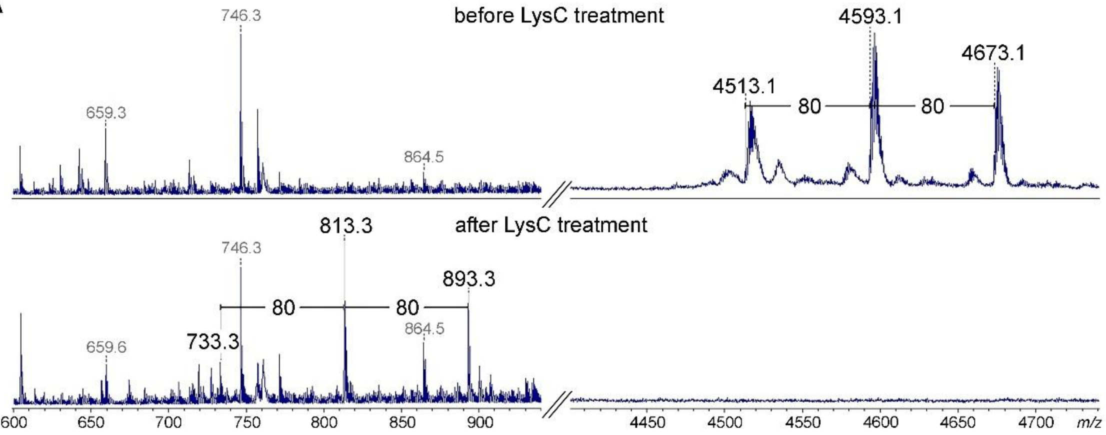


**B**

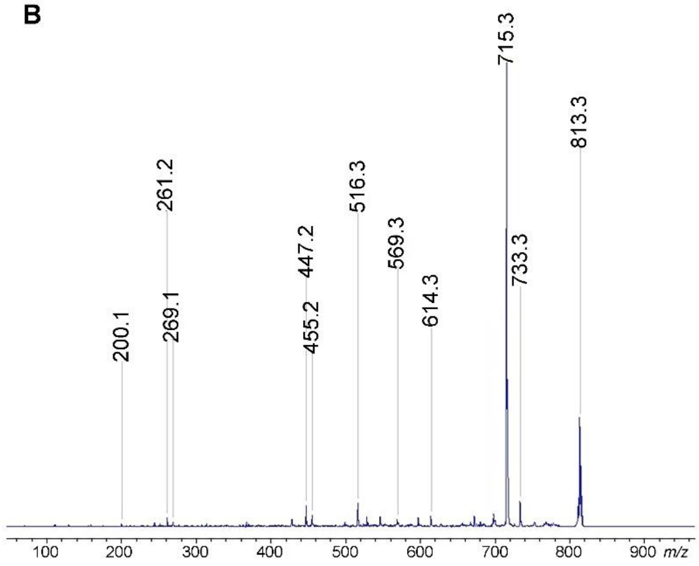


| $[M+H]^+$ | | fragment N-C |
|---|---|---|
| 200.1 | b2 | AQ |
| 261.2 | y"2 | NK |
| 269.1 | b3-$H_3PO_4$ | AQdS* |
| 447.2 | y"3 | WNK |
| 455.2 | b4-$H_3PO_4$ | AQdSW |
| 516.3 | y"4-$H_3PO_4$ | dSWNK |
| 569.3 | b5-$H_3PO_4$ | AQdSWN |
| 614.3 | y"4 | SpWNK** |
| 715.3 | parent-$H_3PO_4$ | AQdSWNK |
| 733.3 | parent-$HPO_3$ | AQSWNK |
| 813.3 | parent | AQSpWNK |

*dS - dehydrated Ser; **Sp -phosphorylated Ser

**Supplementary Figure 5. Identification of the phosphorylation site in LppA$^{LC}$. A.** MALDI-TOF MS spectra of partially purified products of *lpp* BGC before and after treatment with LysC endoprotease recorded in reflector mode. $[M+H]^+$ mass peaks at *m/z* 4513.1, 4593.1, and 4673.1 correspond to the unphosphorylated, mono-, and di-phosphorylated LppA$^C$ peptide. $[M+H]^+$ mass peaks at *m/z* 733.3, 813.3, and 893.3 correspond to the same peptides with the C-terminal part removed by LysC. Spectra were recorded in reflector mode with measurement accuracy within 0.1 Da. **B.** MALDI TOF MS/MS analysis of the N-terminal fragment of the monophosphorylated LppA$^C$ peptide ($[M+H]^+$ 813.3).

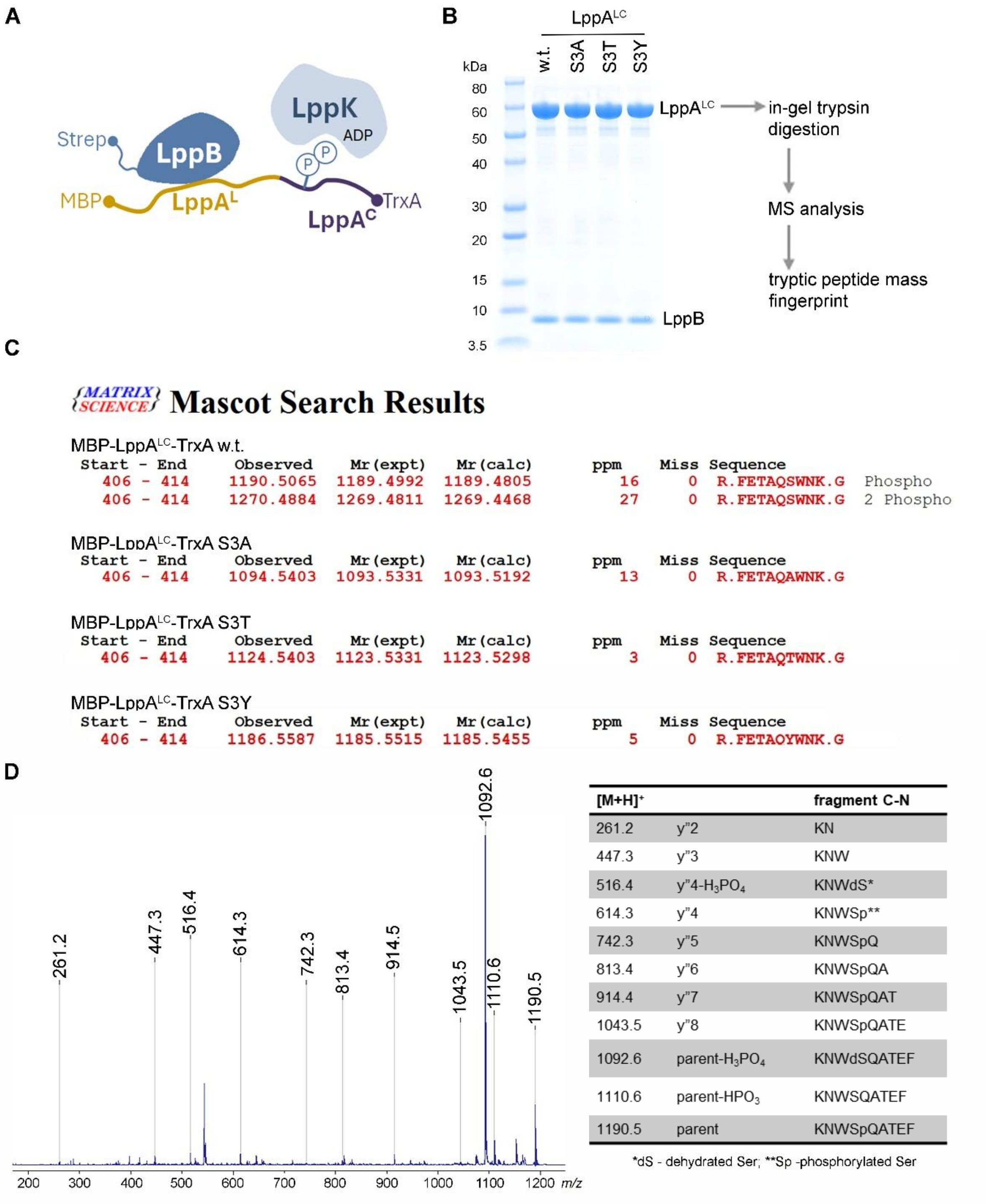


**Supplementary Figure 6. LppK kinase modifies conserved Ser3 residue in LppA$^{LC}$. A**. Schematic representation of the assay. LppA$^{LC}$ peptide fused with N-terminal MBP and C-terminal TrxA mass tag (MW 60.1 kDa) co-expressed with LppB harboring N-terminal Strep-tag and LppK. The LppB-LppA$^{LC}$ complex was purified using affinity chromatography. **B.** SDS-PAGE analysis

of the eluted fractions. Mutations in $LppA^{LC}$ are shown at the top. Protein zones corresponding to $LppA^{LC}$ (MBP-$LppA^{LC}$-TrxA fused protein, MW 61.5 kDa) were subjected to in-gel digestion with trypsin. The eluted tryptic peptides were identified using MALDI-TOF MS. All pull-down experiments were performed independently three times with similar results. Source data are provided as a Source Data file. **C.** Protein modifications analysis using the Mascot protein identification tool (https://www.matrixscience.com/) with phosphorylation as a variable modification. Shown are extracts of the relevant peptide hits from the Mascot Peptide Summary. **D.** MALDI-TOF MS/MS analysis of the monophosphorylated tryptic peptide of $LppA^{LC}$ ($[M+H]^+$ 1190.5).

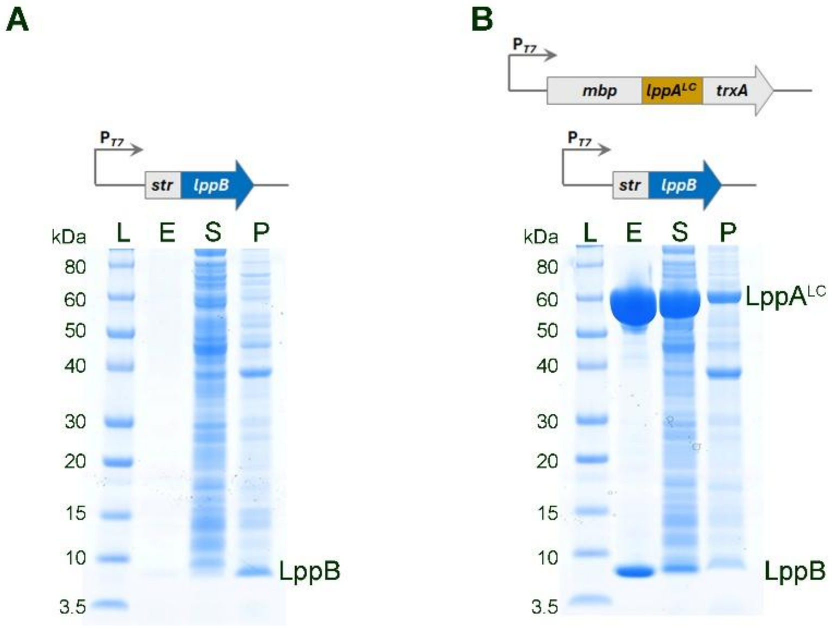


**Supplementary Figure 7. Purification of LppB (A) and the LppB•LppA$^{LC}$ complex (B).** LppA$^{LC}$ was fused to MBP and TrxA mass tags to improve resolution on PAGE; LppB was fused with Strep tag. L, protein MW molecular weight ladder; E, eluate from StrepTactin resin; S, cleared cellular lysate; P, insoluble fraction. LppA$^{LC}$, MBP-LppA$^{LC}$-TrxA fused protein, MW 61.5 kDa; LppB, Strep-tagged LppB, MW 11.1 kDa. All pull-down experiments were performed independently three times with similar results. Source data are provided as a Source Data file.

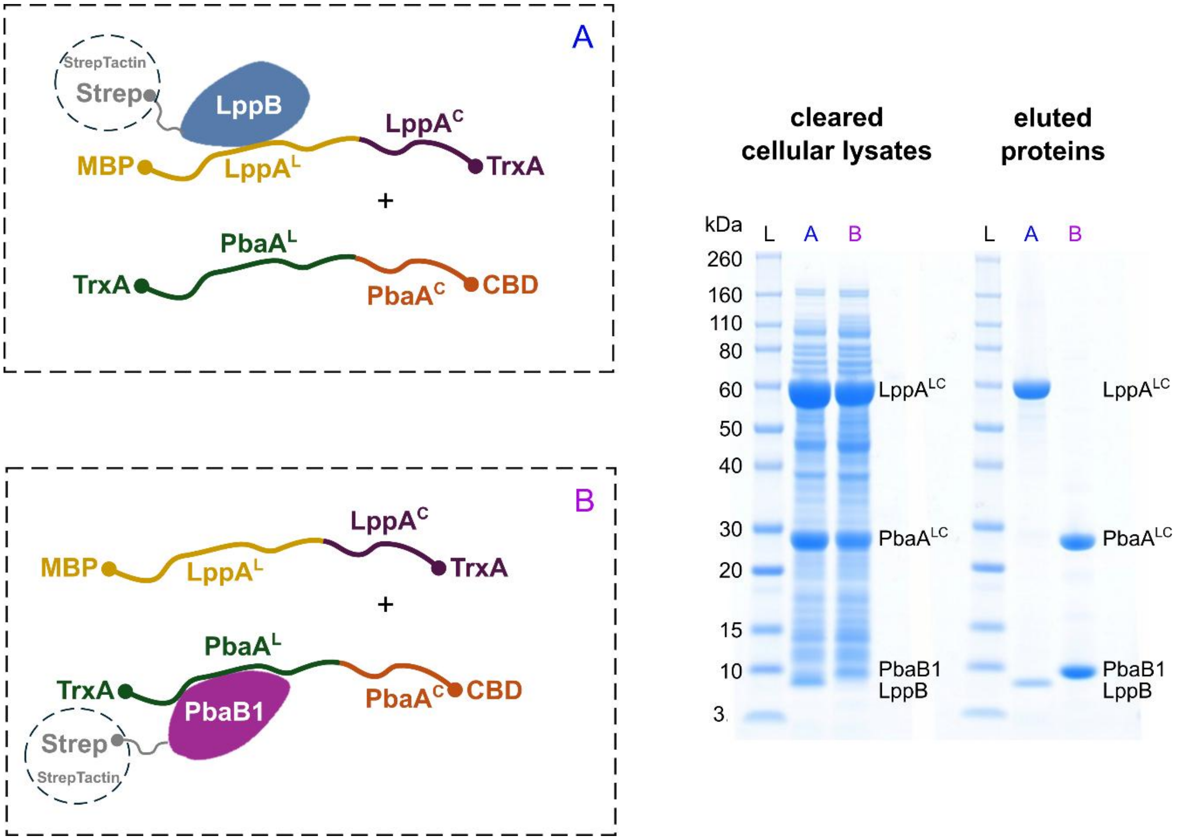


**Supplementary Figure 8.** Schematic representation of the competition pull-down assay (left) and cleared cellular lysates and eluted protein fractions of the competition pull-down assays of RRE-precursor peptide complexes co-expressing LppA$^{LC}$ and PbaA$^{LC}$ peptides with either Strep-tagged LppB (A) or Strep-tagged PbaB1 (B) (right). LppA$^{LC}$ was expressed as an MBP-LppA$^{LC}$-TrxA fusion (MW 61.5 kDa), and PbaA$^{LC}$ as a TrxA-PbaA$^{LC}$-CBD fusion (MW 24.6 kDa). L, protein MW ladder. All pull-down experiments were performed independently three times with similar results. Source data are provided as a Source Data file.

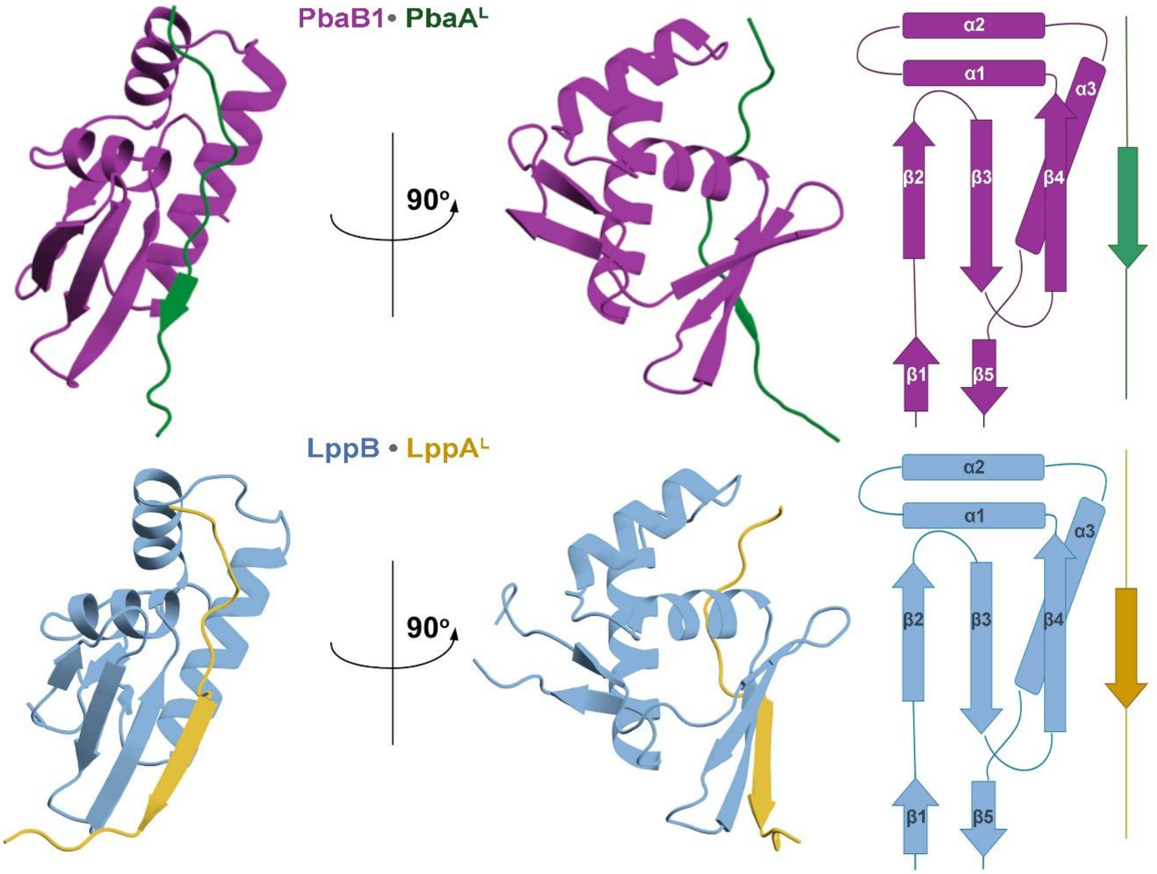


**Supplementary Figure 9. Crystal structures and topology diagrams of the PbaB1•PbaA$^L$ (top) and LppB•LppA$^L$ (bottom) complexes.** RRE proteins are shown in purple (PbaB1) or light blue (LppB), while their cognate leader peptides are depicted in green (PbaA$^L$) or yellow (LppA$^L$).

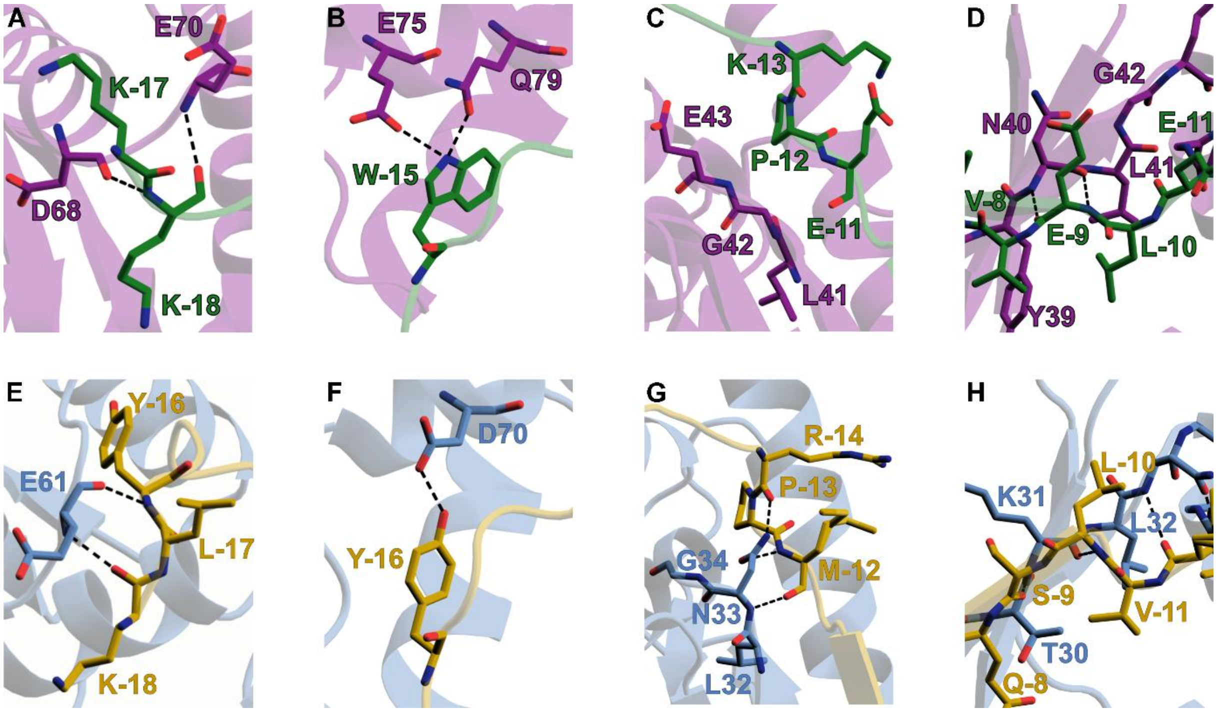


**Supplementary Figure 10. Intermolecular interactions in the PbaB1•PbaA$^{L}$ (A-D) and LppB•LppA$^{L}$ (E-H) complexes.** Residues in the PbaA$^{L}$ and LppA$^{L}$ leader peptides are shown in green and yellow, respectively. PbaB1 and LppB RRE domains are in magenta and blue, respectively. Hydrogen bonds are indicated with black dashed lines. Alternative conformations are shown for LppA$^{L}$ M-12.

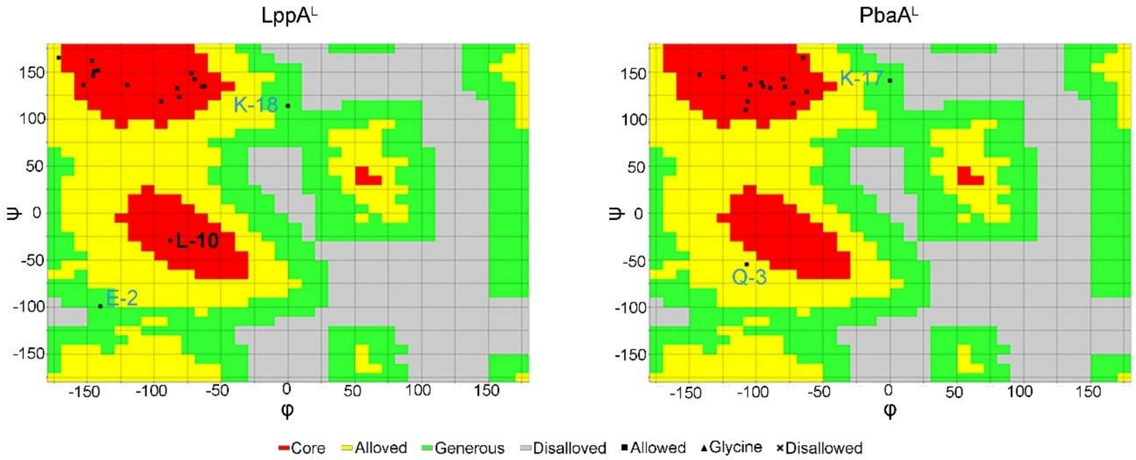


**Supplementary Figure 11. Ramachandran plots of the LppA$^{L}$ and PbaA$^{L}$ peptides.** Coordinates for the peptides were obtained from the pdb_00009x8z (LppB•LppA$^{L}$) and pdb_00009x90 (PbaB•PbaA$^{L}$) structures and analyzed using the VADAR protein structure analysis server[1] (http://vadar.wishartlab.com/). Residues with φ,ψ angles outside the β sheet region are indicated. The first and last residues of each leader peptide visible in the corresponding crystal structures are highlighted in blue.

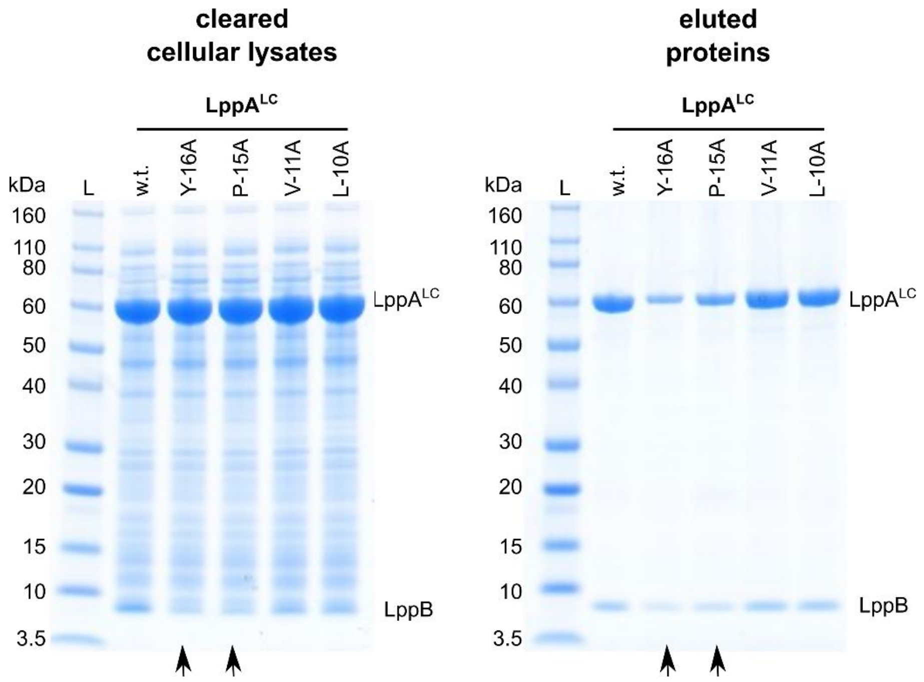


**Supplementary Figure 12.** Cleared cellular lysates (left) and eluted protein fractions (right) from pull-down assays of LppB in complex with wild-type or mutant $LppA^{LC}$. $LppA^{LC}$ was expressed as an MBP-$LppA^{LC}$-TrxA fusion protein (MW 61.5 kDa). L, molecular weight ladder. Arrows indicate samples in which LppB is largely insoluble in the absence of a bound peptide, resulting in reduced levels in the cleared lysate (left) and consequently decreased recovery of the complex (right). All pull-down experiments were performed independently three times with similar results. Source data are provided as a Source Data file.

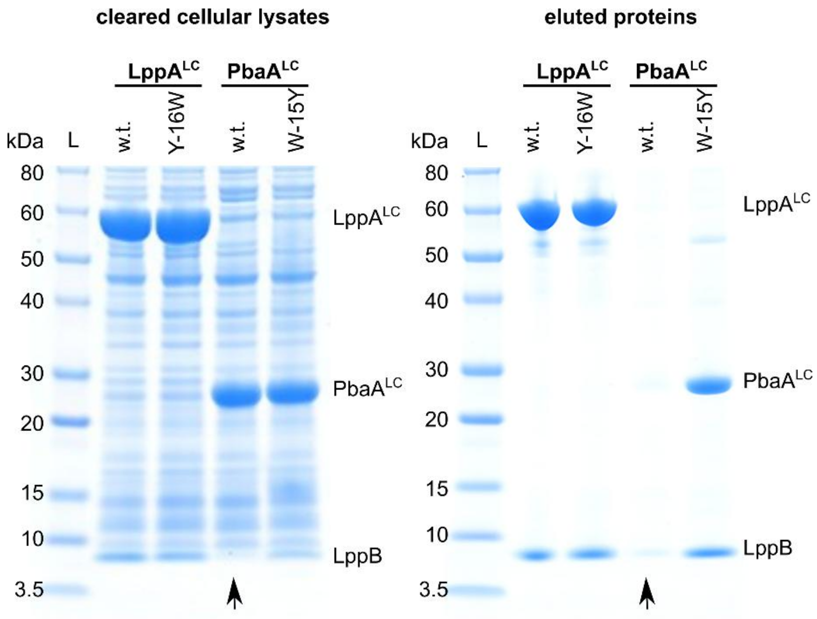


**Supplementary Figure 13**. Cleared cellular lysates (left) and eluted protein fractions (right) from pull-down assays of LppB in complex with wild-type or mutant $LppA^{LC}$ and $PbaA^{LC}$ peptides. $LppA^{LC}$ was expressed as an MBP–$LppA^{LC}$–TrxA fusion (MW 61.5 kDa), and $PbaA^{LC}$ as a TrxA–$PbaA^{LC}$–CBD fusion (MW 24.6 kDa). Arrows indicate samples in which LppB remains predominantly insoluble in the absence of a bound peptide, leading to diminished levels in the cleared lysate and correspondingly lower recovery of the complex. L, molecular weight ladder. All pull-down experiments were performed independently three times with similar results. Source data are provided as a Source Data file.

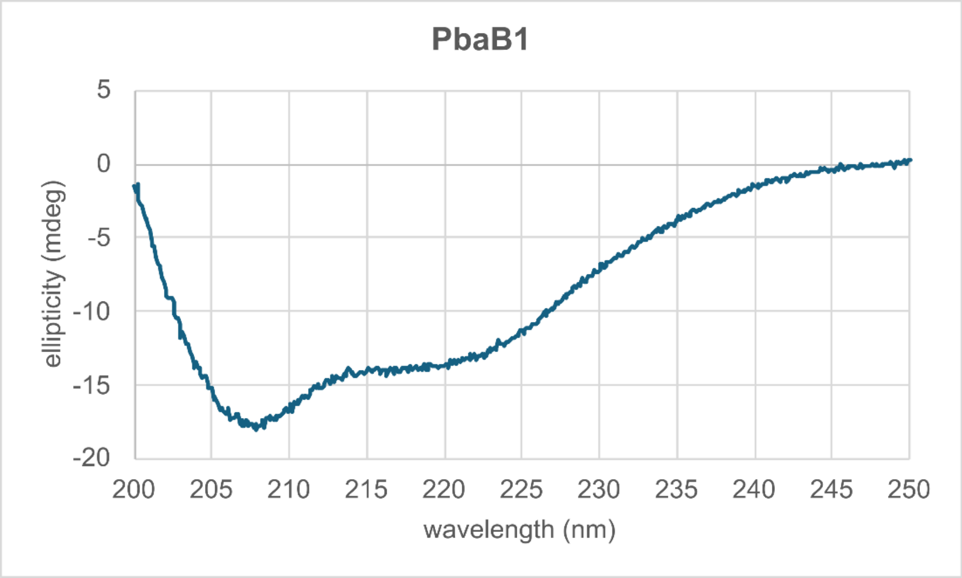


**Supplementary Figure 14. Circular dichroism (CD) spectra of PbaB1 recorded at 20 °C.** Source data are provided as a Source Data file.

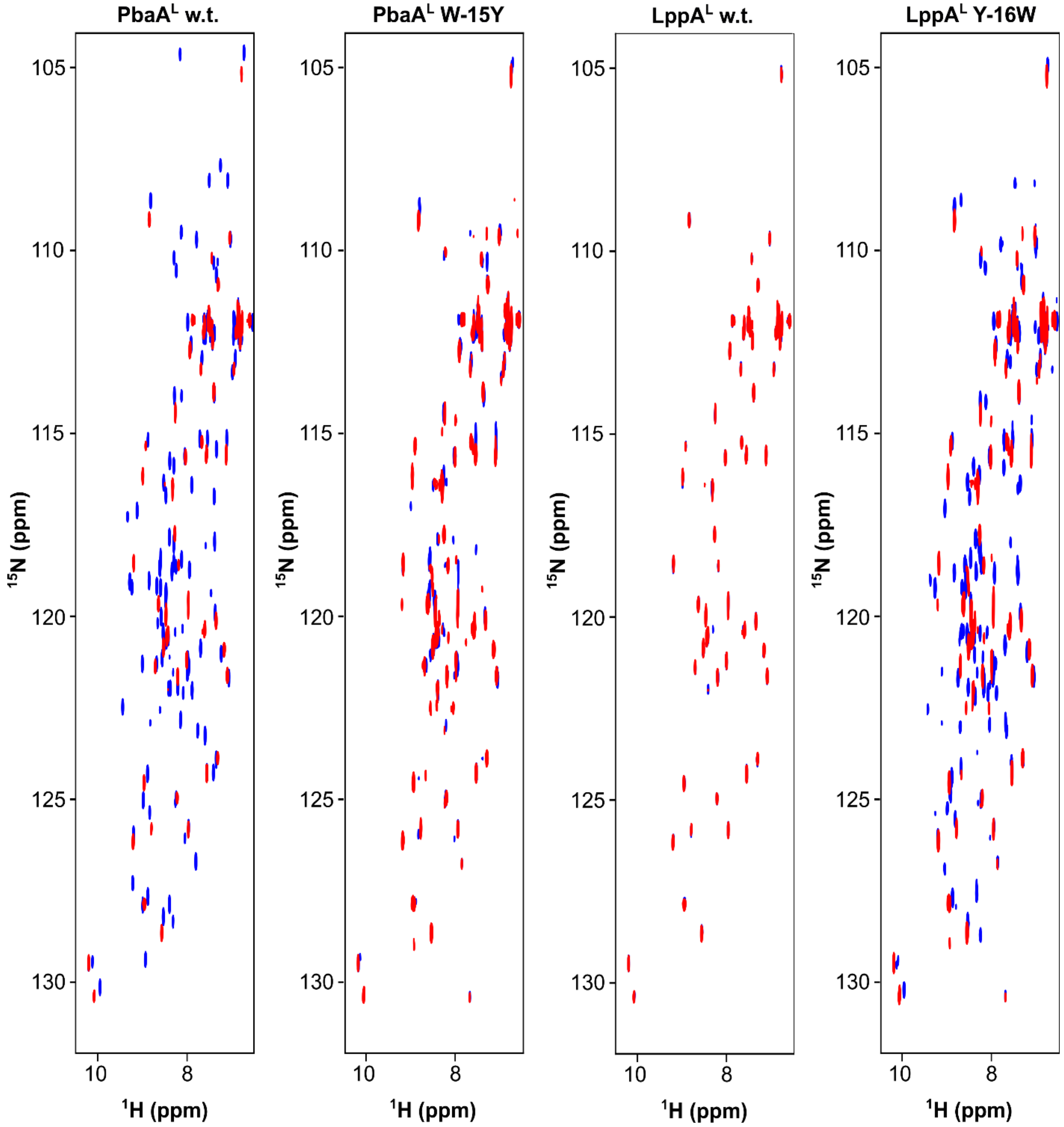


**Supplementary Figure 15.** **$^{1}$H, $^{15}$N HSQC spectra of the isolated PbaB1 RRE domain (red), and of the same domain in the presence of the indicated peptides in equimolar concentration (blue).**

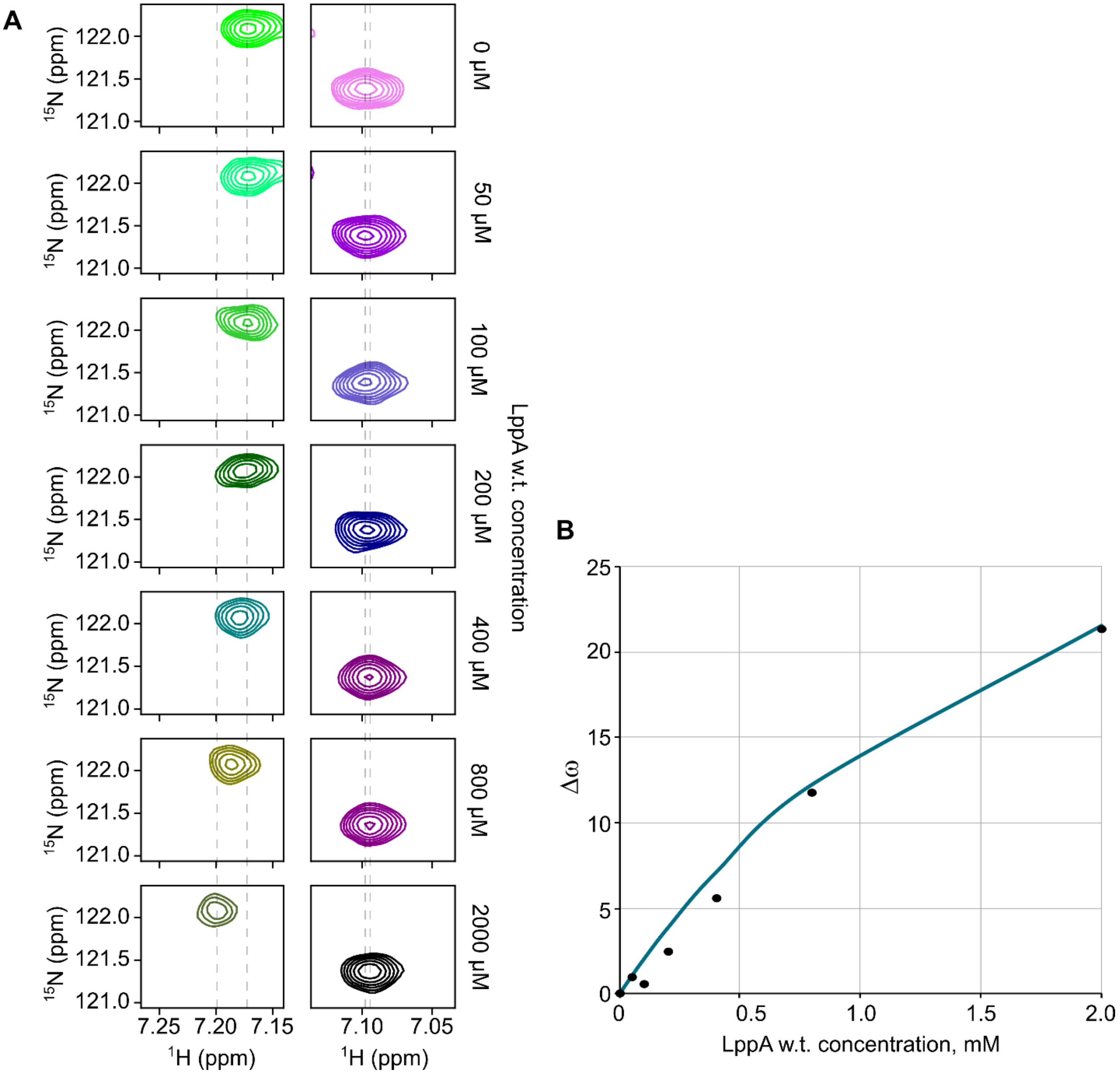


**Supplementary Figure 16. (A)** $^{1}$H,$^{15}$N HSQC spectra of the PbaB1 RRE domain recorded in the presence of increasing concentrations of the LppA$^{L}$ peptide. **(B)** The chemical shift perturbation of the left resonance was fitted to a 1:1 binding model, yielding a $K_D$ of 1.5 mM. Source data are provided as a Source Data file.

| Protein ID | LP | Phylum | RiPP | Sequence |
|---|---|---|---|---|
| WP_011291591.1 (FusB1) | YxxP | Actinomycetota | lasso | -------------METTGAEFRLRPEISVAQTDYGMVLLDGRSGEYWQINDTAALIVQRLLDGHSPADVAQFLTSEYEVERTDAERDIAALVTSLKENGMALP--------------------- |
| WP_011437370.1 | YxxP | Actinomycetota | lasso | -------------------MVTLRPDVVRAPTQYGAVLLHIDNGRYWTINPSGDLVLRILLDGGDTAAAVRGLCETSEVDPETARRDVEGLLAQLADVGLIEAEPESRWSPETEAVCDPRTQAR |
| WP_184223909.1 | YxxP | Acidobacteriota | lasso | --------MMNGSS---HLR------TIV--NQDGAAVLDTKLGSIATINSTGAYVWQSLERGDTLEVIIANLSREAETPHKTVERDVLEFVEALRAQQLLSH--------------------- |
| VJB82892.1 | YxxP | Bacillota | lasso | --------MFNKESK---------SRLKIIKQDTGAIVFDKNQGIYFQTNDVGVKILELLSKNESEEEIVSAISVAYSIEIDVAKQDVKDFIQSLKKGGL------------------------ |
| *WP_084160254.1 (PbaB1) | WxxP | Bacillota | lasso | --------MIKNQALSLQDIVVQGKGNIVSDMGGEKVMLSVQNGKYYNIGEIGGIIWDAIEEPIAVKQLVANLVADYEVDQSECEQQVISFLTHLMDEKLIGVENQWAI--------------- |
| *WP_258356003.1 | WxxP | Bacillota | lasso | --------MSNKQAISLKLIVSQSAGNLVSDMDGEKVMLNIDKGKYYNIGQLGGVIWELVEQPMAVQELVSTLCSLYQVEQSECENQVIEFLEKLMQEGLIQVKNFSS---------------- |
| WP_114069283.1 | WxxP | Bacteroidota | lasso | -----------------MAKFIRKNETISGQLNDDLVMVDIEKGSYFSINSVATRIWELLENPLSPESLYDALLAEYDVTPEQCRTEVNEYLEKMKELGLIQVVV------------------- |
| WP_249219862.1 | YxxP | Bacteroidota | lasso | -------------MIFSGKEIKVSENSVVREMGAGIVILNLNTERFYEINEVGKRFWELLSDNHDYTSILNILQAEYEVSAEQLQDDITRLIGDLDEAELIVGY-------------------- |
| MCH8744259.1 | YxxP | Chloroflexota | lasso | ------MTVRYKSLELDKLTVTIPQEVLFQDLGEETVLLNVATGKYHGINGVGSRIWELIQESNPMEMVLATLLDEFEVSSKTLEDDLSQFLGVLQSKGLIEIHEADGQ--------------- |
| NMF83048.1 | YxxP | Cyanobacteriota | lasso | --------MPSQLDITSTTTLKATSNQVSSEVGEEVVILQLQSGQYFGIDGVGAVVWEKLQTPVTPTELEAGLINEFDVEPEVLRHDLQVLIQDLAAAGLVDVKGES----------------- |
| PYN02361.1 | YxxP | Euryarchaeota | lasso | --------MAPMSRPTLASRVTLNSDIAFRELDGELVILNLETGIYFGIDPVGARTWTLIEHHGSLGAVLEVLCSEYDAPPAVLERDLLELVDQLCAKGLTRVAASSA---------------- |
| WP_245259933.1 | YxxP | Pseudomonadota | lasso | --------------------MSASKDAVACEFGNGLALLDMRSNIYYSINSVGAYIWELIQEPRPISEIRSAVLDRYNVDPERCKADVDGLLKGLADAGLARLHDEELV--------------- |
| WP_092309227.1 | WxxP | Pseudomonadota | PQQ | --------MSFDRSK--TPRWRPGYRFQYEPAQKGHVLLYP--EGMIKINDSAALIGGLIDGERNVAAIISELEVQFPGV-AELGDDIEQFMEVARAQHWIELG-------------------- |
| WP_409525499.1 | WxxP | Pseudomonadota | PQQ | MSASQTVDARAIDAR--AIRLNPMFRLQWEAAQDAWVLLYP--EGMVKINATAAAILNHVDGKRSLAGIVQALQNDYPQA-EGLIDDVAAFMQEAHANNWVIYD-------------------- |
| WP_029196375.1 (LppB) | YxxP | Bacillota | LPP | -----------------MTQYLRMNDYESIQLDMEWIILNTDEYTLTKINGVGGFCWSLLGAAQTVGSISEAIRKEYEFVNETVEEDIEAFLNDMIGRGLVQHAVS------------------ |
| | | | | 1 10 20 30 40 50 60 70 80 89 |

**Supplementary Figure 17. Multiple sequence alignment of selected RRE domains from different phyla.** Residues corresponding to N33 and D70 in LppB are shown in color. RREs from paeninodin-like lasso peptide BGCs are indicated with an asterisk. The LP column denotes the conserved RRE binding motif of the corresponding peptide precursors.

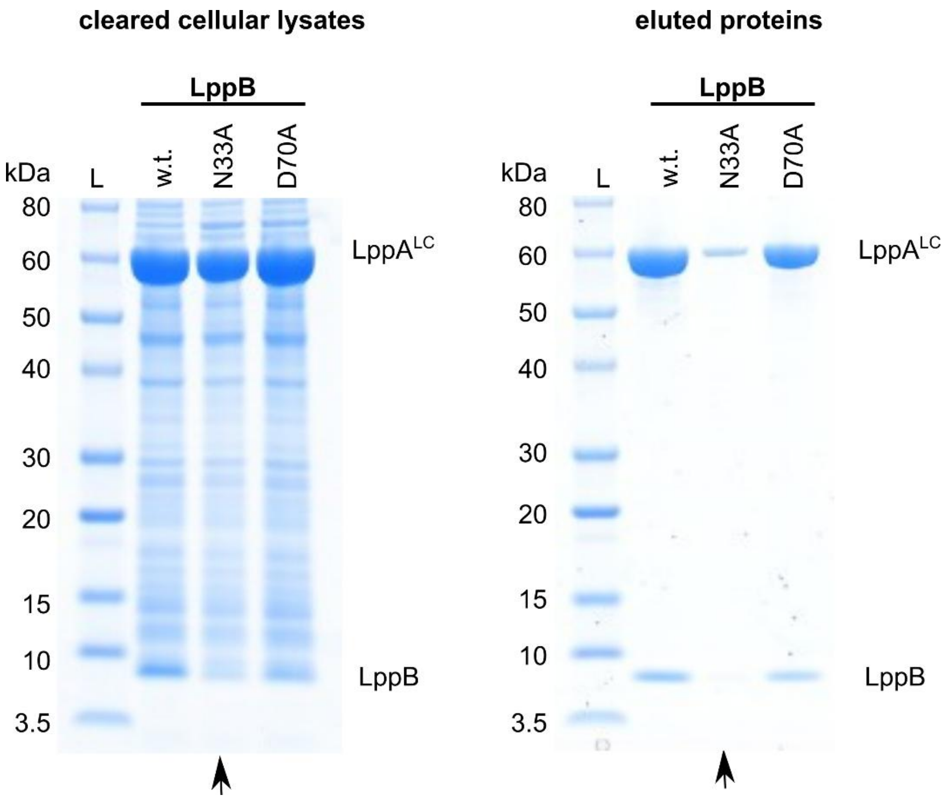


**Supplementary Figure 18.** Cleared cellular lysates (left) and eluted protein fractions (right) from pull-down assays of wild-type or mutant LppB in complex with the LppA$^{LC}$ peptide. LppA$^{LC}$ was expressed as an MBP–LppA$^{LC}$–TrxA fusion protein (MW 61.5 kDa). Arrows indicate samples in which LppB is largely insoluble in the absence of a bound peptide and therefore reduced in the cleared lysate, resulting in a reduced amount of the recovered complex. L, molecular weight ladder. All pull-down experiments were performed independently three times with similar results. Source data are provided as a Source Data file.

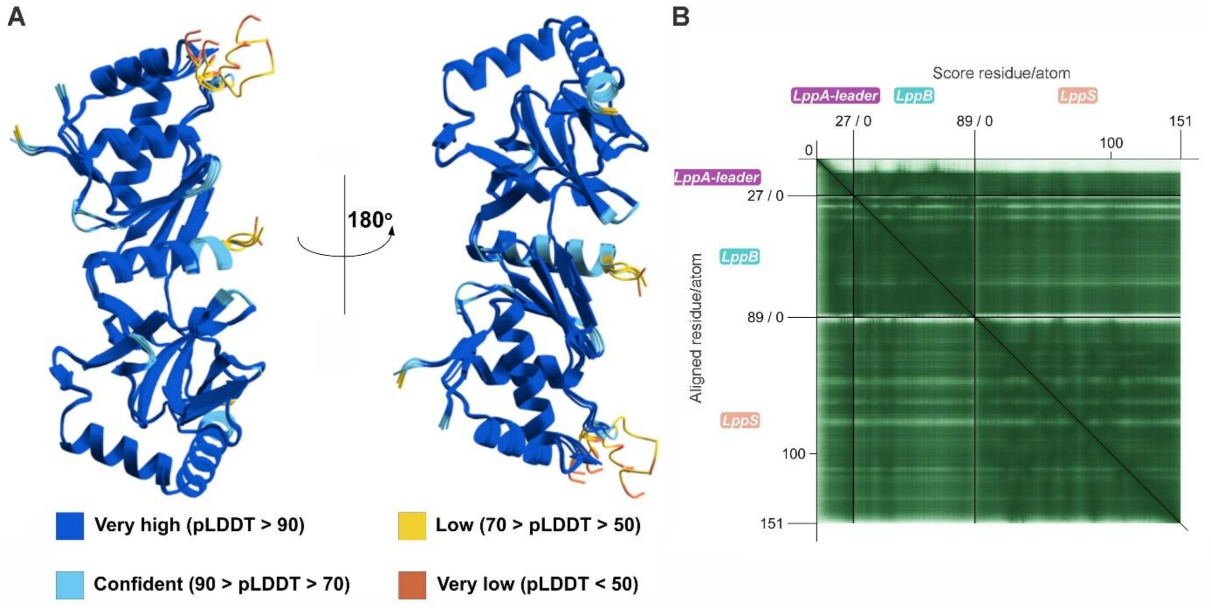


**Supplementary Figure 19. AlphaFold3**[2] **predicted structure of the LppS•LppB•LppA$^{L}$ complex**. **A.** Five generated models of the complex overlapped and colored according to the pLDDT score. **B.** Predicted Aligned Error (PAE) plot generated by PAE Viewer[3] online at https://pae-viewer.uni-goettingen.de/.

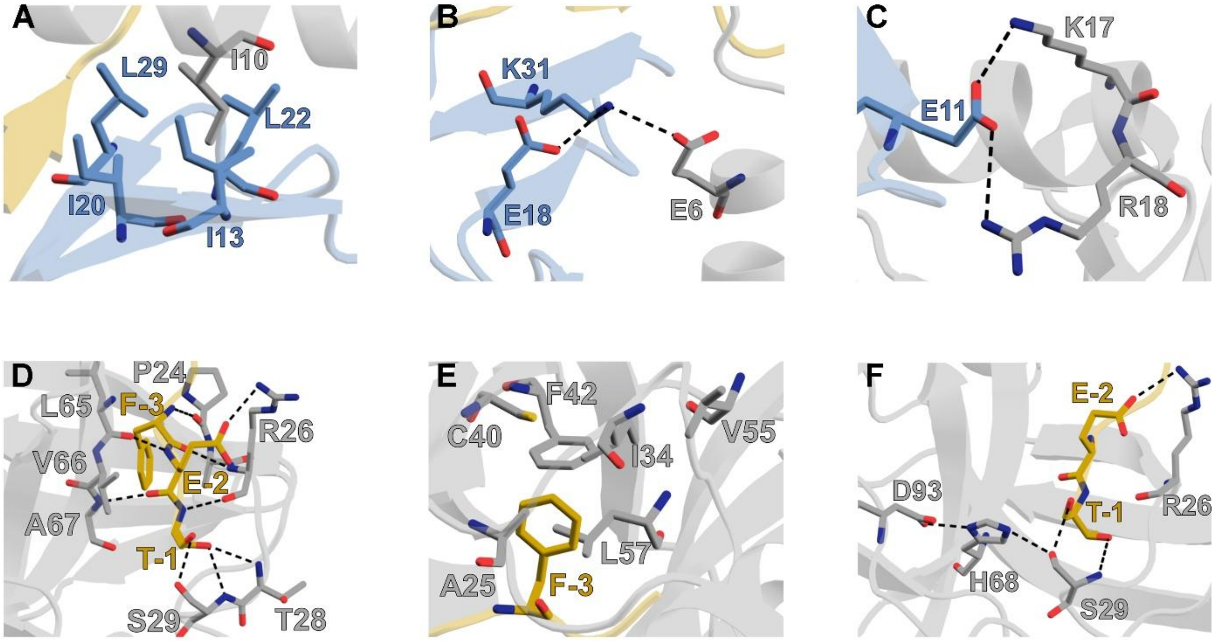


**Supplementary Figure 20. Intermolecular interactions in the LppS•LppB•LppA$^{L}$ complex predicted by the AlphaFold3[2] structural model.** LppA$^{L}$ is shown in yellow, LppB in blue, and LppS in grey. Hydrogen bonds are indicated with black dashed lines.

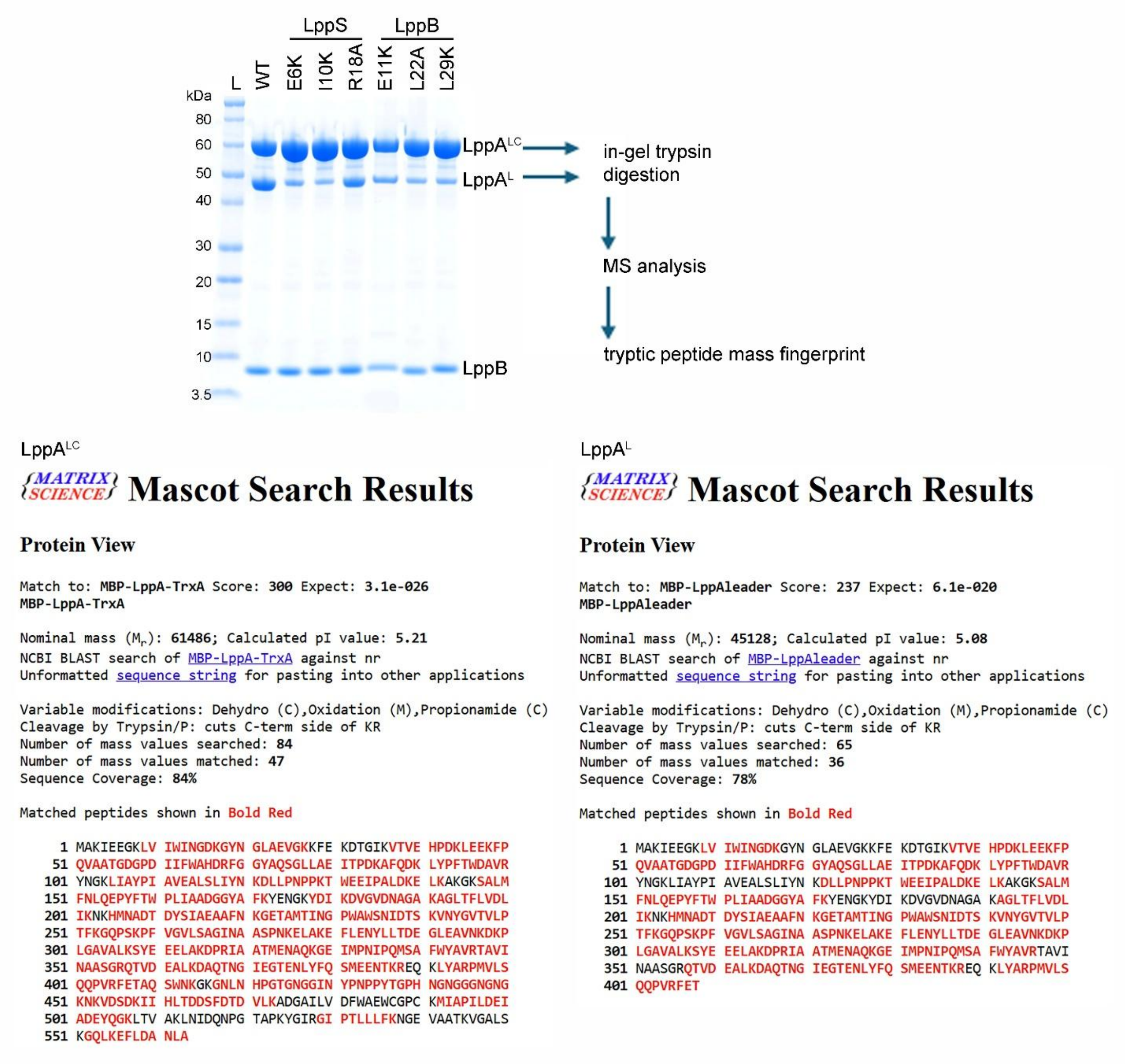


**Supplementary Figure 21. Mutational analysis of the LppB–LppS interaction interface in the *in vivo* cleavage/pull-down assay.** Mutations in the corresponding proteins are listed at the top. L, protein MW ladder, $LppA^{LC}$, MBP-$LppA^{LC}$-TrxA fusion (MW 61.5 kDa), $LppA^{L}$, $LppA^{L}$ leader peptide fused to MBP (45.1 kDa). Representative tryptic peptide fingerprint analyses confirming correct $LppA^{LC}$ cleavage are shown in the bottom panels. All pull-down experiments were performed independently three times with similar results. Source data are provided as a Source Data file.

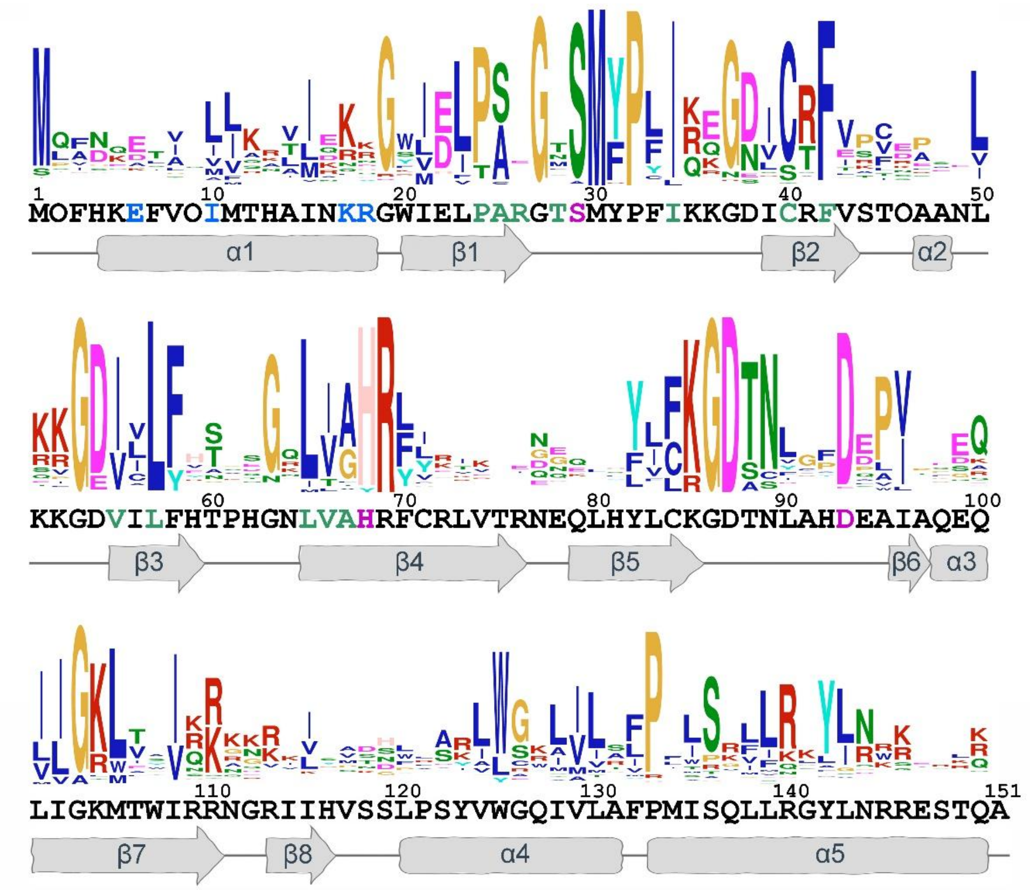


**Supplementary Figure 22. The LppS secondary structure and sequence conservation.** The conservation of residues is shown as Logo[4]. Secondary structure elements predicted by AlphaFold3 structural model[2] are depicted as arrows (β strands) and rectangles (α helices). Amino acids forming LppS/LppB and LppS/LppA$^{LC}$ intermolecular interfaces are shown in blue and green, respectively. Catalytic residues are in magenta.

**A**

```
LppK  1    MELFHTQIGEHAIQLICQSEALMC--------------IMRKNFPAADLIVSKPDII---------------IYI---KE  48
BsfK  1    MENTDRLVRYRAFGRDIDGPAGLSlspaPAEAAPGEVPVLRLQ-PDASLRVFIDENLPPLLHNVEDYPGgpKFYVwqEGE  79
PbaK  1    MIEVEKKVVYKAFGLTI-----------VSDVALPELTIVDEQIEEIDIEIKKDEDLTRLYFELTAQPN--QFVV--KDH  65

LppK  49   GYGISFVDYDVEITKEVDSISFRRADYLIEAELDYRYAVISVNN---ELALKHALMNMYSSYVVHHkwGLLIHSSCVMDK  125
BsfK  80   AVGVQYDRWRTRLIPGEGRIDFAELPPS--GPKRVEDAGDEYGRfrfSLAMERVFLPLYALFSMPD--AVALHGSAVVLN  155
PbaK  66   LVIFHIPEIATFSIKEGKRITFSPMKETKDGEIRLLILGTCMG----VILMQRKIFP-------------LHGSLVAIQ  127

LppK  126  GKAYLFAGHSGAGKSTAAKLSMPRN--LLSDEATVVKITSD-KVIIFNSPFRSELDRTT---GAEENSPLAGIYLLNQAV  199
BsfK  156  GEAFLFIGRSGAGKSTTAYEFVRRGATLLADDLIVADVA---RGIALGGAPTLRLWKGE--GALPEAQEDRSLW-RHDAS  229
PbaK  128  GKAYAIIGDSGAGKSTLASAFLNEGYQLLSDDVIAVWLSGEnMPYVTSSYPQQKLWQDSltNFGMESSEYQSLYGRVD--  205

LppK  200  DNHIVPLSKS-----------------------------------------NGFLHLMDKVFYWSH-SPEEIGGILQLL  236
BsfK  230  KRWFRIPAERGAASAVPIAAIVML----DPDTLGGQRDVLPGLET---SPQRKALTDLLGQTFDLSHgTPEWMVARFRNT  302
PbaK  206  -KYYIPVSSKYFTGSLPLAGVIELvkteDKEIAIRQIAKLERFKTlfyHTYRNFLIPDLGLT--------EW---HFNTS  273

LppK  237  QQLVNSVSICELHFQKN--------DTFWELIS-----  261
BsfK  303  ARLIREYPFYHFRYVKSADGKPTHMDALYQAIVGLAsk  340
PbaK  274  VSIVNNVQMFQLSRPASGFTAPQLVTTIMETLNLEA--  309
```

**B**

```
LppN  1    MI----------------ITLIQALYDpRVPIPQEIEFFDK----------ALEDIEFFDISPQIYWLLKQRGQlVQMPP  54
PbaN  1    ME---NDFRSDSMHFSKELTLLLSIM--RMDKGESMLWKDQDQaneidwGAFLQLARHHRVFSLLYTNLRQTDS-AWIPT  74
BsfN  1    MNapcNEKLASTRLFPEDCPTTSLVLE-RIVEGSALCAGDIAH------ERLLTDADAAGFLPVLYRYLLSTGR------  67

LppN  55   FFQEQLQQKYNGTIYQNMFIRSQTKIALEKLEEAGIQTIPLKGTFFAEKYFGHLGARGTSDIDLLVQPFDLERAIHCVKS  134
PbaN  75   YVMNALQKDYQENTFNMLRLSAEMEQICKLFSESDIRTIVLKGPVLADDLYGNISLRTSRDLDILIPIQHVDQAHELLRS  154
BsfN  68   DVPGRFRQSWLVHIARLALYRAELRRVLPIIEAFS-PVVLLKGEGLSLLLFGDARLRNTTDMDLLIDPRQLGPIVEALGE  146

LppN  135  LGYIEQERIPS--------HFHWSFSKEiPESPIPLTIELHWDLMKEKTSDINMDEFWEQATAIGSYRY---IKELSDYH  203
PbaN  155  QGYVKEEEYNTIlddwKWRHHHATFIH--PDKRV--TLEIHWRLGPGPGKEPGFDELWERKRISKITSH--PVYYLGRED  228
BsfN  147  IGYHTLGDGKTK----PWAYNQLLLVHE----DCGTLIELHWRIAFPHLKSPPIGDLLEDTIAVELDGGalPARSLRPEL  218

LppN  204  TFYMICLHGWRHNLCSLKYFLDIIQMIHILQDKIDYIRLQKDAATHKT---LRRTVRTLAIVYH---------------  264
PbaN  229  LFLFLASHGARHGWSRLRWLIDMDRITRQNIDYAKLHTLLTKNRQLHL---GAQAMILAAQLLHtpLTAEMESMTTGKRA  305
BsfN  219  LLLQLCFHFHQHH-GFYKGLLDIAGWIDRFESTADLDEVRALTRRYEIdgvLQWGLHALERFTG--VRSRLYDPHANLFA  295

LppN  265  ---------HFPHLAGIKELPIKKGINLWWQ------------YNAIRDRNYKTYIQYVNWVYYEF-FDFDSLAHTWAAL  322
PbaN  306  KRLAADALFYIRQMVNLHTAPVPEHVSKYHK------------KHLFSLMSAQQKILFVLSFFYPYpEDADTL--TLPKR  371
BsfN  296  KSWAAWSALEMERRYIFMASP--NAIDRWWRdprvttqiagvlADALSMTVADGALNRLRAVLSPV-----LLGPHRVGR  368

LppN  323  VNFTYS-----IGNMRLSHKIKKM---  341
PbaN  372  LHFLYFPlrpVLWAWRKTNSISQRGT-  397
BsfN  369  VNFAILE---GIGAFGREELYERRILg  392
```

**Supplementary Figure 23. Alignments of kinases (A) and NTP transferases (B) from *lpp*, *pba*, and *bsf*[5] BGCs.** Identical residues are highlighted in red.

**Supplementary Table 1. Data collection and refinement statistics**

| Name | LppB•LppA$^L$ | PbaB1•PbaA$^L$ |
|---|---|---|
| PDB ID | pdb_00009x8z | pdb_00009x90 |
| **Data collection** | | |
| Beamline | PF BL5-A | SPring8 BL32XU |
| Wavelength (Å) | 1.00 | 1.00 |
| Space group | $P\ 2_12_12_1$ | $P\ 4_3$ |
| Unit cell parameters:<br>a; b; c (Å)<br>α; β; γ (°) | <br>52.90; 55.60; 83.87<br>90; 90; 90 | <br>49.79; 49.79; 135.01<br>90; 90; 90 |
| Resolution (Å) | 50.00 – 2.10 (2.15 – 2.10) | 50.00 – 2.00 (2.05 – 2.00) |
| $CC_{1/2}$ | 99.80 (74.40) | 99.70 (79.90) |
| I/σ | 11.69 (1.79) | 11.12 (2.56) |
| Completeness (%) | 98.20 (97.10) | 99.10 (99.80) |
| Unique reflections | 27534* (2023*) | 21968 (22169) |
| $R_{meas}$ (%) | 13.50 (104.60) | 21.10 (118.50) |
| **Refinement statistics** | | |
| Resolution (Å) | 32.85 – 2.10 | 46.71 – 2.00 |
| Reflections | 27532* | 21957 |
| $R_{work}$ | 0.20 | 0.23 |
| $R_{free}$ | 0.25 | 0.26 |
| Number of atoms (total) | 1814 | 1959 |
| Number of protein atoms | 1720 | 1685 |
| Number of water atoms | 94 | 274 |
| **RMSDs** | | |
| Bond length (Å) | 0.007 | 0.007 |
| Bond angles (°) | 0.943 | 0.852 |
| **Ramachandran plot** | | |
| Outliers (%) | 0.00 | 0.00 |
| Allowed (%) | 1.95 | 0.96 |
| Favored (%) | 98.05 | 99.04 |

Values in parentheses are for the high-resolution shell

*Anomalous data

**Supplementary Table 2. Synthetic DNA fragments used in the study**

| DNA fragment ID | Manufacturer | Sequence |
|---|---|---|
| lppA$^{LC}$-sfGFP | Thermo Fisher | gcgaattaatacgactcactatagggcttaagtataaggaggaaaaaatatgaaaataaaaacaggagcacgcaataacatggaagaaaatacgaagagggaacaaaaattgtatgcgcgccccatggttttaagtcaacaacccgttcgatttgagacagctcaaagctggaataagggggaaaggtaacctaaatcacccgggtacagggaatggcggcattaattatccgaaccctccgtacaccggaccgcataatggtaacggaggcggcaacggtaacggtaagaataagcatcaccatcatcaccactccgcggctcttgaagtcctctttcagggacccatgagcaaaggagaagaacttttcactggagttgtcccaattcttgttgaattagatggtgatgttaatgggcacaaattttctgtccgtggagagggtgaaggtgatgctacaaacggaaaactcacccttaaatttatttgcactactggaaaactacctgttccatggccaacacttgtcactactctgacctatggtgttcaatgcttttcccgttatccggatcacatgaaacggcatgactttttcaagagtgccatgcccgaaggttatgtacaggaacgcactatatctttcaaagatgacgggacctacaagacgcgtgctgaagtcaagtttgaaggtgatacccttgttaatcgtatcgagttaaaaggtattgatttaaagaagatggaaacattctcggacacaaactcgagtacaactttaactcacacaatgtatacatcacggcagacaaacaaaagaatggaatcaaagctaacttcaaaattcgccacaacgttgaagatggttccgttcaactagcagaccattatcaacaaaatactccaattggcgatggccctgtccttttaccagacaaccattacctgtcgacacaatctgtcctttcgaaagatcccaacgaaaagcgtgaccacatggtccttcttgagtttgtaactgctgctgggattacacatggcatggatgagctctacaaaggtagctaataaggaccgaattctgtacaggcc |
| trxA-pbaA$^{LC}$ | Thermo Fisher | agcgataaaatcatccatctgaccgatgatagctttgataccgatgttctgaaagcagatggtgcaattctggttgatttttgggcagaatggtgtggtccgtgtaaaatgattgcaccgattctggatgaaatcgcggatgaatatcagggtaaactgaccgttgcaaaactgaacattgatcagaatccgggtacagcaccgaaatatggtattcgtggtattccgacactgctgctgtttaaaaacggtgaagttgcagcaaccaaagttggtgcactgagcaaaggtcagctgaaagaatttctggatgccaatctggcaggcaccgaaaatctgtattttcagagcatgaaaaaagaatggttgaaacctgagcttgaagtattggatgttaatcaaacaatgcttgatccgaaaaatggtaaccaccttgatcatgcttatacggaaggtactcctaaagacaaattaacatggagctaa |

## Supplementary References


1. Willard, L. *et al*. VADAR: a web server for quantitative evaluation of protein structure quality. *Nucleic Acids Res*. **31**, 3316–9 (2003).

2. Abramson, J. *et al*. Accurate structure prediction of biomolecular interactions with AlphaFold 3. *Nature* **630**, 493–500 (2024).

3. Elfmann, C. & Stülke, J. PAE viewer: a webserver for the interactive visualization of the predicted aligned error for multimer structure predictions and crosslinks. *Nucleic Acids Res*. **51**, W404–W410 (2023).

4. Crooks, G. E., Hon, G., Chandonia, J.-M. & Brenner, S. E. WebLogo: A Sequence Logo Generator. *Genome Res*. **14**, 1188–1190 (2004).

5. Duan, Y. *et al*. Leader peptide removal in lasso peptide biosynthesis based on penultimate isoleucine residue. *Front. Microbiol*. **14**, 1181125 (2023).
