# Supplementary material for: RiPP recognition elements evolved to prevent pathway interference through leader peptide discrimination
Source: Nat Commun. 2026 May 20;17:6633. doi: 10.1038/s41467-026-73250-6 (PMC13381572; doi:10.1038/s41467-026-73250-6)
Supplement: Supplementary file 5 — Reporting Summary [file 41467_2026_73250_MOESM5_ESM.pdf]

nature portfolio

Corresponding author(s): Shunsuke Tagami
Svetlana Dubiley

Last updated by author(s): Jan 17, 2026

Reporting Summary

Nature Portfolio wishes to improve the reproducibility of the work that we publish. This form provides structure for consistency and transparency in reporting. For further information on Nature Portfolio policies, see our Editorial Policies and the Editorial Policy Checklist.

Statistics

For all statistical analyses, confirm that the following items are present in the figure legend, table legend, main text, or Methods section.

n/a	Confirmed	
☐	☒	The exact sample size (*n*) for each experimental group/condition, given as a discrete number and unit of measurement
☐	☒	A statement on whether measurements were taken from distinct samples or whether the same sample was measured repeatedly
☐	☒	The statistical test(s) used AND whether they are one- or two-sided<br>*Only common tests should be described solely by name; describe more complex techniques in the Methods section.*
☒	☐	A description of all covariates tested
☒	☐	A description of any assumptions or corrections, such as tests of normality and adjustment for multiple comparisons
☐	☒	A full description of the statistical parameters including central tendency (e.g. means) or other basic estimates (e.g. regression coefficient) AND variation (e.g. standard deviation) or associated estimates of uncertainty (e.g. confidence intervals)
☒	☐	For null hypothesis testing, the test statistic (e.g. *F*, *t*, *r*) with confidence intervals, effect sizes, degrees of freedom and *P* value noted<br>*Give P values as exact values whenever suitable.*
☒	☐	For Bayesian analysis, information on the choice of priors and Markov chain Monte Carlo settings
☒	☐	For hierarchical and complex designs, identification of the appropriate level for tests and full reporting of outcomes
☒	☐	Estimates of effect sizes (e.g. Cohen's *d*, Pearson's *r*), indicating how they were calculated

Our web collection on statistics for biologists contains articles on many of the points above.

Software and code

Policy information about availability of computer code

Data collection	BLASTP; Bruker Topspin (v. 4.1.3); Bruker hsqcf3gpph19
Data analysis	EFI-EST (v. 2025_03 / 106); Cytoscape (3.10.4); RODEO (2.1.3); Clustal Omega (v. 1.2.4); MEME (v. 5.5.8); WebLOGO (v. 2.8.2 and v. 3.7.3); MUSCLE5 (v.5); iTOL (v.7); FastTree (v. 2.2); MMSEQS2 (v2); AlphaFold2 and AlphaFold3; FlexAnalysis (v. 3.2); MassHunter Qualitative Analysis (v. 10.00); PAE Viewer (https://pae-viewer.uni-goettingen.de/); VADAR (v. 1.3); XDS (v. Jan 31, 2020 and v. Jan 10, 2022); PHENIX (v. 1.21-5207); PHASER (v. 2.8.3); PyMOL (v.3.1.0); COOT (v. 0.9.8.92). A Python script for calculation of domain co-occurence is available at https://github.com/bikdm12/lpp.

For manuscripts utilizing custom algorithms or software that are central to the research but not yet described in published literature, software must be made available to editors and reviewers. We strongly encourage code deposition in a community repository (e.g. GitHub). See the Nature Portfolio guidelines for submitting code & software for further information.

Data

Policy information about availability of data

All manuscripts must include a data availability statement. This statement should provide the following information, where applicable:

- Accession codes, unique identifiers, or web links for publicly available datasets
- A description of any restrictions on data availability
- For clinical datasets or third party data, please ensure that the statement adheres to our policy

Protein and genomic sequences analyzed in this study are available in the NCBI Protein (https://www.ncbi.nlm.nih.gov/protein/) and Nucleotide (https://

www.ncbi.nlm.nih.gov/nucleotide/) databases, respectively. Identified protein domains are available in the Pfam (http://pfam.xfam.org/) and TIGRFAMs (https://tigrfams.jcvi.org/cgi-bin/index.cgi) databases. Accession numbers are listed in Supplementary Dataset 1. Data from the previous study by Kretsch et al. used in this work are available in 10.1021/acs.biochem.2c00700. Completely sequenced bacterial genomes were downloaded from the NCBI FTP site (https://ftp.ncbi.nlm.nih.gov/). The NMR data and raw data supporting the phylogenetic analysis are available on Zenodo (https://doi.org/10.5281/zenodo.18877617). Atomic coordinates of LppB•LppAL and PbaB1•PbaAL complexes have been deposited in the Protein Data Bank (PDB) under accession codes pdb_00009x8z (https://doi.org/10.2210/pdb9X8Z/pdb) and pdb_00009x90 (https://doi.org/10.2210/pdb9X90/pdb). Atomic coordinates of FusB1•FusAL(TfuB1•TfuAL), TbiB1•TbiAαL, and TbiB1•TbiAβL discussed in this study are available under accession codes pdb_00005v1v (https://doi.org/10.2210/pdb5v1v/pdb), pdb_00006jx3 (https://doi.org/10.2210/pdb6jx3/pdb), and pdb_00005v1u (https://doi.org/10.2210/pdb5v1u/pdb), respectively. Relevant raw MALDI-TOF mass spectra are available at https://repository.jpostdb.org/entry/JPST004185.0.

Research involving human participants, their data, or biological material

Policy information about studies with human participants or human data. See also policy information about sex, gender (identity/presentation), and sexual orientation and race, ethnicity and racism.

Reporting on sex and gender	No human participants were involved in the study, and no human data was used except for the researchers themselves and the data they generated
Reporting on race, ethnicity, or other socially relevant groupings	No human participants were involved in the study, and no human data was used except for the researchers themselves and the data they generated
Population characteristics	No human participants were involved in the study, and no human data was used except for the researchers themselves and the data they generated
Recruitment	No human participants were involved in the study, and no human data was used except for the researchers themselves and the data they generated
Ethics oversight	No human participants were involved in the study, and no human data was used except for the researchers themselves and the data they generated

Note that full information on the approval of the study protocol must also be provided in the manuscript.

Field-specific reporting

Please select the one below that is the best fit for your research. If you are not sure, read the appropriate sections before making your selection.

[x] Life sciences [ ] Behavioural & social sciences [ ] Ecological, evolutionary & environmental sciences

For a reference copy of the document with all sections, see nature.com/documents/nr-reporting-summary-flat.pdf

Life sciences study design

All studies must disclose on these points even when the disclosure is negative.

Sample size	Sequences for the bioinformatical analysis were collected from the NCBI protein database (February 2019) using BLASTP search for WP_029196374.1 homologs with an E-value cutoff of 0.001 (n=240)
Data exclusions	Short (less than 3 kb) and truncated (containing long NNN regions that prevented analysis of coding sequences) contigs were manually removed from the original dataset (n=41)
Replication	All biochemical experiments were performed in 3 replicas with similar results
Randomization	Not applicable
Blinding	Not applicable

Reporting for specific materials, systems and methods

We require information from authors about some types of materials, experimental systems and methods used in many studies. Here, indicate whether each material, system or method listed is relevant to your study. If you are not sure if a list item applies to your research, read the appropriate section before selecting a response.

Materials & experimental systems

n/a	Involved in the study	
☒	☐	Antibodies
☒	☐	Eukaryotic cell lines
☒	☐	Palaeontology and archaeology
☒	☐	Animals and other organisms
☒	☐	Clinical data
☒	☐	Dual use research of concern
☒	☐	Plants

Methods

n/a	Involved in the study	
☒	☐	ChIP-seq
☒	☐	Flow cytometry
☒	☐	MRI-based neuroimaging

Plants

Seed stocks	no plants were used in the study
Novel plant genotypes	no plants were used in the study
Authentication	no plants were used in the study
