# Supplementary material for: RiPP recognition elements evolved to prevent pathway interference through leader peptide discrimination
Source: Nat Commun. 2026 May 20;17:6633. doi: 10.1038/s41467-026-73250-6 (PMC13381572; doi:10.1038/s41467-026-73250-6)
Supplement: Supplementary file 6 — Transparent Peer Review file [file 41467_2026_73250_MOESM6_ESM.pdf]

nature portfolio

Peer Review File

RiPP Recognition Elements Evolved to Prevent Pathway Interference through Leader Peptide Discrimination

Corresponding Author: Dr Svetlana Dubiley

This file contains all reviewer reports in order by version, followed by all author rebuttals in order by version.

Version 0:

Reviewer comments:

Reviewer #1

(Remarks to the Author)
The manuscript "Rules of Coexistence: RiPP Recognition Elements Evolved to Prevent Pathway Interference through Leader Peptide Discrimination" by Dubiley and colleagues describes the discovery of a new type of ribosomal natural product (RiPP), termed linear polyphosphorylated peptides. The biosynthetic gene clusters (BGCs) for this new type of RiPP were discovered through their co-occurence with lasso-peptide BGCs, which are also RiPPs. In RiPP biosynthesis, certain enzymes may contain so-called RiPP recognition elements, which bind to the substrate, a ribosomally produced precursor peptide. In addition to the discovery of this new RiPP family, the authors also investigated how the biosynthetic enzymes of the two RiPP BGCs that co-occur distinguish between their respective precursor peptides.
Overall, this is an intriguing manuscript well-worth reading. This reviewer has certain concerns that ought to be addressed before publication in NatComms:

Major:
1. This reviewer fully appreciates the difficulties associated with the discovery of a novel natural product, especially a RiPP. Heterologous expression may lead to success, but as the authors themselves note, it is unclear if all modifications are in fact installed. Given the lack of apparent activity of the NTP-transferase, and the implications polyphosphorylation could play in the context, I would strongly recommend refraining from naming a new class of natural products, when the final product is (putatively) unknown. This should be reserved for a later time.

2. The extensive use of pull-down assays to ascertain preferred binders. The authors have investigated leader peptide preference of the RREs from the two co-localized RiPP pathways for their respective leader peptides purely qualitatively. While this is suitable for an initial description of the system and certainly called for in the co-expression studies employed here, it would be highly desirable to obtain quantitative data. At the end of the day, pull-down assays are so dependent on the person performing the experiment, that actual KDs ought to be used to delineate the relative preferences of the RREs. After all, it is possible that the differences are much smaller than one might expect based on the pull-down data, which would make for an even more interesting story.

Minor:
I dommend the authors for providing the actual pdb and mtz files! A cursory look at the statistics raises two minor questions: Why was the resolution cut-off used? The useable data appears to extend further. The Rfree appears to be quite high given the resolution. Is there a particular reason, other than that the data appears to be twinned? Which should be mentioned in the main text.

The authors state that discrete RREs exist in nearly 50% of all RiPP clusters. I would suggest to rephrase: RREs exist in nearly 50% of all RiPP clusters, some of which are discrete.

Figure 1 is not as clear as it could be, and especially the labelling of A1 and A2 could be improved. I suggest revising the figure to help the general audience of Nat Comms to ease into the subject.

I look forward to reviewing a suitably revised manuscript.

Reviewer #2

(Remarks to the Author)
The study presents the intriguing discovery of a new family of polyphosphorylated RiPP-like products (LPPs) and explores how closely related leader motifs are discriminated by distinct RRE domains. The topic is timely, and the underlying questions about substrate selection, pathway coexistence, and RRE specificity are of relevance to RiPP enzymology and peptide natural products. The manuscript would benefit from clarifying several points related to evolutionary framing, genomic occurrence, structural expectations, and experimental interpretation. These revisions would significantly strengthen the clarity and impact of the work.
Below, I outline suggestions intended to support the manuscript and help ensure the conclusions are well grounded and accessible.
1. Evolutionary framing and RRE ancestry. The title and abstract suggest new evolutionary insight, but these ideas are not fully developed. It would strengthen the manuscript to clarify:
• Whether phylogenetic patterns support any plausible ancestral relationships between LPP and lasso peptide RREs.
• Whether any evidence of paralogous duplication appears in the RRE clades examined.
• Whether the data suggest one family predates the other, or whether coexistence is better explained by later diversification.
2. Genomic co-occurrence of RiPP pathways. The introduction states that lasso peptide and PQQ pathways do not co-occur in the same genome. A quick survey shows these pathways do in fact co-occur in the same genome. Resolution of this discrepancy is important because the manuscript uses the absence of their co-occurrence as a conceptual foundation for the "conflict" model.
3. Prior observations of RiPP cross-pathway interactions. The idea that broad RiPP substrate tolerance could lead to pathway interference is compelling. There are, however, prior studies examining substrate competition and crosstalk in multi-enzyme RiPP pathways. Adding a short contextual note acknowledging previous work would situate the current study more clearly within the field.
4. Clarification of RRE leader-peptide recognition rules. The discussion emphasizes a canonical leader motif for lasso peptide RREs. Some lasso systems, particularly those where the RRE is fused to a peptidase domain, do not encode the motif cited in the manuscript.
5. Structural definition of the proposed RiPP class. The identification of a new family of precursor peptides with characteristic polyphosphorylation is intriguing. At present, the manuscript does not show chemical structures or clarify whether the detected phosphorylated products represent mature molecules or pathway intermediates. Should we be so-naming the RiPP class if we don't know it has phosphate in the final structure?
6. Interpretation of transcriptional and biochemical "conflict". The observation that relevant genes show modest expression during stationary phase raises interesting possibilities, but it is not clear whether these conditions reflect ecologically relevant contexts for the producing organism. It may be useful to comment on alternative explanations for how pathway coexistence could be managed in vivo, including transcriptional regulation, environmental triggers, or distinctions between productive and nonproductive protein interactions.
7. Interpretation of the RRE-binding experiments.The pull-down assays provide a useful first look at how leader variations affect binding. In several panels, the visible amount of LppB differs between lanes, which calls into question whether these experiments are conclusive. Repeating these experiments with consistent loading is mandatory. If possible, complementing these data with a quantitative method (e.g., binding analysis by SPR or BLI) would be preferrable.
8. Rationale for specific mutagenesis choices. It would help the reader to understand why residues such as D70 and N33 in LppB were selected for mutagenesis. Are these the only important positions?

Version 1:

Reviewer comments:

Reviewer #1

(Remarks to the Author)
I would like to thank the authors for the careful revision of their manuscript. I particularly appreciate the lengths the authors have gone to to determine KD values for the protein-PP interactions. It is unfortunate that the enzymes are poorly soluble in the absence of their respective leader peptides. I always wonder what that means for the logistics of compound production...
I recommend the manuscript be accepted without further revision.

Reviewer #2

(Remarks to the Author)
The authors have provide a satisfactory response to the prior review. This article is ready to be published in Nat Comm.

Open Access This Peer Review File is licensed under a Creative Commons Attribution 4.0 International License, which permits use, sharing, adaptation, distribution and reproduction in any medium or format, as long as you give appropriate credit to the original author(s) and the source, provide a link to the Creative Commons license, and indicate if changes were made.
In cases where reviewers are anonymous, credit should be given to 'Anonymous Referee' and the source.
The images or other third party material in this Peer Review File are included in the article's Creative Commons license, unless indicated otherwise in a credit line to the material. If material is not included in the article's Creative Commons license and your intended use is not permitted by statutory regulation or exceeds the permitted use, you will need to obtain permission directly from the copyright holder.
To view a copy of this license, visit https://creativecommons.org/licenses/by/4.0/

Reviewer #1 (Remarks to the Author):

The manuscript "Rules of Coexistence: RiPP Recognition Elements Evolved to Prevent Pathway Interference through Leader Peptide Discrimination" by Dubiley and colleagues describes the discovery of a new type of ribosomal natural product (RiPP), termed linear polyphosphorylated peptides. The biosynthetic gene clusters (BGCs) for this new type of RiPP were discovered through their co-occurence with lasso-peptide BGCs, which are also RiPPs. In RiPP biosynthesis, certain enzymes may contain so-called RiPP recognition elements, which bind to the substrate, a ribosomally produced precursor peptide. In addition to the discovery of this new RiPP family, the authors also investigated how the biosynthetic enzymes of the two RiPP BGCs that co-occur distinguish between their respective precursor peptides.

Overall, this is an intriguing manuscript well-worth reading. This reviewer has certain concerns that ought to be addressed before publication in NatComms:

>We thank the Reviewer for the positive evaluation of our manuscript and for the helpful suggestions.

Major:

1. This reviewer fully appreciates the difficulties associated with the discovery of a novel natural product, especially a RiPP. Heterologous expression may lead to success, but as the authors themselves note, it is unclear if all modifications are in fact installed. Given the lack of apparent activity of the NTP-transferase, and the implications polyphosphorylation could play in the context, I would strongly recommend refraining from naming a new class of natural products, when the final product is (putatively) unknown. This should be reserved for a later time.

>We fully agree with this concern and have revised the relevant sentences to emphasize that the term LPP is used solely for convenience and is not intended as a formal nomenclature proposal **(L63-66, L115-116, and the Abstract)**.

2. The extensive use of pull-down assays to ascertain preferred binders. The authors have investigated leader peptide preference of the RREs from the two co-localized RiPP pathways for their respective leader peptides purely qualitatively. While this is suitable for an initial description of the system and certainly called for in the co-expression studies employed here, it would be highly desirable to obtain quantitative data. At the end of the day, pull-down assays are so dependent on the person performing the experiment, that actual KDs ought to be used to delineate the relative preferences of the RREs. After all, it is possible that the differences are much smaller than one might expect based on the pull-down data, which would make for an even more interesting story.

>Thank you for raising this important point. Although all pull-down experiments were independently repeated at least three times with consistent outcomes, we fully agree that quantitative data are essential. However, LppB proved insoluble in the absence of its precursor, even after refolding attempts. To validate the pull-down assays, we focused on PbaB1. Notably, PbaB1 also exhibited only marginal stability in the absence of the peptide, as revealed by NMR analysis (**Fig. S15)**.

To obtain quantitative parameters for the PbaB1-leader peptide interaction, we initially attempted microscale thermophoresis. Unfortunately, the affinity of the RED-tris-NTA dye for 6×His-PbaB1 was weaker than 50 nM, precluding reliable measurement. We therefore employed NMR spectroscopy to assess the affinity of complex formation. Although technically demanding and less precise than some biophysical methods, NMR has a broad dynamic range, which proved advantageous for measuring PbaB1 interactions with non-cognate precursors. The NMR results fully support the conclusions drawn from the pull-down assays. These data have been incorporated into the revised manuscript (**L293–L331, Figs. 5D and S16**).

Minor:

I dommend the authors for providing the actual pdb and mtz files! A cursory look at the statistics raises two minor questions: Why was the resolution cut-off used? The useable data appears to extend further. The Rfree appears to be quite high given the resolution. Is there a particular reason, other than that the data appears to be twinned? Which should be mentioned in the main text.

>Indeed, Phenix.Xtriage analysis of the PbaB1•PbaAL crystals indicated the presence of twinning, while the LppB•LppAL crystals has translational non-crystallographic symmetry. These crystallographic features limited the achievable resolution cutoff and contributed to the relatively high R values during refinement. We have added this information to the **Methods section (L668-669, 679-680)**.

The authors state that discrete RREs exist in nearly 50% of all RiPP clusters. I would suggest to rephrase: RREs exist in nearly 50% of all RiPP clusters, some of which are discrete.

>Thank you for pointing out this inaccuracy of wording. We revised this sentence accordingly **(L40-43)**

Figure 1 is not as clear as it could be, and especially the labelling of A1 and A2 could be improved. I suggest revising the figure to help the general audience of Nat Comms to ease into the subject.

>We did our best to the readability of **Figure 1** and revised the corresponding legend to provide a clearer and more accurate explanation.

I look forward to reviewing a suitably revised manuscript.

Reviewer #2 (Remarks to the Author):

The study presents the intriguing discovery of a new family of polyphosphorylated RiPP-like products (LPPs) and explores how closely related leader motifs are discriminated by distinct RRE domains. The topic is timely, and the underlying questions about substrate selection, pathway coexistence, and RRE specificity are of relevance to RiPP enzymology and peptide natural products. The manuscript would benefit from clarifying several points related to evolutionary framing, genomic

occurrence, structural expectations, and experimental interpretation. These revisions would significantly strengthen the clarity and impact of the work.

>We are grateful to the Reviewer for the careful reading of the manuscript and for the valuable suggestions that helped improve it.

Below, I outline suggestions intended to support the manuscript and help ensure the conclusions are well grounded and accessible.

1. Evolutionary framing and RRE ancestry. The title and abstract suggest new evolutionary insight, but these ideas are not fully developed. It would strengthen the manuscript to clarify:

>We extended the ramifications of the evolutionary analysis in the DISCUSSION section, (**L444-452**).

• Whether phylogenetic patterns support any plausible ancestral relationships between LPP and lasso peptide RREs.

>Direct reconstruction of phylogeny of RRE sequences suggests that paeninodins and LPP emerged independently from lasso peptides. We included this in the discussion **(L437-440)** and as a Fig. 8.

• Whether any evidence of paralogous duplication appears in the RRE clades examined.

>There is very little to no evidence of paralogy, attributable to intragenomic duplication, at least in *Bacilli* genomes, that seem to be the “native home” of these clades. This said, distant paralogs, belonging to different RRE clades, seem to be readily acquired via horizontal gene transfer. The problem with this (which also applies to the question below on coexistence of systems) is that (as expected) the evolutionary history of these systems is extremely dynamic (as suggested by the patchy phyletic distribution and supported by more formal reconstruction). Usually such an evolutionary behavior is a result of strong, but transient selection pressure, typical for systems involved in “biological conflicts” (defense, offence, antibiotic production and resistance, etc.). In this evolutionary regime the ancestral signal is many times written over by more recent contingent events. We simply do not feel confident that the currently observed pattern is informative with respect to the more distant past.

• Whether the data suggest one family predates the other, or whether coexistence is better explained by later diversification.

>There exists an intriguing pattern of co-occurrence of paeninodins, LPP and other lasso peptides in *Bacilli*. But we feel that proper quantification and interpretation of these data go well beyond the scope of this paper and deserves treatment as a standalone research project (very briefly, simply counting concordant and discordant presence/absence of different classes of RREs in genomes is not informative as closely related genomes are not statistically independent from each other and any “naïve” cooccurrence statistics would grossly overestimate the significance of the observations. We would also like to disentangle the contingent “ecological” pressure from simply passing the ancestral state to the descendants. At the very least this would require a thorough analysis of the temporal co-occurrence dynamics rather than of static extant snapshot).

2. Genomic co-occurrence of RiPP pathways. The introduction states that lasso peptide and PQQ pathways do not co-occur in the same genome. A quick survey shows these pathways do in fact

co-occur in the same genome. Resolution of this discrepancy is important because the manuscript uses the absence of their co-occurrence as a conceptual foundation for the “conflict” model.

>Thank you for this valuable insight. We have revised the relevant sentence to incorporate this possibility (**L54-58**).

3. Prior observations of RiPP cross-pathway interactions. The idea that broad RiPP substrate tolerance could lead to pathway interference is compelling. There are, however, prior studies examining substrate competition and crosstalk in multi-enzyme RiPP pathways. Adding a short contextual note acknowledging previous work would situate the current study more clearly within the field.

>Thank you. We added the relevant references and corresponding text to the Introduction **(L49-51).**

4. Clarification of RRE leader-peptide recognition rules. The discussion emphasizes a canonical leader motif for lasso peptide RREs. Some lasso systems, particularly those where the RRE is fused to a peptidase domain, do not encode the motif cited in the manuscript.

>Thank you for bringing this inaccuracy to our attention. We have clarified throughout the manuscript that this motif is characteristic of lasso peptide leaders recognized by discrete RREs (e.g. **L47**)

5. Structural definition of the proposed RiPP class. The identification of a new family of precursor peptides with characteristic polyphosphorylation is intriguing. At present, the manuscript does not show chemical structures or clarify whether the detected phosphorylated products represent mature molecules or pathway intermediates. Should we be so-naming the RiPP class if we don’t know it has phosphate in the final structure?

>We agree with this statement. In the revised manuscript, we clearly indicate that the term LPP is used solely for convenience and is not intended to designate or formally name this new RiPP family **(L63–66)**.

6. Interpretation of transcriptional and biochemical “conflict”. The observation that relevant genes show modest expression during stationary phase raises interesting possibilities, but it is not clear whether these conditions reflect ecologically relevant contexts for the producing organism. It may be useful to comment on alternative explanations for how pathway coexistence could be managed in vivo, including transcriptional regulation, environmental triggers, or distinctions between productive and nonproductive protein interactions.

>Thank you for this suggestion. We have added the relevant note in the Introduction section (**L126-128**).

7. Interpretation of the RRE-binding experiments.The pull-down assays provide a useful first look at how leader variations affect binding. In several panels, the visible amount of LppB differs between lanes, which calls into question whether these experiments are conclusive. Repeating these experiments with consistent loading is mandatory. If possible, complementing these data with a quantitative method (e.g., binding analysis by SPR or BLI) would be preferrable.

>Thank you for bringing this important issue to our attention. All pull-down assays shown on the same gel were performed in parallel to minimize day-to-day variation. Each pull-down

experiment was independently repeated at least 3 times, yielding consistent results. For the revised manuscript, we selected gels with more balanced loading for presentation.

Since LppB is insoluble in the absence of its cognate peptide, variation in the amount of material loaded reflects differences in the level of soluble LppB–peptide complex present in the cell, consistent with reduced binding affinity. To clarify this point, we supplemented the gels displaying eluted fractions with the corresponding cleared lysates prior to the pull-down assays whenever unequal loading of eluted samples might appear ambiguous (**Figs. S12, S13, and S18**), and we added an explanation to the main text (**L248–251**). Cleared lysates were shown instead of insoluble fractions, as various amount of insoluble LppB was observed in all samples, which would further complicate interpretation.

To validate the informativeness of the pull-down assays, we also assessed the affinity of PbaB1 for different peptides (cognate wild type, the cognate W-15Y variant, non-cognate LppA, and the LppA Y-16W variant) using NMR spectroscopy. The NMR data are fully consistent with the pull-down results. These data are included in the revised manuscript (**L293–L331, Figs. 5D and S16**).

8. Rationale for specific mutagenesis choices. It would help the reader to understand why residues such as D70 and N33 in LppB were selected for mutagenesis. Are these the only important positions?

To explain the rationale for the selected mutation, we have added the relevant clarification (**L334–348**) and highlighted the amino acid residues forming the RRE–leader interface in **Fig. 6A**.
